# Climate-driven co-evolution of antimicrobial resistance and virulence in *Escherichia coli* on dairy farms: unraveling adaptive genetic signatures with novel SSCP-PCR

**DOI:** 10.1007/s11274-025-04616-z

**Published:** 2025-10-28

**Authors:** Fawzia A. El-Shenawy, Mohamed A. M. Kotb, Doaa M. Sharaf, Azza SalahEldin El-Demerdash

**Affiliations:** 1 Bacteriology unit, Animal Health Research Institute (AHRI), Agricultural Research Center (ARC), Tanta city, Giza, Egypt; 2 Biotechnology Unit, Department of Microbiology, Agricultural Research Center (ARC), Animal Health Research Institute (AHRI), Zagazig, 44516 Egypt

**Keywords:** Climate-driven antimicrobial resistance, SSCP-PCR genetics, One health zoonoses, Bovine *E. coli* adaptation

## Abstract

This study addresses a critical One Health challenge by investigating the epidemiological and genetic drivers of antimicrobial resistance (AMR) in *E. coli* from 290 clinical bovine samples. On Egyptian dairy farms, our findings revealed that while calf diarrhea peaked during the winter, a higher rate of multidrug resistance was consistently observed in isolates from the summer, directly linking seasonal pressures to AMR dissemination. Strikingly, a mastitis isolate was confirmed as the highly virulent *E. coli* O157:H7 serotype, harboring the Shiga toxin genes *stx1* and *stx2*, underscoring a direct and significant public health risk. To dissect the molecular basis of these trends, we pioneered the use of a novel Single-Strand Conformation Polymorphism Polymerase Chain Reaction (SSCP-PCR) assay on 33 selected isolates. This high-throughput approach revealed prevalent mutations in resistance genes (*blaTEM* and *gyrB*) and the virulence gene (*fimH*). Crucially, sequencing confirmed that mutations in the highly conserved *16S rRNA* gene significantly co-occurred with mutations in *blaTEM*, *fimH*, and *lacI*, providing compelling evidence for co-selected adaptive pathways and clonal expansion. Our research demonstrates that climate-driven environmental pressures fuel the co-evolution of AMR and virulence on farms, championing SSCP-PCR as a robust tool for tracking microbial evolution and advocating for integrated, molecularly-informed One Health strategies.

**Supplementary Information:**

The online version contains supplementary material available at 10.1007/s11274-025-04616-z.

## Introduction

The Anthropocene is characterized by an escalating crisis born from two interwoven global challenges: accelerating climate change and the alarming rise of antimicrobial resistance (AMR). Climate change is not merely altering weather patterns; it is profoundly restructuring microbial ecosystems, creating novel and intensified selective pressures that directly fuel the evolution and dissemination of antibiotic resistance (Zambrano 2023; Litchman 2025). Rising ambient temperatures, for instance, are known to enhance microbial metabolism, which in turn accelerates critical ecological and evolutionary processes, including bacterial growth, increased population doubling times, speciation, and crucially, mutation rates (Ali et al. 2023; Magnano San Lio et al. 2023). Beyond heat, other climate-induced environmental variations, such as shifts in precipitation patterns, altered nutrient availability, and increased osmotic stress, also impose significant pressures, forcing microorganisms to adapt rapidly (Zhao et al. 2024; Raza et al. 2025). This constant struggle for survival drives genetic variation, the very engine of evolution, through mechanisms such as spontaneous mutations and horizontal gene transfer (Baquero et al. 2021; El Damaty et al. 2023; El-Demerdash et al. 2023b; Megahed et al. 2023).

The widespread use of antibiotics in both medicine and farming contributes to the growing problem of antibiotic resistance, which reduces the effectiveness of standard treatments (Essawi et al. 2020; Muteeb et al. 2023; El-Demerdash et al. 2024). This challenge is particularly acute and financially devastating within the agricultural sector, where the intensive use of antibiotics in livestock, such as cattle, creates a powerful selective pressure. Bovine mastitis, a costly inflammatory disease of the mammary gland, and calf diarrhea, a major cause of morbidity and mortality in young livestock, are frequently associated with infections by diverse pathogens (Vlasova and Saif 2021; Elashkar et al. 2024; Tartor et al. 2024). Among these, *Escherichia coli* stands out as a highly adaptable and globally prevalent bacterium. While many *E. coli* strains are commensal gut inhabitants, the species’ remarkable genomic plasticity enables the emergence of virulent pathotypes (such as enterotoxigenic *E. coli* (ETEC), enterohemorrhagic *E. coli* (EHEC), enteropathogenic *E. coli* (EPEC) capable of causing severe clinical symptoms (Denamur et al. 2021; El-Sheikh et al. 2024; Nemati et al. 2025b; 2025a). These infections lead to significant financial losses for farmers, with the economic burden of mastitis alone estimated to be in the billions of dollars globally, through reduced milk yield, treatment costs, and premature culling (Heikkilä et al. 2018; Holko et al. 2019; El-Demerdash et al. 2023a). Furthermore, certain *E. coli* pathotypes present a substantial public health concern due to their zoonotic potential (Sebre et al. 2022; Chekole et al. 2023; Hashem et al. 2024).

The inherent adaptability of *E. coli* is due to its high mutation rate, which is considered rapid in a microbiological context, typically ranging from 1 to 2 × 10⁻³ mutations per generation per genome (Foster et al. 2015; Ebrahem et al. 2024). This genetic flexibility makes the bacterium particularly susceptible to rapid evolutionary changes under new selective pressures imposed by climate shifts. Understanding how these environmental changes translate into specific genetic alterations within resistance genes is paramount for developing effective prevention and control strategies.

Despite the growing body of evidence on the effects of climate on microbial ecosystems (Vincent 2010; Ranheim Sveen et al. 2024), there remains a critical knowledge gap: a lack of integrated, on-the-ground studies that directly link specific seasonal climate variables to genetic mutations in field isolates of common livestock pathogens. This study directly addresses this gap by investigating the impact of seasonal changes on genetic mutations within key antimicrobial resistance and virulence genes of pathogenic *E. coli* isolated from field cases of calf diarrhea and cattle mastitis.

To precisely identify these subtle yet significant genetic variations, we employed a novel application of the Single-Strand Conformation Polymorphism Polymerase Chain Reaction (SSCP-PCR) assay. While traditional sequencing methods are comprehensive, they can be costly and time-consuming for screening large numbers of samples (Ari and Arikan 2016). SSCP-PCR offers a high-throughput, cost-effective alternative for rapidly detecting subtle genetic variations (Hashim and Al-Shuhaib 2019), making it an ideal tool for this large-scale screening study. We designed novel primers to target five genes of significant biological importance: *blaTEM* and *gyrB* (encoding β-lactam and fluoroquinolone resistance, respectively), *fimH* (a key virulence factor for host cell adhesion), and *16S rRNA* and *lacI* (representing a core ribosomal gene and a metabolic gene, respectively).

Through this innovative molecular approach in the Biotechnology Unit, Animal Health Research Institute, Zagazig Branch, Egypt, our findings aim to illuminate the direct molecular link between environmental shifts and the rapid evolution of *E. coli*’s resistance and pathogenic potential. This research offers crucial, evidence-based insights essential for developing targeted surveillance and effective intervention strategies to safeguard both animal and public health within the complex dynamics of a changing global climate.

## Materials and methods

### Study design and sample collection

This cross-sectional study aimed to determine the prevalence and characteristics of *Escherichia coli* (*E. coli*) isolated from mastitis milk samples and rectal swabs of calves suffering from diarrhea. Samples were collected from various dairy farms located within the Garbia Governorate, Egypt. The study protocol and all animal procedures received prior ethical approval from the Agricultural Research Center Institutional Animal Care and Use Committee (ARC-IACUC), under approval number ARC-AHRI-44-24. Animal handling and sampling procedures were conducted in strict accordance with the ARRIVE guidelines to ensure humane treatment and responsible reporting.

A total of 290 samples were collected. This total comprised 170 milk samples obtained from cattle exhibiting clinical signs of mastitis. The diagnosis of clinical mastitis was confirmed on-farm by a veterinarian based on the presence of macroscopic signs in the udder (swelling, redness, pain) and in the milk (watery consistency, clots, or flakes). For seasonal comparison, 90 of these milk samples were collected during the summer season (June to September 2022) and the remaining 80 samples were collected during the winter season (November to February 2023–2024). Based on data from the Egyptian Meteorological Authority, the average daily temperatures during the summer period ranged from approximately 25 °C to 35 °C, with maximum daily temperatures frequently exceeding 40 °C. In contrast, the average daily temperatures during the winter period were approximately 10 °C to 15 °C. Weather data, including daily temperature and precipitation, for both sampling periods was obtained from the Egyptian Meteorological Authority to confirm seasonal climate differences. Milk samples were collected aseptically after thorough cleaning and disinfection of the teats to minimize external contamination.

In addition, 120 rectal swab samples were collected from calves (aged 1–3 months) presenting with clinical signs of diarrhea. These calf samples were equally distributed by season, with 60 samples collected during summer and 60 during winter. Rectal swabs were collected directly from the rectum using sterile cotton swabs.

All samples were immediately placed in sterile containers or transport media specific to their type (milk in sterile tubes, swabs in Amies transport medium). They were then transported in an ice box (maintained at 4 °C) to the laboratory within 2–4 h of collection to ensure optimal preservation of microbial viability for *E. coli* isolation.

### Sample processing and bacterial identification

Upon arrival at the laboratory, all samples were processed promptly to maintain microbial viability. Rectal swabs were directly inoculated into Nutrient Broth for enrichment, while milk samples were first concentrated by centrifugation. Following enrichment, all cultures were streaked onto MacConkey Agar and then Eosin Methylene Blue (EMB) Agar for presumptive *E. coli* identification. Final confirmation of isolates was performed using a series of standard morphological, biochemical, and serological tests. Isolates exhibiting characteristic growth patterns were subjected to a battery of conventional biochemical tests, including Indole, Methyl Red, Voges-Proskauer, and Citrate, consistent with well-established protocols for *E. coli* identification (Quinn et al. 2011; Donna and Kohlerschmidt 2021). Confirmed isolates were further characterized by serotyping for O and H antigens using a standard slide agglutination test (Denka Seiken Co. Ltd.).

### Antimicrobial susceptibility testing (AST)

Antimicrobial susceptibility testing was performed on all confirmed *E. coli* isolates using the Kirby-Bauer disk diffusion method on Mueller-Hinton Agar (Bauer et al. 1966). A standardized bacterial suspension (0.5 McFarland standard) was used to inoculate the plates. The following twelve antimicrobial agents were tested: Ampicillin (AMP, 10 µg) from the Penicillins class; Amoxicillin/Clavulanate (AMC, 20/10 µg) from Penicillins + Beta-lactamase inhibitors; third-generation Cephalosporins including Ceftazidime (CTZ, 30 µg) and Cefotaxime (CTX, 30 µg); Sulfamethoxazole/Trimethoprim (SXT, 1.25/23.75 µg) from Sulfonamides/Diaminopyrimidines; Fluoroquinolones such as Ciprofloxacin (CIP, 5 µg) and Levofloxacin (LEV, 5 µg); Aminoglycosides including Amikacin (AK, 30 µg), Gentamicin (GN, 10 µg), and Streptomycin (STR, 10 µg); Imipenem (IMP, 10 µg) from Carbapenems; and Tetracycline (TET, 30 µg) from the Tetracyclines class. The selection of these twelve agents was based on their frequent use in treating common bovine diseases, particularly clinical mastitis and calf diarrhea, within the Egyptian veterinary context. Plates were incubated aerobically at 37 °C for 18–24 h. After incubation, the diameter of the zone of inhibition around each antibiotic disk was measured to the nearest millimeter using a ruler. Interpretations of susceptibility (Susceptible, Intermediate, or Resistant) were made based on the standard breakpoint criteria specified by CLSI (2023). The Multiple Antibiotic Resistance (MAR) index was calculated for each isolate to quantify the extent of antimicrobial resistance. The MAR index, a value between 0 and 1, was determined by dividing the total number of antibiotics to which a particular isolate demonstrated resistance by the total number of antibiotics tested (Tambekar et al. 2006; Sadat et al. 2021). A MAR index greater than 0.2 is commonly considered indicative of a high-risk source of contamination, suggesting exposure to antibiotics. Isolates were categorized as Multidrug-Resistant (MDR) if they exhibited resistance to at least one antimicrobial agent in three or more distinct antibiotic classes (Magiorakos et al. 2012).

### DNA extraction

Genomic DNA was extracted from pure *E. coli* isolates using the QIAamp DNA Mini Kit (Qiagen GmbH, Hilden, Germany) following the manufacturer’s instructions. To ensure high purity and yield suitable for downstream PCR applications, extracted DNA samples were subjected to specific inclusion criteria. DNA concentration was measured using a NanoDrop spectrophotometer (NanoDrop Technologies, Wilmington, DE, USA). Only DNA samples with a concentration of at least 20 ng/µL and a purity ratio (A260/A280) between 1.8 and 2.0 were included in subsequent analyses. Extracted DNA samples meeting these criteria were stored at −20 °C until further use.

### PCR amplification of target genes for mutation analysis

To investigate the genetic mutations influenced by climatic changes within *E. coli* populations, specific gene fragments crucial for antibiotic resistance, virulence, and broader bacterial adaptation were amplified by Polymerase Chain Reaction (PCR). This amplification step prepared samples for subsequent Single-Strand Conformation Polymorphism (SSCP) analysis. The primers designed for the amplification of these targeted genes, along with their respective annealing temperatures and amplicon sizes, are detailed in Table 1.

**Table 1:** Novel primer sequences, amplicon sizes, and annealing temperatures for e*scherichia coli* gene amplification

Target gene	Primers sequences (5’−3’)	Annealing Temp. (T°C)	Amplicon size (bp)	Gene Accession no.
*16S rRNA*	CAACACAGTGCTGTCTGGTGG	60	236	X80721.1
	TGAGTTTTAACCTTGCGGCC			
*gyrB*	TTCGGTAGTAAACGCCCTGT	60	229	PQ656130.1
	GAACGACAACTCACGCAGAC			
*blaTEM*	GTGCACGAGTGGGTTACATC	58	173	MF594222.1
	GAATAGTGTATGCGGCGACC			
*fimH*	GTTACTCTGCCGGACTACCC	60	249	JX847135.1
	TAATCCCAGACTTACCGCCG			
*lacI* operon	CGCTGTTGTTGTGGGGTTAA	58	247	AY159365.1
	CTACACCATGAAAGCCGCTC			
*stx1*	TGCAGTTGATGTCAGAGGGA TATCTGCATCCCCGTACGAC	60	241	FR875153.1
*stx2*	ATTCTCCCCACTCTGACACC ACCAGAGATGCATCCAGAGC	60	217	MW276034.1

Each PCR reaction was performed in a total volume of 25 µL, comprising: 12.5 µL of 2X DreamTaq Green PCR Master Mix (Thermo Scientific, Waltham, MA, USA), 1 µM of each forward and reverse primer, 5 µL of extracted genomic DNA template (approximately 50–100 ng), and nuclease-free water (molecular biology grade) to reach the final volume.

PCR amplification was carried out in an Applied Biosystems Veriti 96-Well Thermal Cycler (Thermo Fisher Scientific, USA) using the following cycling conditions: an initial denaturation at 95 °C for 5 min; 35 cycles of denaturation at 95 °C for 30 s, annealing at T°C (Table 1) for 30 s, and extension at 72 °C for 1 min; followed by a final extension at 72 °C for 7 min.

PCR products were analyzed via 1.5% agarose gel electrophoresis in 1X TBE buffer. Gels were stained with 0.5 µg/mL ethidium bromide and visualized under UV light using a Bio-Rad ChemiDoc MP gel documentation system (Bio-Rad Laboratories, Hercules, CA, USA) to confirm the presence and expected size of the amplified fragments. A 100 bp DNA ladder (Cat No. SM0311, Thermo Scientific) served as a molecular weight marker.

### Single-strand conformation polymorphism PCR (SSCP-PCR) assay

The SSCP-PCR assay was performed on the amplified gene fragments (*16S rRNA*, *gyrB*, *lac* operon, *blaTEM*, and *fimH*) to detect genetic mutations or polymorphisms that might be influenced by climatic variations.

SSCP Sample Preparation: For each PCR product to be analyzed by SSCP, 10 µL of the purified amplified DNA was mixed with 10 µL of denaturation loading buffer. The denaturation loading buffer comprised [95% formamide, 10 mM NaOH, 0.05% bromophenol blue, and 0.05% xylene cyanol]. The mixture was thoroughly vortexed, briefly centrifuged, then denatured by heating at 95 °C for 5–10 min in a thermal cycler to ensure complete dissociation into single strands. Immediately following denaturation, samples were rapidly snap-cooled on ice for 5–10 min to prevent re-annealing and promote the formation of stable, sequence-dependent single-strand conformations.

#### *Electrophoresis*

 The denatured and snap-cooled samples were carefully loaded onto a non-denaturing polyacrylamide gel. The gel consisted of 10% polyacrylamide (19:1 acrylamide: bis-acrylamide ratio) prepared in 0.5X TBE buffer. Electrophoresis was performed using a temperature-controlled electrophoresis unit [Bio-Rad DCode Universal Mutation Detection System] at a constant voltage of 200 V at a precisely controlled low temperature of 15 °C, as temperature is critical for SSCP resolution, for 4–6 h or until the tracking dye reached X cm from the bottom. A known reference *E. coli* strain (ATCC 25922), confirmed to lack mutations, was included as a control on each gel for comparison.

#### ** Visualization**

After electrophoresis, the gel was stained using a silver nitrate-based staining protocol, according to established procedures specific for high-sensitivity SSCP visualization (Avinash et al. 2016). Stained gels were then visualized using a gel documentation system. The presence of mobility shifts in the single-stranded DNA bands, compared to the wild-type control, indicated the presence of mutations or polymorphisms within the amplified gene fragments.

### DNA sequencing and mutation analysis

Following the SSCP assay, all conventional PCR amplified gene fragments that exhibited altered single-strand conformation polymorphism (SSCP) patterns, indicative of potential mutations or polymorphisms, were subjected to Sanger sequencing to precisely identify the underlying genetic changes. The amplified fragment sizes sequenced were consistent with the expected amplicon sizes from the PCR, as detailed in Table 1. Prior to sequencing, these amplified PCR products showing the band shifts were meticulously purified using a QIAquick PCR Purification Kit (Qiagen GmbH, Hilden, Germany) according to the manufacturer’s instructions. This step was crucial for removing residual primers, nucleotides, and enzymes that could interfere with the sequencing reaction.

Purified PCR products were then sent to GATC Biotech, Germany for bidirectional Sanger sequencing, utilizing the same forward and reverse primers employed for the initial conventional PCR amplification.

For comprehensive sequence data analysis, raw sequence chromatograms were first subjected to a stringent quality control process. We ensured that all base calls met a minimum Phred quality score (Q-score) of 20, corresponding to a 99% accuracy rate, and visually inspected the chromatograms for sharp, unambiguous peaks and minimal background noise. Low-quality base calls and adapter sequences at the ends of the reads were trimmed using the BioEdit software package version 7.2.5 (Ibis Bioscience, Inc., Carlsbad, CA, USA). Forward and reverse reads for each sample were then assembled to generate high-quality consensus sequences, relying on the robust overlap from the bidirectional sequencing to confirm base accuracy. The resulting consensus sequences were meticulously aligned with corresponding wild-type or reference gene sequences (from NCBI GenBank) to facilitate the precise identification of point mutations (single nucleotide polymorphisms - SNPs), insertions, deletions, and other genetic variations through comparative analysis. The specific type (e.g., synonymous, non-synonymous/missense, nonsense, frameshift) and exact genomic location of each detected mutation were meticulously recorded. Phylogenetic analyses, where applicable, were conducted utilizing the MegAlign module for tree reconstruction of sequences by the Neighbor-joining technique, based on ClustalW, using MEGA software version 11 (Kumar et al. 2008).

### Statistical analysis

All quantitative data were expressed as mean ± standard deviation (SD). Categorical data, including prevalence rates and proportions (*E. coli* isolation rates, resistance patterns, and mutation prevalence), were presented as frequencies and percentages. Statistical analyses were performed using SPSS version 26 (IBM Corp, Armonk, NY, USA) and GraphPad Prism version 8 (GraphPad Software, San Diego, CA, USA). Descriptive statistics, such as frequencies and percentages, were calculated for *E. coli* isolation rates across different sample types and seasons. Associations between categorical variables, including season, *E. coli* isolation rate, specific resistance patterns, or mutation prevalence in tested genes (*16S RNA*, *gyrB*, *blaTEM*, *fimH*, *lacI*), were assessed using the Chi-square (χ2) test. For comparisons of quantitative data between two groups (inhibition zone diameters), Student’s t-test was employed. One-way Analysis of Variance (ANOVA) was used for comparisons involving three or more groups (inhibition zone diameters across antibiotic classes). When ANOVA yielded a statistically significant result, Tukey’s HSD test was applied for multiple pairwise comparisons. Before correlation analyses, variables were tested for normality using a Q–Q plot. Pearson correlation (r) was estimated to assess relationships between quantitative variables. A p-value of < 0.05 was considered statistically significant for all analyses. Figures and graphical representations were generated using GraphPad Prism version 8 (GraphPad Software, San Diego, CA, USA) and R software version 4.4.3 (Patil 2021) (https://www.r-project.org/). Specifically, complex heatmaps and correlation visualizations were created in R using the ggplot2 (Wickham and Winston 2019), pheatmap (Kolde 2012), and Hmisc (Harrell 2020) packages. All software and package references are provided in the reference list.

## Results

### Prevalence of E. coli isolates

Out of a total of 290 samples tested, 94 *E. coli* isolates (32.4%) were successfully recovered. These isolates were definitively confirmed as *E. coli* through characteristic Gram-negative, rod-shaped morphology on Gram staining and consistent biochemical profiles. The isolates were recovered from clinical mastitis milk samples (*n* = 49) and calf diarrhea rectal swabs (*n* = 45).

Further serotyping of the isolates revealed a diverse distribution. The most prevalent serotype was O26, accounting for 40.6% (13/32) of the typed isolates, followed by O128 at 21.9% (7/32). Serotypes O86, O78, and O124 each constituted 6.25% (2/32) of the isolates. The remaining serotypes, including O111, O44, O157, O114, O55, and O8, were each found in a single isolate, representing 3.1% of the total. A single isolate from a clinical mastitis sample was identified as the highly virulent *E. coli* O157:H7 serotype. This isolate was the only one subjected to PCR for toxin gene confirmation and was found to harbor the Shiga toxin genes *stx1* and *stx2*, underscoring a significant zoonotic and public health risk.

Seasonal analysis of isolation rates revealed a notable difference between sample sources. For clinical mastitis, the isolation rate was 33.3% (30/90) during summer and 23.75% (19/80) during winter, with no statistically significant difference between seasons (*p* = 0.227). Conversely, *E. coli* isolation from calf diarrhea rectal swabs showed a marked seasonal variation, with a significantly higher isolation rate of 55% (33/60) during the winter compared to 20% (12/60) in the summer (*p* = 0.00016).

### Phenotypic and genotypic data on antimicrobial resistance

**Fig. 1:**
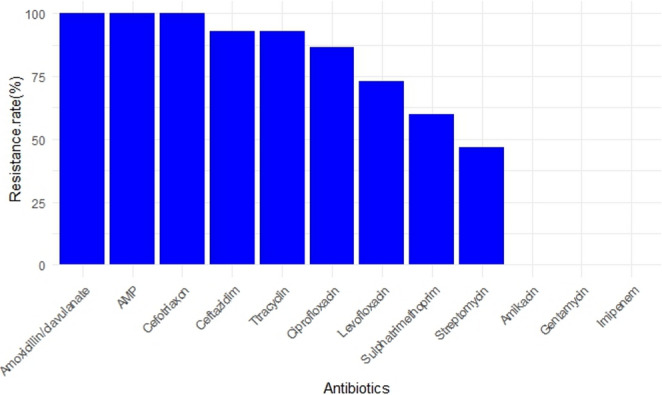
Percentage resistance of *E. coli* isolates to various antibiotics

**Fig. 2:**
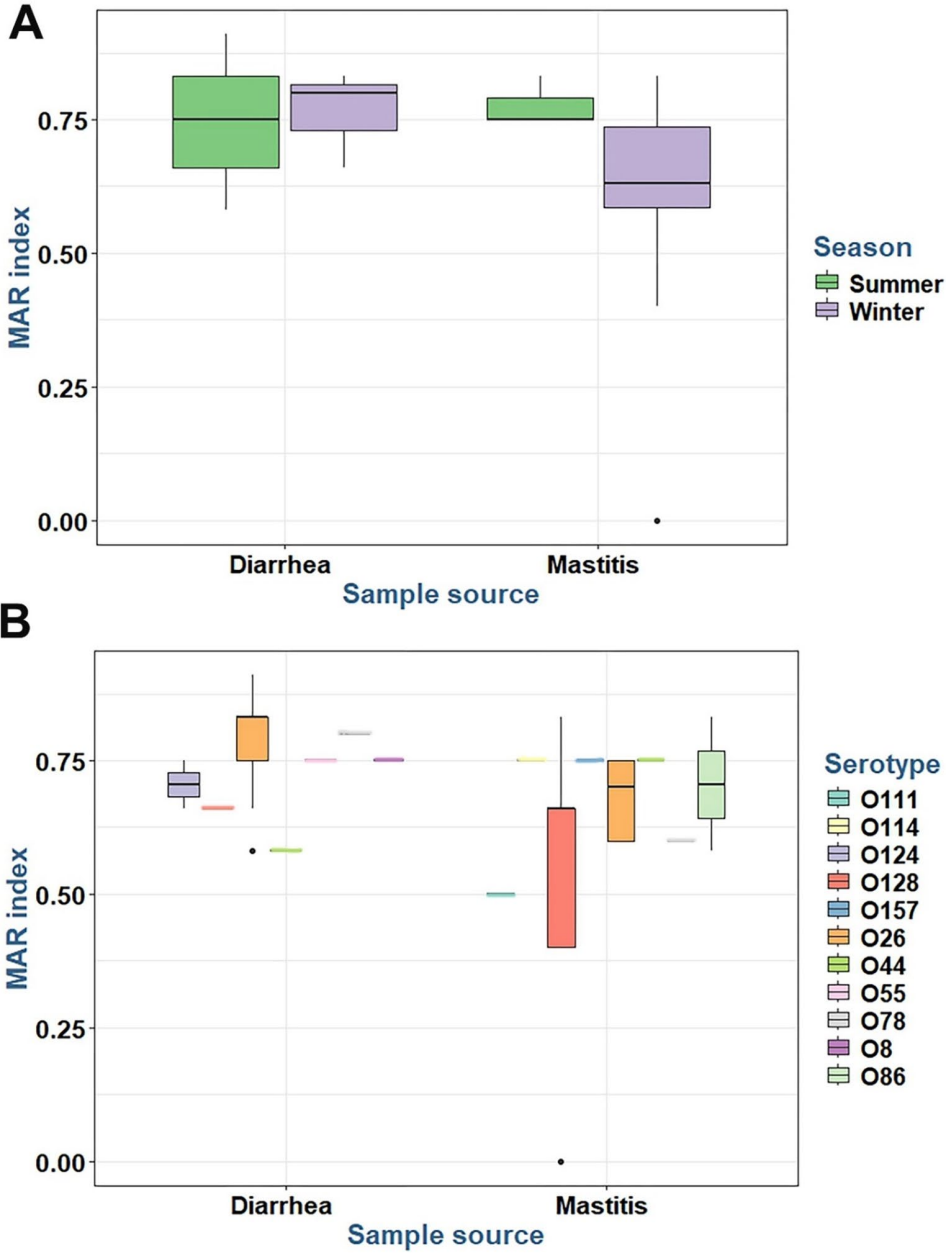
The multiple antibiotic resistance (MAR) index of the tested *E. coli* isolates belonging to various sources, seasons (**A**), and serotypes (**B**)

**Fig. 3:**
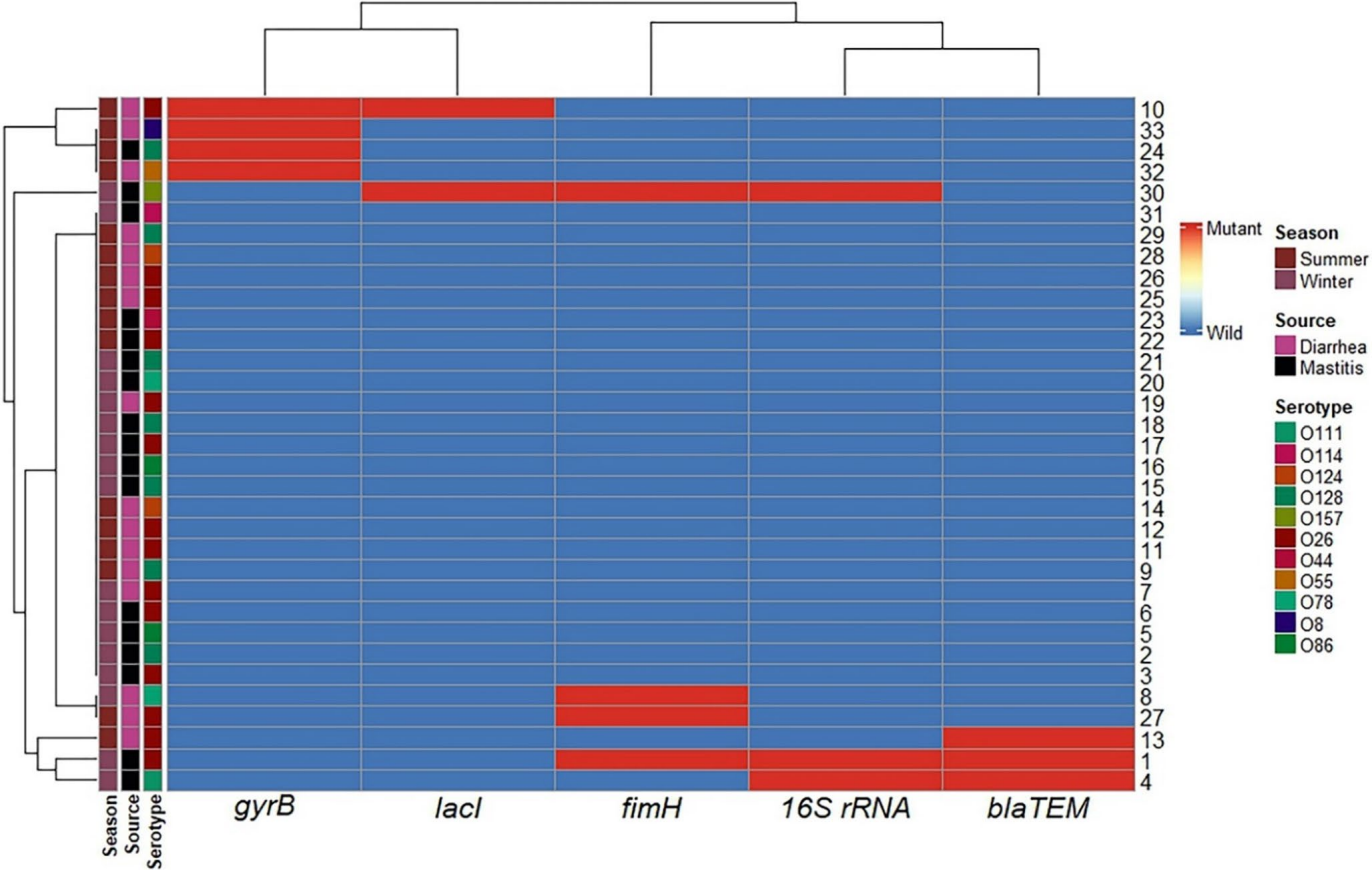
Hierarchical clustering heatmap showing the overall distribution of the investigated *E. coli* isolates based on the presence of mutation in the investigated genes via SSCP-PCR assay. Different seasons, sample sources, and serotypes are color-coded on the right of the heatmap

All *E. coli* isolates (100%) demonstrated complete susceptibility to the aminoglycoside antibiotics (Amikacin and Gentamicin) and Imipenem. In contrast, widespread resistance was observed against several other drugs, including 100% resistance to Ampicillin, Amoxicillin/clavulanate, and Ceftazidime. This phenotypic resistance was consistent with a high prevalence of multidrug resistance (86.6% of isolates). A total of nine distinct antibiotic resistance patterns were identified (Fig. 1), and the calculated Multiple Antibiotic Resistance (MAR) index ranged from 0.33 to 0.91 (Figures S2-S3). We observed a significant seasonal influence on resistance, with a higher mean MAR index in summer isolates (*p* = 0.0095), and a significant difference between isolates from calf diarrhea and mastitis cases (*p* = 0.0358) as shown in Fig. 2 and Supplementary Tables S1-S3. This widespread phenotypic resistance was genotypically corroborated by the frequent detection of mutations in the *blaTEM* and *gyrB* genes (Fig. 3).

### Overall mutation profiles via SSCP-PCR screening

**Fig. 4:**
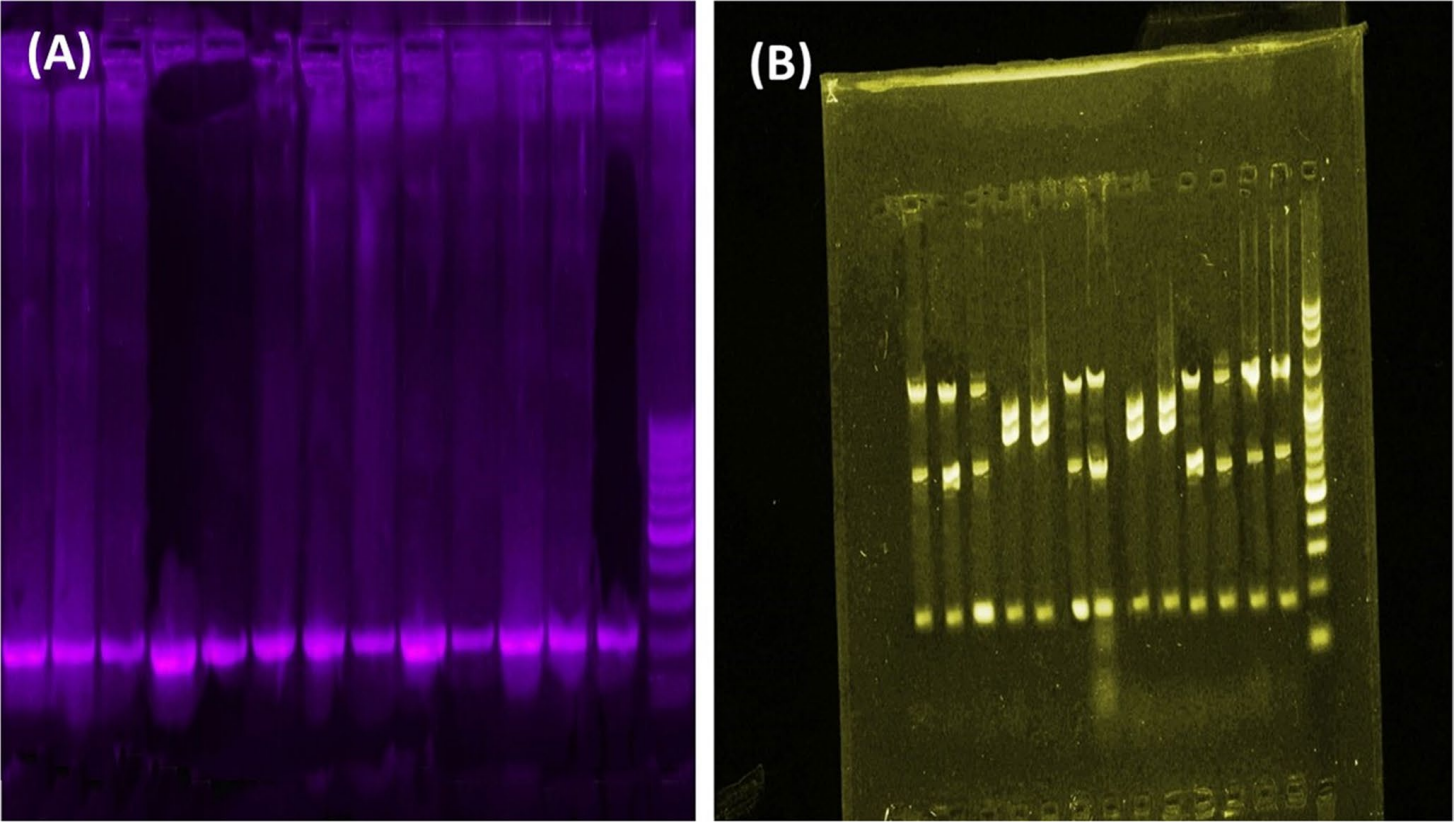
Conventional PCR amplification and single-strand conformation polymorphism (SSCP) analysis of the 229 bp *gyrB* gene from *E. coli* isolates. Panel (**A**) demonstrates the successful amplification of the 229 bp *gyrB* gene fragment from various *E. coli* isolates via conventional PCR, as shown by the presence of a single, uniform band in each lane. Panel (**B**) displays the SSCP profiles of these *gyrB* amplicons, revealing distinct banding patterns. These patterns indicate different single-stranded DNA conformations, suggesting underlying nucleotide variations (polymorphisms or mutations) within the *gyrB* gene among the analyzed *E. coli* isolates. Different banding patterns correspond to isolates with potentially different *gyrB* sequences compared to a presumed wild-type strain or among each other

A total of 33 *E. coli* isolates were screened for mutations across five target genes using the SSCP-PCR assay. Thirteen isolates (13/33) were found to harbor at least one mutation. The overall distribution of these mutations is visually represented in a hierarchical clustering heatmap (Fig. 3), showing distinct genotypic profiles. Analysis revealed that genetic variations were most frequently observed in the *blaTEM*, *gyrB*, and *fimH* genes, which correspond to resistance and virulence, while mutations in the *lacI* and highly conserved* 16S rRNA* genes were less common. The SSCP gel images (Fig. 4) provide a visual representation of the band shifts that indicate the presence of these mutations. This initial screening successfully identified the isolates with genetic variations for subsequent sequencing.

### Molecular characterization of gene mutations via sanger sequencing

Sanger sequencing was performed on all SSCP-positive isolates to precisely characterize the underlying genetic variations. This approach provided atomic-level insights into the molecular changes driving the adaptive traits of the *E. coli* isolates, with all generated partial gene sequences submitted to the NCBI GenBank (Accession numbers provided in Supplementary Table S4).

**Fig. 5:**
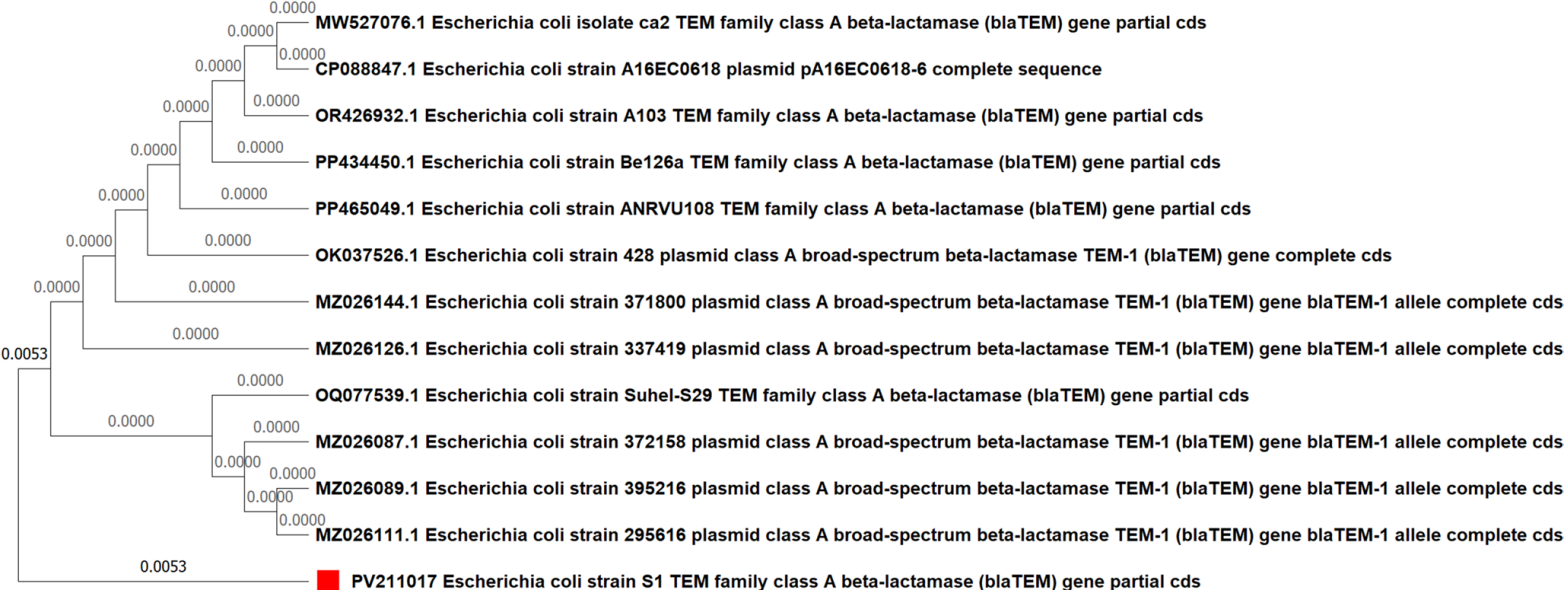
Phylogenetic tree of the examined *Escherichia coli* isolate strain S1based on the *blaTEM* gene partial sequence generated via the neighbor-joining technique. ▪ our examined *E. coli* isolate

**Fig. 6:**
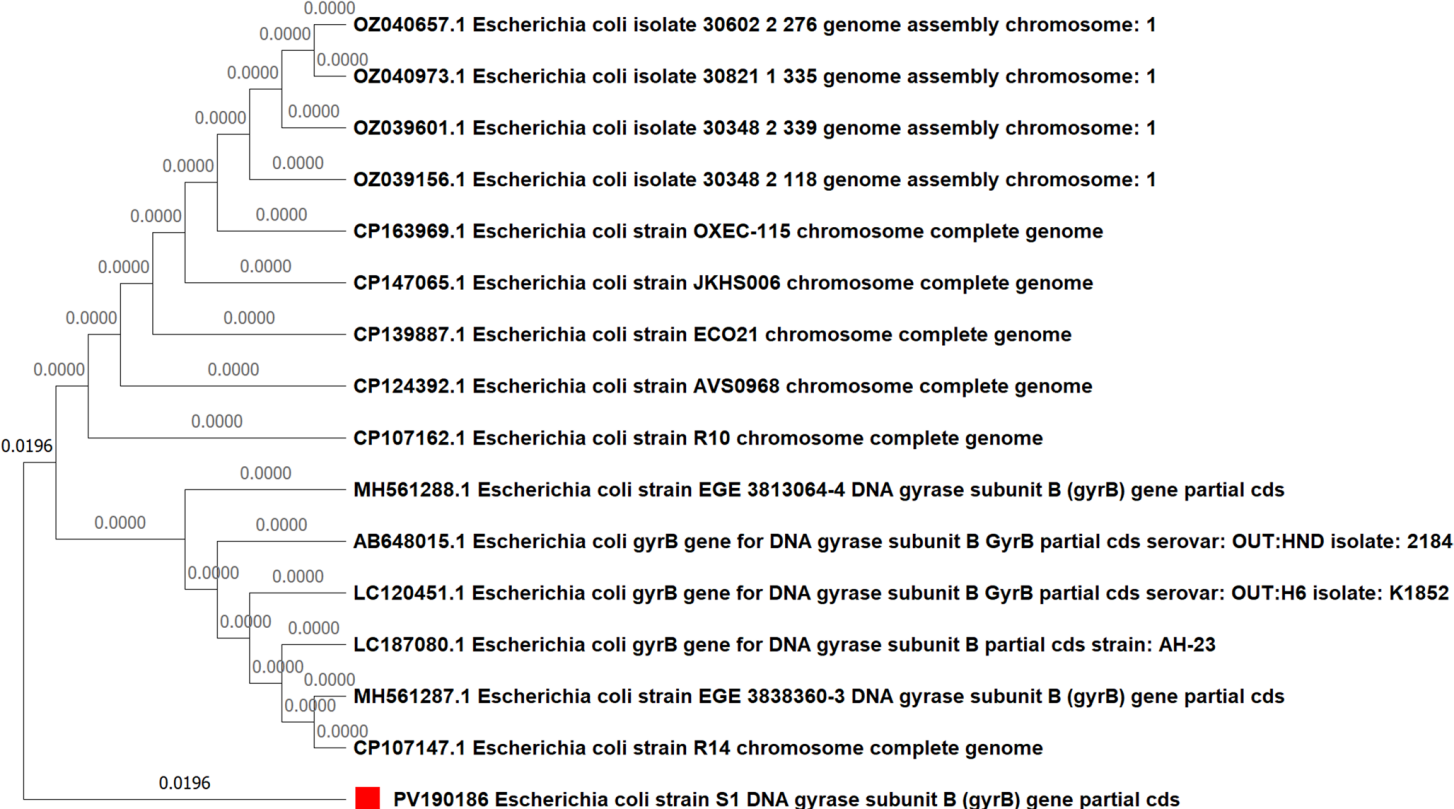
Phylogenetic tree of the examined *Escherichia coli* isolate strain S1 based on the *gyrB* gene partial sequence generated via the neighbor-joining technique. ▪ our examined *E. coli* isolate

Sequencing of the *blaTEM* gene, for example, confirmed the presence of distinct missense mutations in three isolates, providing the direct genetic basis for the observed widespread β-lactam resistance. Each isolate displayed a unique mutational profile, such as the Arg → Trp substitution in one strain, highlighting the dynamic nature of resistance development (Fig. 5). Similarly, sequencing of the *gyrB* gene in four isolates confirmed the presence of distinct missense mutations, including Leu → Glu and Asp → Glu substitutions (Fig. 6). These specific amino acid changes, located within the Quinolone Resistance Determining Regions (QRDRs), are well-established mechanisms for conferring fluoroquinolone resistance. The genetic heterogeneity observed across these isolates suggests multiple, independent evolutionary events rather than the clonal dissemination of a single variant.

**Fig. 7:**
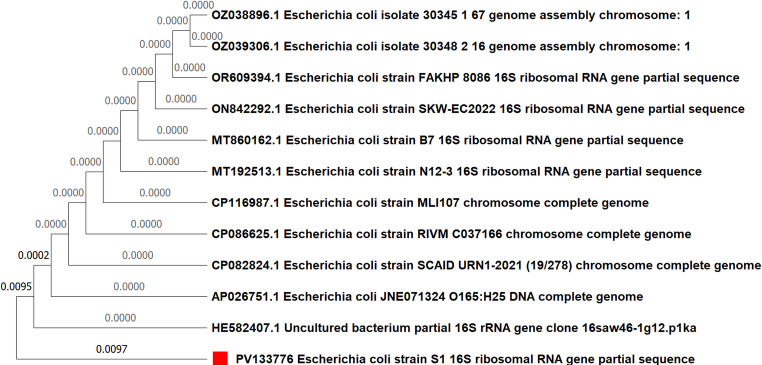
Phylogenetic tree of the examined *Escherichia coli* isolate strain S1 (ID; 1) based on the *16S rRNA* gene partial sequence generated via the neighbor-joining technique. ▪ our examined *E. coli* isolate

Beyond resistance genes, sequencing of the *fimH* virulence gene revealed diverse missense mutations across four isolates. These changes can significantly impact the structure of the FimH adhesin, directly affecting bacterial colonization capabilities. Additionally, our analysis identified notable mutations in the metabolic gene *lacI* and the highly conserved 16*S rRNA* gene. For instance, a nonsense mutation leading to a premature stop codon was detected in the *16 S rRNA* gene of one isolate, a rare event highlighting profound genetic adaptability (Fig. 7). The specific details of all confirmed mutations, including nucleotide and amino acid changes, are presented in Supplementary Table S4 and Supplementary Figures S3-S60. The co-occurrence of these mutations, particularly the strong positive correlation between the rare *16S rRNA* mutation and mutations in *blaTEM*, *fimH*, and *lacI* (as shown in Fig. 3), provides compelling evidence of a co-evolutionary process.

### Correlation between MAR index and gene mutations

**Fig. 8:**
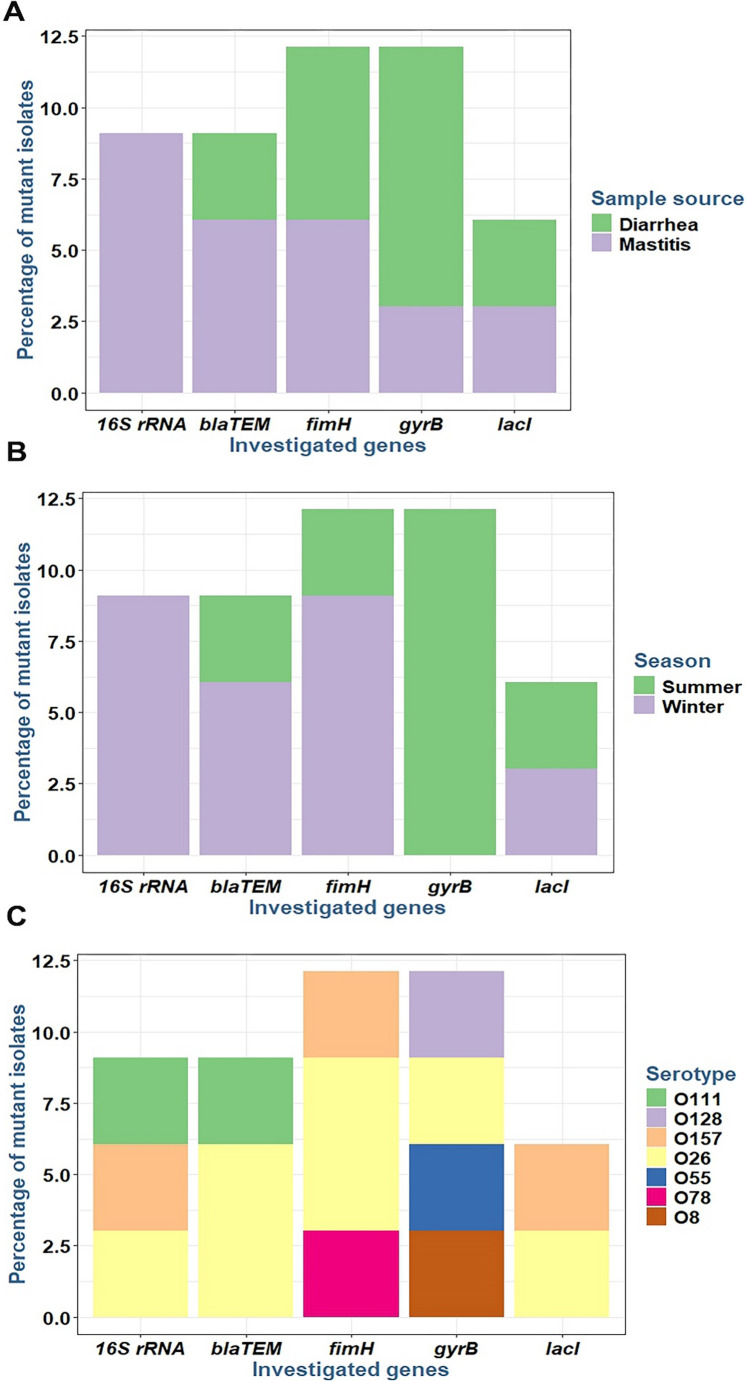
Prevalence of mutation among investigated genes of *E. coli* isolates obtained from various sources (**A**), seasons (**B**), and serotypes (**C**)

**Fig. 9:**
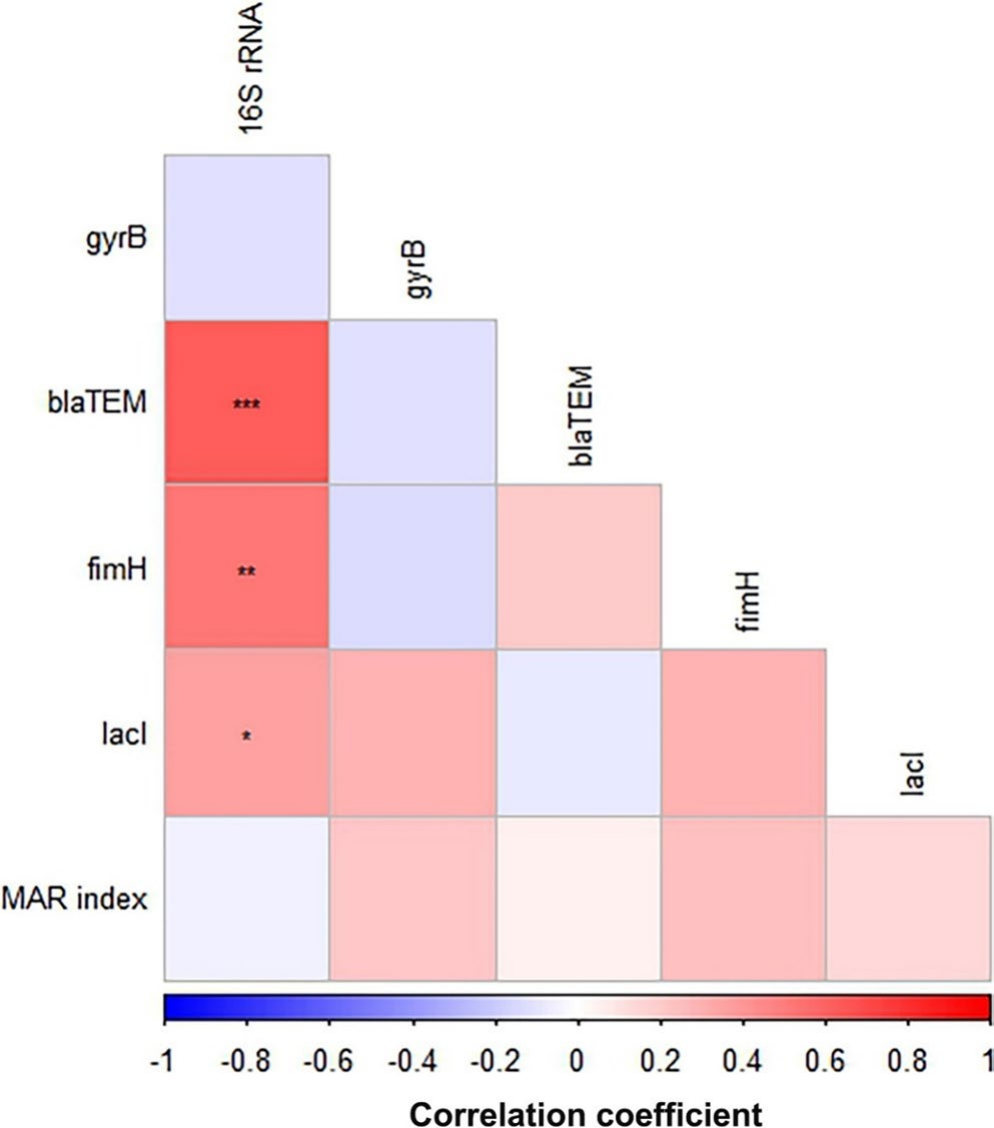
Pairwise correlation (*r*) between the MAR index, and the existence of mutations in the investigated genes of *E. coli* isolates. The scale below the figure refers to the correlation coefficient (*r*). The more intense the color, the more the stronger the positive or negative correlation. Stars refer to the significant correlation; *p < 0.05, **p < 0.01, ***p < 0.001

The prevalence of mutations within the investigated genes did not significantly differ ( p>0.05 ) when isolates were grouped by source, season of isolation, or serotype (Figure 8). Figure 9 presents the pairwise correlation coefficients (r) between the Multiple Antibiotic Resistance (MAR) index and the existence of mutations (as detected by SSCP-PCR) in the five investigated *E. coli* genes (*16 S rRNA*,* gyrB*,* blaTEM*,* fimH*, and *lacI*). The color intensity and scale indicate the strength and direction of the correlation, with red representing positive and blue representing negative correlations. Statistical significance is denoted by asterisks (*p < 0.05, **p < 0.01, ***p < 0.001).

As shown in Fig. 9, several significant positive correlations were identified between the presence of mutations in the* 16S rRNA* gene and mutations in other genes:

- A highly significant positive correlation was observed between the existence of mutation in *16S rRNA* and mutations in *blaTEM* (*r* = 0.63, *p* < 0.0001).
- A significant positive correlation was also found between *16S rRNA* mutation and mutations in *fimH* (*r* = 0.53, *p* = 0.0015).
- A moderately significant positive correlation was noted between *16S rRNA* mutation and mutations in *lacI* (*r* = 0.38, *p* = 0.03). Other pairwise correlations between gene mutations generally showed weaker or non-significant positive relationships (e.g., between *blaTEM* and *fimH*,* gyrB* and *fimH*). The MAR index, representing the overall antibiotic resistance profile, did not show any strong or statistically significant correlation with the existence of mutations in any of the individual investigated genes, as indicated by the generally light blue/white squares in the bottom row/leftmost column.

## Discussion

Our cross-sectional study in Garbia Governorate, Egypt, represents a crucial and novel step in understanding the complex epidemiology, serotype diversity, and antimicrobial resistance (AMR) patterns of *E. coli* circulating within bovine populations. By taking a comprehensive “Farm to Forefront” approach, we uniquely connect real-world disease dynamics at the farm level with intricate molecular adaptations, particularly highlighting how climate-driven pressures shape the genetic landscape of virulent *E. coli*. The application of the Single-Strand Conformation Polymorphism Polymerase Chain Reaction (SSCP-PCR) assay, using our newly designed primers, was pivotal in unraveling these genetic nuances, providing a high-throughput and cost-effective method to screen for genetic variations.

Our overall *E. coli* isolation rate of 32.4% (94/290) provides an important baseline for local prevalence and underscores the widespread circulation of this pathogen on dairy farms in the region. While our data showed no statistically significant seasonal difference for clinical mastitis, this finding is critically important as it points to a persistent, year-round problem driven by fundamental management and hygiene challenges. These challenges are exacerbated by fluctuating climatic conditions. Warmer, drier conditions characteristic of Egyptian summers lead to increased environmental dust and create an ideal milieu for bacterial survival and proliferation in bedding and manure, thereby amplifying mastitis risk and placing greater strain on farm biosecurity practices (Ghali-Mohammed et al. 2023; Xu et al. 2023).

In stark contrast, calf diarrhea exhibited a highly significant seasonal variation (*p* = 0.00016), peaking dramatically in winter. This pronounced winter incidence is biologically plausible, as colder, wetter environmental stressors directly compromise calf immunity through thermal stress and facilitate pathogen transmission through damp bedding and increased animal density in winter housing. This observation highlights that the clinical incidence of disease is significantly influenced by host-specific factors and immune system vulnerability to cold stress. This aligns with other regional studies, strongly implicating these environmental factors as direct drivers of disease (El-Nady et al. 2023; Fouad et al. 2024). The diverse *E. coli* serotypes identified, notably the prevalence of O26 and O128 across both calf diarrhea and mastitis isolates, strongly suggests shared epidemiological links and active inter-animal transmission. These versatile serotypes appear to be well-adapted colonizers of multiple host niches on the farm. Of particular critical concern is the isolation of *E. coli* O157 from a mastitis sample, a finding that immediately elevates public health risks and establishes a clear and present danger of farm-to-table transmission, given its potential for causing severe human illnesses like hemorrhagic colitis and hemolytic uremic syndrome (HUS).

Our investigation further reveals a deeply concerning landscape of antimicrobial resistance (AMR) in bovine *E. coli*. While Amikacin, Gentamicin, and Imipenem maintain their effectiveness, the widespread resistance to β-lactam antibiotics such as Ampicillin, Amoxicillin/clavulanate, and Ceftazidime signifies a high prevalence of β-lactamase-producing *E. coli* strains. This pattern, coupled with a high observed multidrug resistance (MDR) rate, aligns with alarming trends documented regionally and globally (Rodríguez-Baño et al. 2018; Tomeh et al. 2024; Gajic et al. 2025).

While we observed higher resistance rates in summer isolates, we do not attribute this directly to temperature or climate change alone. Our findings provide local evidence of a well-documented phenomenon. We hypothesize that the higher ambient temperatures during the summer months in Garbia Governorate, Egypt (as supported by local meteorological data), act as a powerful environmental pressure that contributes to higher disease incidence. This leads to a predictable escalation in antimicrobial use, which is the primary selective force driving the selection and proliferation of resistant bacterial strains. Crucially, we also hypothesize that the direct physiological stress of extreme heat on the bacterial cells themselves accelerates microbial metabolism and mutation rates, providing a powerful selective pressure for the co-evolution of resistance and virulence genes. We also recognize that other potential mechanisms, such as increased horizontal gene transfer (HGT) at higher temperatures and the mechanical spread of bacteria via increased insect activity in warmer months, likely contribute to the observed increase in AMR (Zhang and Cheng 2022).

The divergence in MAR index between isolates from different infection sites (mastitis vs. diarrheal) also provides a nuanced understanding of on-farm resistance profiles. The consistently higher resistance observed in diarrheic calf isolates is likely due to the more intense and systemic selective pressures of oral antibiotic use, which contrasts with the more localized intramammary treatments for mastitis. This underscores how specific infection niches and their associated, often distinct, treatment regimens contribute to varied resistance landscapes.

To unravel the intricate molecular underpinnings of these phenotypes, we employed a targeted molecular approach. The SSCP-PCR assay, as a robust screening tool, enabled us to identify isolates with genetic variations. The prevalence of mutations in *blaTEM* directly corroborates the observed widespread phenotypic resistance to β-lactam antibiotics (Fashae et al. 2021). Similarly, the detection of mutations in *gyrB*, a primary target for fluoroquinolone antibiotics, directly links our genotypic findings to observed phenotypic resistance, and the diversity of missense mutations suggests multiple, independent evolutionary events rather than the clonal dissemination of a single variant (Malik et al. 2012; Shariati et al. 2022). Beyond resistance, mutations in *fimH*, a pivotal virulence factor, suggest adaptive changes in *E. coli*’s capacity for host tissue colonization or immune evasion (Hojati et al. 2015; Yoshida et al. 2022).

our results provide compelling support through the co-occurrence of mutations in multiple genes. A particularly seminal and critical finding evident from our correlation heatmap (Fig. 9) is the strong and highly significant positive correlation between mutations in the *16S rRNA* gene and mutations in *blaTEM*, *fimH*, and *lacI*. Previous studies recorded that random mutations are a common occurrence in *E. coli* (Algammal et al. 2020; Brunauer et al. 2021). However, the significance of our findings lies not in the mere presence of these mutations, but in their compelling co-occurrence. A mutation in the *16S rRNA* gene is a relatively rare event due to its high conservation. The co-occurrence of this rare mutation with adaptive mutations in resistance and virulence genes provides strong support for the hypothesis of clonal expansion (Jansen et al. 2013; García-Meniño et al. 2024). This co-occurrence is highly improbable to be a random event. Instead, it suggests that a specific, successful clonal lineage, having acquired a rare mutation in its core machinery, was able to expand and subsequently accumulate additional beneficial mutations in genes for antibiotic resistance, virulence, and metabolism. This suite of co-inherited mutations represents a highly successful, integrated adaptive strategy that was selected for by the on-farm environmental pressures. Our findings therefore support the premise by showing that a single, rare mutational event (in the *16S rRNA* gene) was propagated through a successful lineage, which then became a repository for other advantageous mutations. This demonstrates that resistance genes are not evolving in isolation but are intricately intertwined with other essential bacterial functions, impacting not only drug resistance but also host colonization and nutrient utilization (Yang et al. 2016).

## Conclusion

This comprehensive study provides groundbreaking insights into the intricate and dynamic landscape of antimicrobial resistance in bovine *E. coli*, effectively bridging crucial farm-level epidemiological observations with advanced molecular genetic discoveries. We have unequivocally established widespread phenotypic resistance and, importantly, unveiled a critical, previously underappreciated, climate-driven nexus where climate-driven nexus where seasonal pressures influence both disease incidence (through host stress) and the genetic evolution of pathogens (through microbial stress). Our findings reveal that while the clinical manifestation of disease peaks in winter, the most significant genetic adaptations in AMR and virulence occur in summer isolates, demonstrating a direct link between environmental heat stress and genetic co-evolution. A particularly seminal finding is the direct molecular evidence supporting clonal expansion as a key mechanism for the co-inheritance of resistance and virulence genes.

While our SSCP-PCR assay provided a vital snapshot of point mutations, it is important to acknowledge that it does not capture all resistance mechanisms, such as the rapid acquisition of plasmid-borne genes. This highlights the multifaceted nature of AMR and underscores the urgent necessity for broader, integrated molecular investigations. We recommend that future studies, ideally through whole-genome sequencing (WGS), be conducted to fully characterize the complete resistome, confirm clonal relationships, and provide a deeper understanding of the genetic plasticity of these pathogens. Ultimately, our findings are indispensable for deciphering the dynamic processes of AMR and virulence dissemination in a climate-influenced context. This work emphasizes the urgent need for integrated, climate-informed AMR surveillance and control strategies in livestock, which are paramount for safeguarding both animal and human health within the interconnected “One Health” paradigm.

## Supplementary Information

Below is the link to the electronic supplementary material.

Supplementary Material 1 (DOCX 7.41 MB)

## Data Availability

All data generated or analyzed during this study are included in this research article and its supplementary information files.

## Abbreviations

AMR: Antimicrobial resistance
MDR: Multi-drug resistance
SSCP-PCR: Single-Strand Conformation Polymorphism Polymerase Chain Reaction
