# Supplementary material for: Climate-driven co-evolution of antimicrobial resistance and virulence in Escherichia coli on dairy farms: unraveling adaptive genetic signatures with novel SSCP-PCR
Source: World J Microbiol Biotechnol. 2025 Oct 28;41(11):419. doi: 10.1007/s11274-025-04616-z (PMC12568838; doi:10.1007/s11274-025-04616-z)
Supplement: Supplementary file 1 — Supplementary Material 1 (DOCX 7.41 MB) [file 11274_2025_4616_MOESM1_ESM.docx]

**Supplementary Table S1.** Prevalence of mutation among investigated genes of *E. coli* isolates obtained from various sources

| Genes | No. of mutant isolates (%) | | *p*-value | Total no. of mutant isolates (%) (n= 33) |
| --- | --- | --- | --- | --- |
|  | Mastitis (n= 17) | Diarrhea (n= 16) |  |  |
| *16S rRNA* | 3 (17.6) | 0 | 0.227 | 3 (9.09) |
| *gyrB* | 1 (5.88) | 3 (18.75) | 0.335 | 4 (12.12) |
| *blaTEM* | 2 (11.76) | 1 (6.25) | 1 | 3 (9.09) |
| *fimH* | 2 (11.76) | 2 (12.5) | 1 | 4 (12.12) |
| *lacI* | 1 (5.88) | 1 (6.25) | 1 | 2 (6.06) |

**Supplementary Table S2.** Prevalence of mutation among investigated genes of *E. coli* isolates obtained from various seasons

| Genes | No. of mutant isolates (%) | | *p*-value | Total no. of mutant isolates (%) (n= 33) |
| --- | --- | --- | --- | --- |
|  | Summer (n= 16) | Winter (n= 17) |  |  |
| *16S rRNA* | 0 | 3 (17.6) | 0.227 | 3 (9.09) |
| *gyrB* | 4 (25) | 0 | 0.044* | 4 (12.12) |
| *blaTEM* | 1 (6.25) | 2 (11.76) | 1 | 3 (9.09) |
| *fimH* | 1 (6.25) | 3 (17.6) | 0.601 | 4 (12.12) |
| *lacI* | 1 (6.25) | 1 (5.88) | 1 | 2 (6.06) |

**p*< 0.05.

**Supplementary Table S3.** Prevalence of mutation among investigated genes of *E. coli* isolates obtained from various serotypes

| Genes | No. of mutant isolates (%) | | | | | | | *p*-value | Total no. of mutant isolates (%) (n= 33) |
| --- | --- | --- | --- | --- | --- | --- | --- | --- | --- |
|  | O26 (n= 14) | O111 (n= 1) | O157 (n=1) | O128 (n=7) | O55 (n=1) | O8 (n=1) | O78 (n=2) |  |  |
| *16S rRNA* | 1 (7.14) | 1 (100) | 1 (100) | 0 | 0 | 0 | 0 | 0.095 | 3 (9.09) |
| *gyrB* | 1 (7.14) | 0 | 0 | 1 (14.29) | 1 (100) | 1 (100) | 0 | 0.182 | 4 (12.12) |
| *blaTEM* | 2 (14.29) | 1 (100) | 0 | 0 | 0 | 0 | 0 | 0.378 | 3 (9.09) |
| *fimH* | 2 (14.29) | 0 | 1 (100) | 0 | 0 | 0 | 1 (50) | 0.323 | 4 (12.12) |
| *lacI* | 1 (7.14) | 0 | 1 (100) | 0 | 0 | 0 | 0 | 0.341 | 2 (6.06) |

**Supplementary Table S4.** Molecular Characterization of Mutations in *E. coli* Genes by Sanger Sequencing

| Gene | Strain ID | Accession Number | Mutation(s) |
| --- | --- | --- | --- |
| 16S rRNA | S1 | PV133776 | Nucleotide substitution at position 31 (silent mutation) and position 42 (missense mutation at amino acid 14: Met → Lys) |
|  | S2 | PV133781 | Nucleotide substitutions at positions 17 (missense mutation at amino acid 7: Asp → Val) and 104 (nonsense mutation at amino acid 35: Leu → premature stop codon) |
| gyrB | S1 | PV190186 | Nucleotide substitutions at positions 48-50 (missense mutation at amino acid 17: Leu → Glu) and 117 (missense mutation at amino acid 39: Asp → Glu) |
|  | S2 | PV190187 | Nucleotide substitutions at positions 24 (missense mutation at amino acid 8: Arg → Pro) and 114-116 (missense mutation at amino acid 38: Val → Glu and amino acid 39: Asn → Tyr) |
|  | S3 | PV190188 | Nucleotide substitutions at positions 107-109 (missense mutation at amino acid 36: Leu → Glu) and 177 (missense mutation at amino acid 59: Asn → Tyr) |
|  | S4 | PV190189 | Nucleotide substitutions at positions 57 (missense mutation at amino acid 19: Val → Leu), 127-128 (missense mutation at amino acid 42: Lys → Ile), and 197 (missense mutation at amino acid 65: Asp → Glu) |
| blaTEM | S1 | PV211017 | Nucleotide substitution at position 205 (missense mutation at amino acid 69: Arg → Trp) |
|  | S2 | PV197669 | Nucleotide substitutions at positions 27-29 (missense mutation at amino acid 10: Arg → Ala), 97 (missense mutation at amino acid 33: Val → Leu), and 168 (silent mutation) |
|  | S3 | PV197670 | Nucleotide substitutions at positions 103 (missense mutation at amino acid 35: Gly → Arg), 172-173 (missense mutation at amino acid 58: Arg → Ala), and 143 (missense mutation at amino acid 81: Phe → Leu) |
| fimH | S1 | PV209838 | Nucleotide substitutions at positions 26-28 (missense mutations at amino acid 9: Leu → Gln and amino acid 10: Ile → Phe) and 96-98 (missense mutation at amino acid 33: Leu → Asp) |
|  | S2 | PV209839 | Nucleotide substitutions at positions 33 (missense mutation at amino acid 11: Gln → Glu), 103-104 (missense mutation at amino acid 34: Ala → Gly), and 173 (missense mutation at amino acid 57: Lys → Asn) |
|  | S3 | PV209840 | Nucleotide substitutions at positions 83 (missense mutation at amino acid 28: Cys → Ser) and 96 (missense mutation at amino acid 32: Leu → Gln) |
|  | S4 | PV209841 | Nucleotide substitution at position 94 (missense mutation at amino acid 32: Pro → Ala) |
| lacI | S1 | PV231894 | Nucleotide substitutions at positions 35 (missense mutation at amino acid 11: His → Gln) and 173 (missense mutation at amino acid 57: Gln → His) |
|  | S2 | PV209837 | Nucleotide substitutions at positions 37 (silent mutation) and 174-175 (missense mutation at amino acid 58: Ile → Asn) |


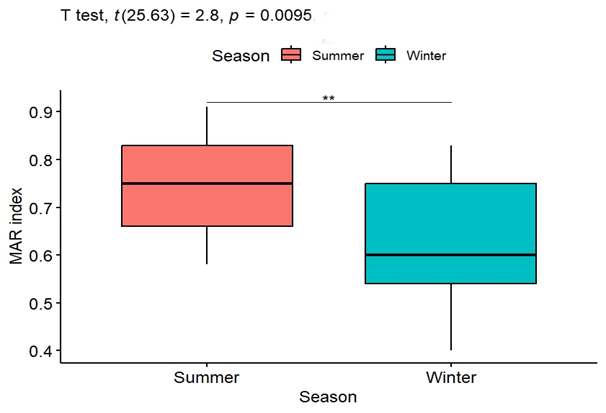


**Figure S1.** Seasonal Variation in Multiple Antibiotic Resistance (MAR) Index of *E. coli* Isolates, showing significantly higher resistance in Summer.


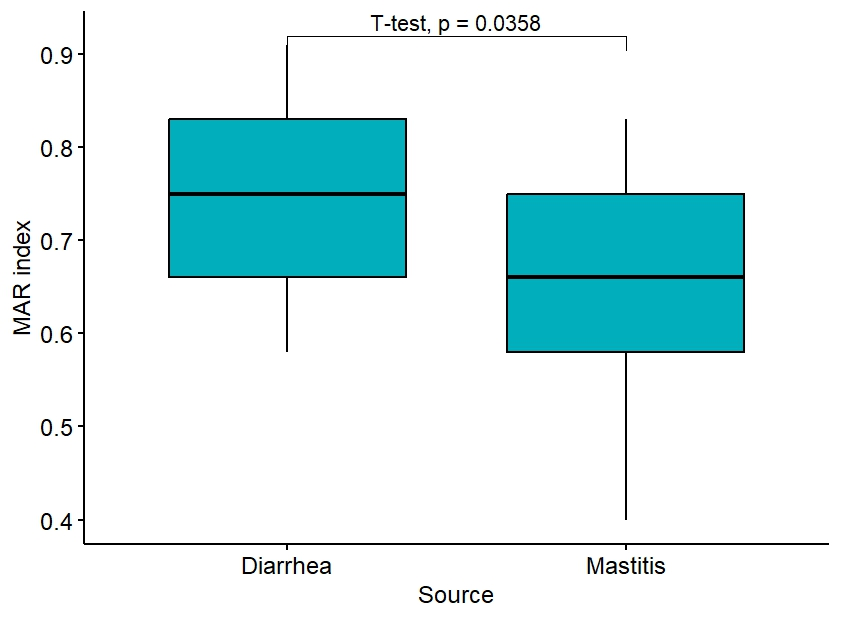


**Figure S2.** Comparison of MAR Index of *E. coli* Isolates from Diarrhea and Mastitis Samples.


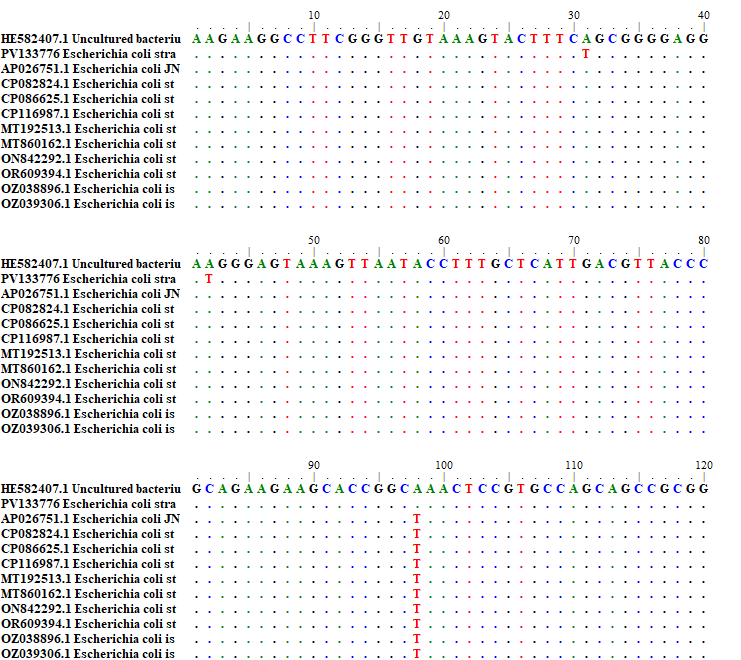


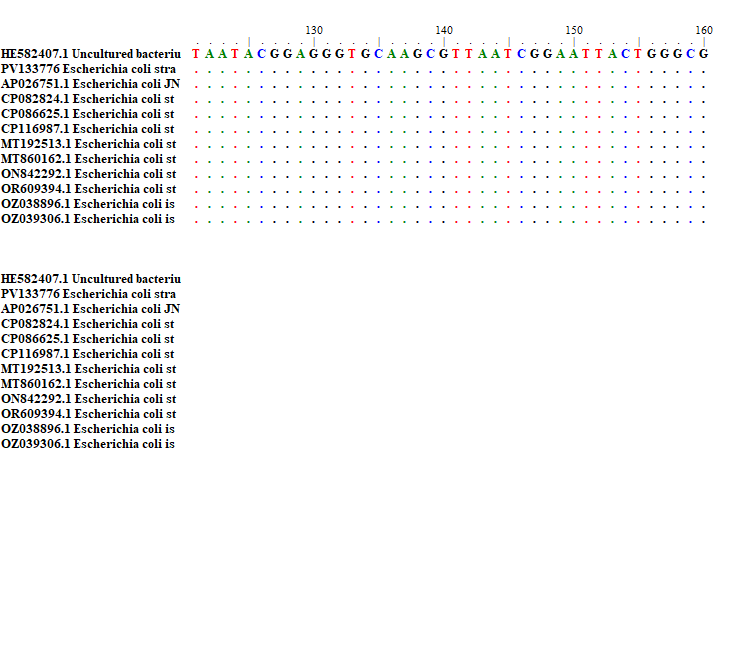


**Figure S3.** Nucleotide sequence alignments of the *16S rRNA* gene of the examined *Escherichia coli* isolate strain S1 with PV133776 accession number. The sequences are indicated by their accession numbers.


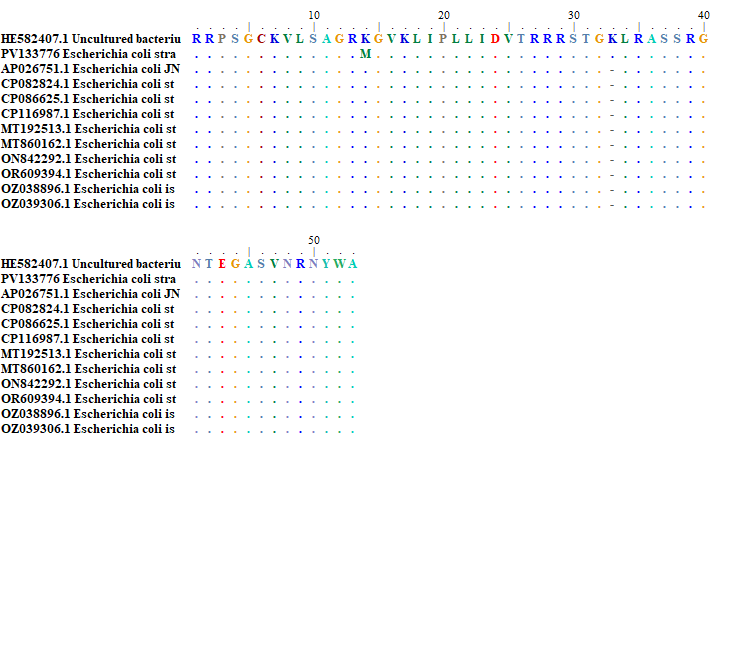


**Figure S4.** Amino acid sequence alignments of the *16S rRNA* gene of the examined *Escherichia coli* isolate strain S1 with PV133776 accession number. The sequences are indicated by their accession numbers.


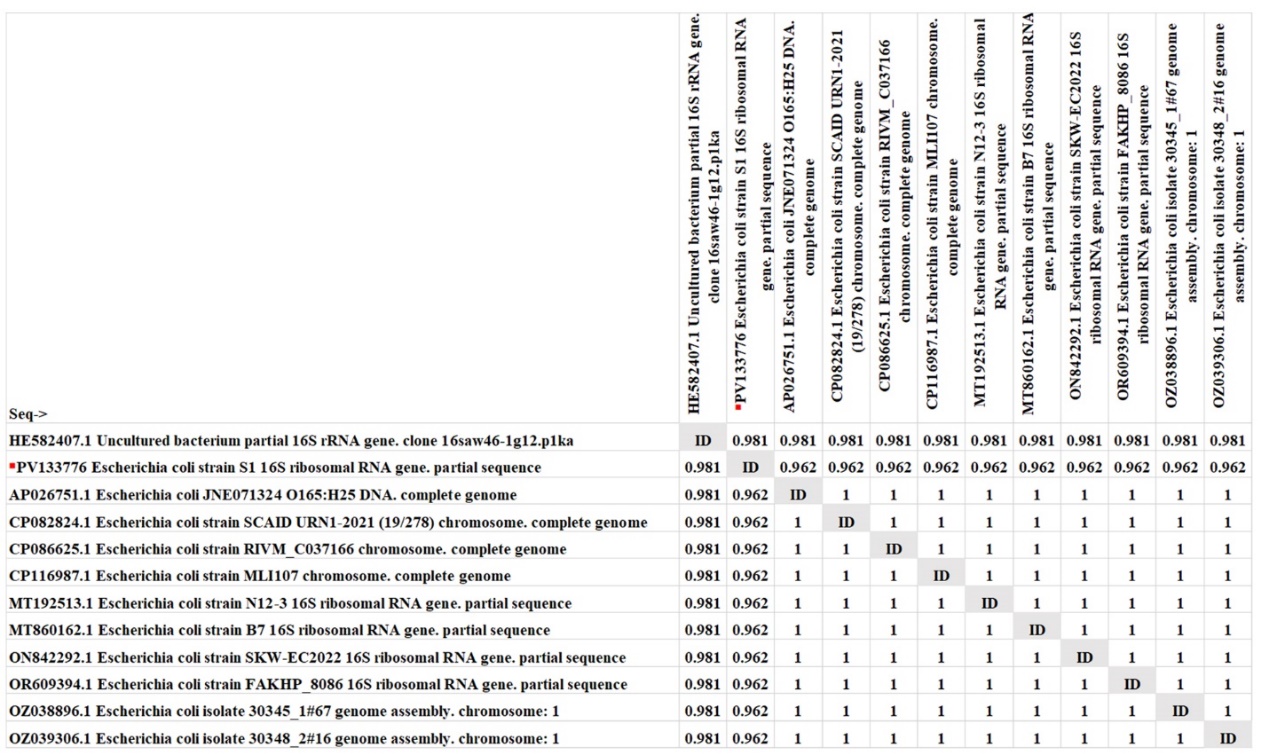


**Figure S5.** Amino acid identity percentages between the *16S rRNA* genes of the examined *Escherichia coli* isolate strain S1, and other *E. coli* isolates on the GenBank. ID: identity, ▪ Our examined *E. coli* isolate.


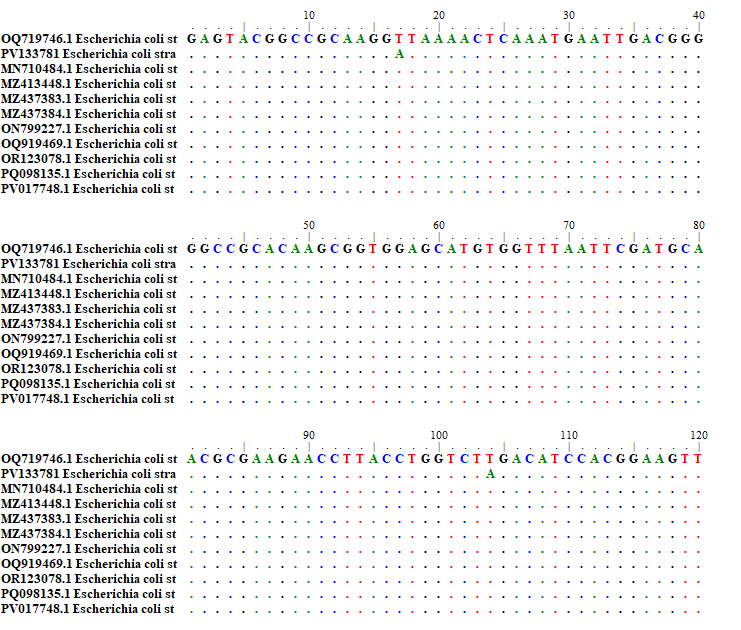


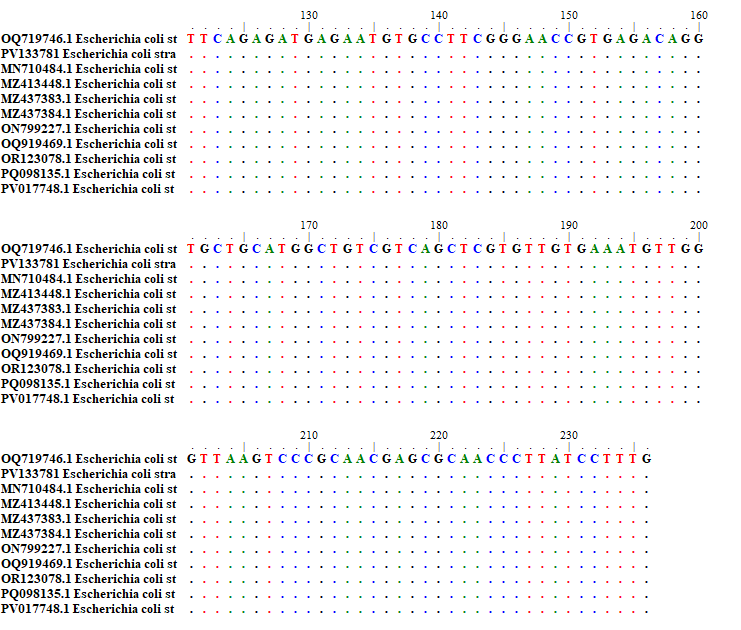


**Figure S6.** Nucleotide sequence alignments of the *16S rRNA* gene of the examined *Escherichia coli* isolate strain S2 with PV133781 accession number. The sequences are indicated by their accession numbers.


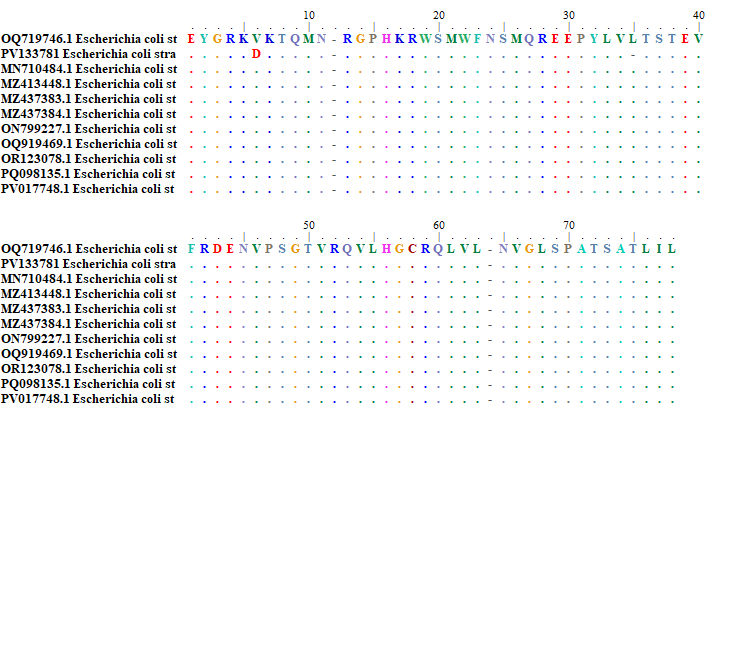


**Figure S7.** Amino acid sequence alignments of the *16S rRNA* gene of the examined *Escherichia coli* isolate strain S2 with PV133781 accession number. The sequences are indicated by their accession numbers.


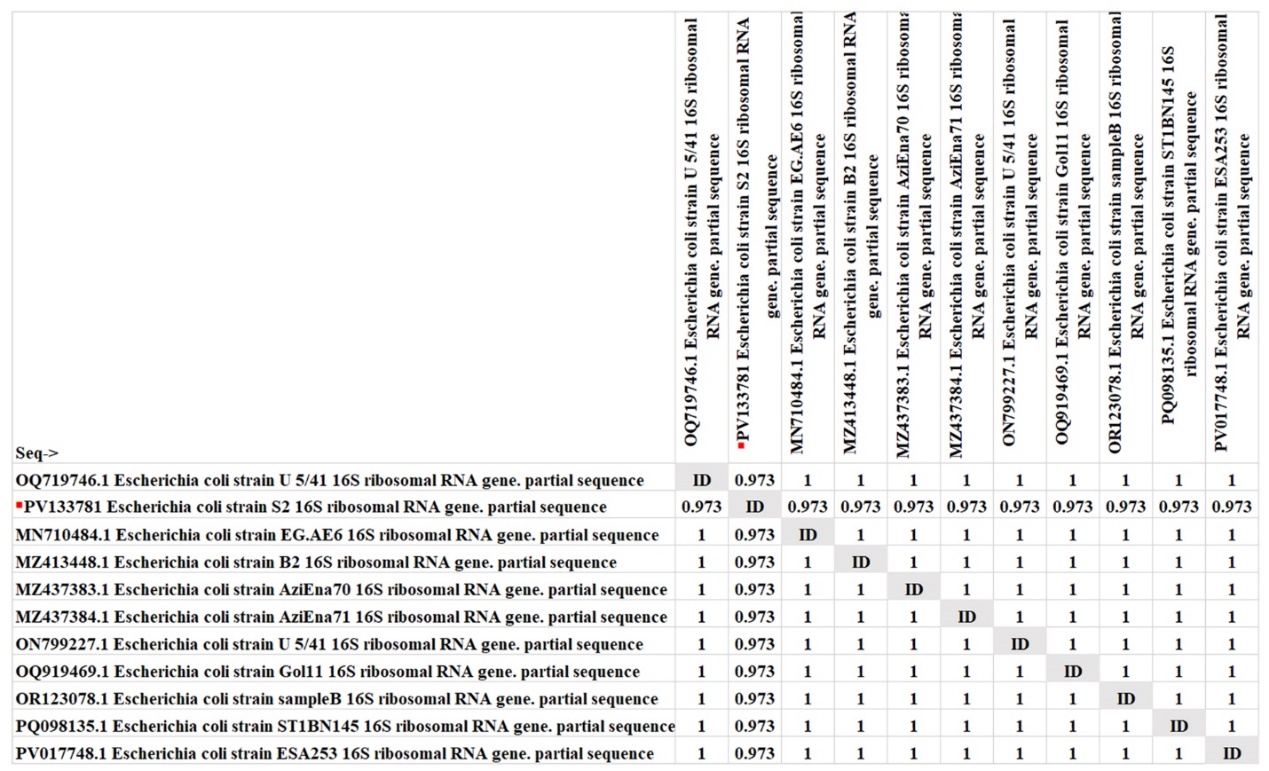


**Figure S8.** Amino acid identity percentages between the *16S rRNA* genes of the examined *Escherichia coli* isolate strain S2, and other *E. coli* isolates on the GenBank. ID: identity, ▪ Our examined *E. coli* isolate.


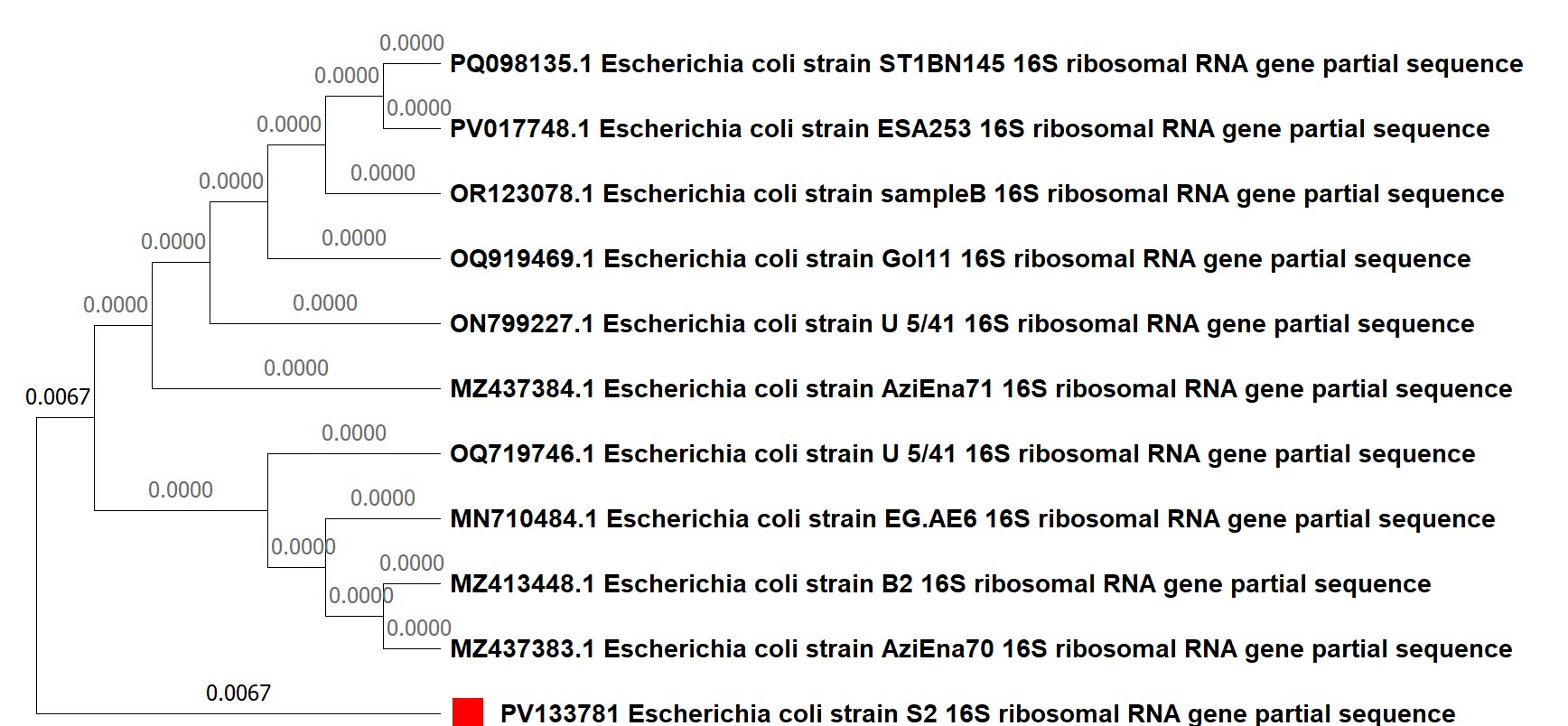


**Figure S9.** Phylogenetic tree of the examined *Escherichia coli* isolate strain S2 based on the *16S rRNA* gene partial sequence generated via the Neighbor-Joining technique. ▪ Our examined *E. coli* isolate.


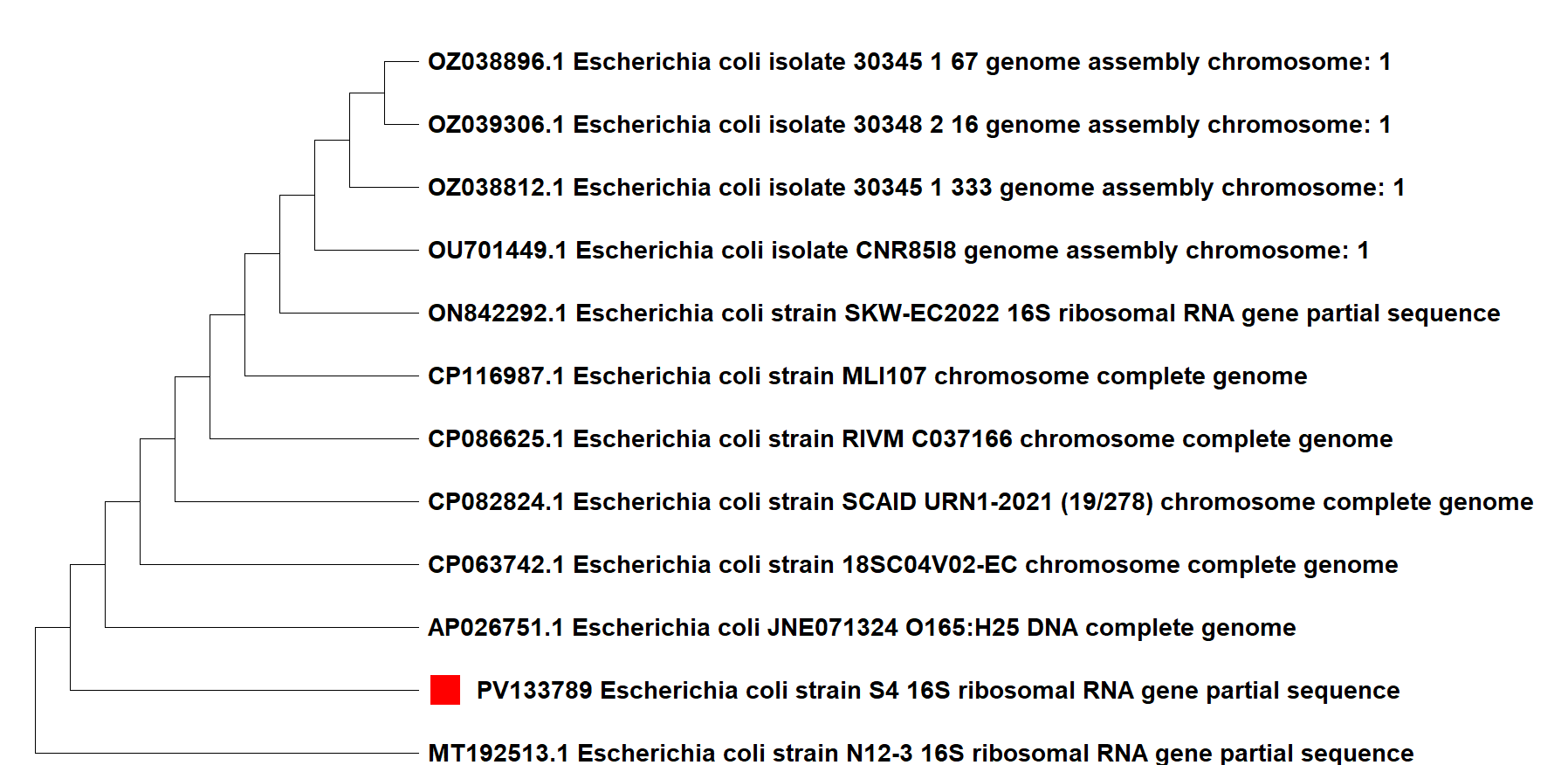
**Figure S10.** Phylogenetic tree of the examined *Escherichia coli* isolate strain S4 based on the *16S rRNA* gene partial sequence generated via the Neighbor-Joining technique. ▪ Our examined *E. coli* isolate.


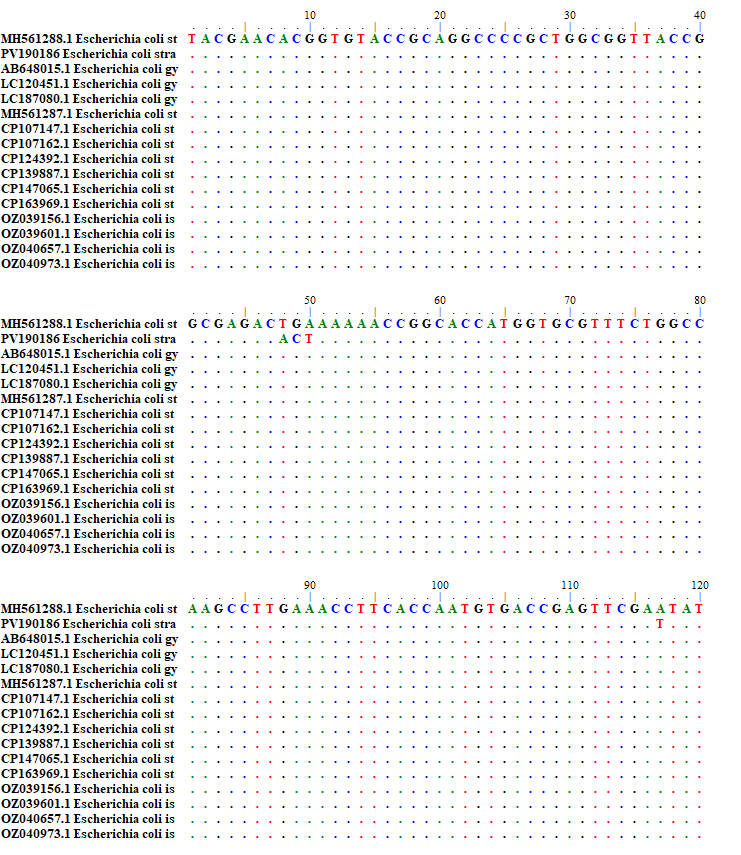


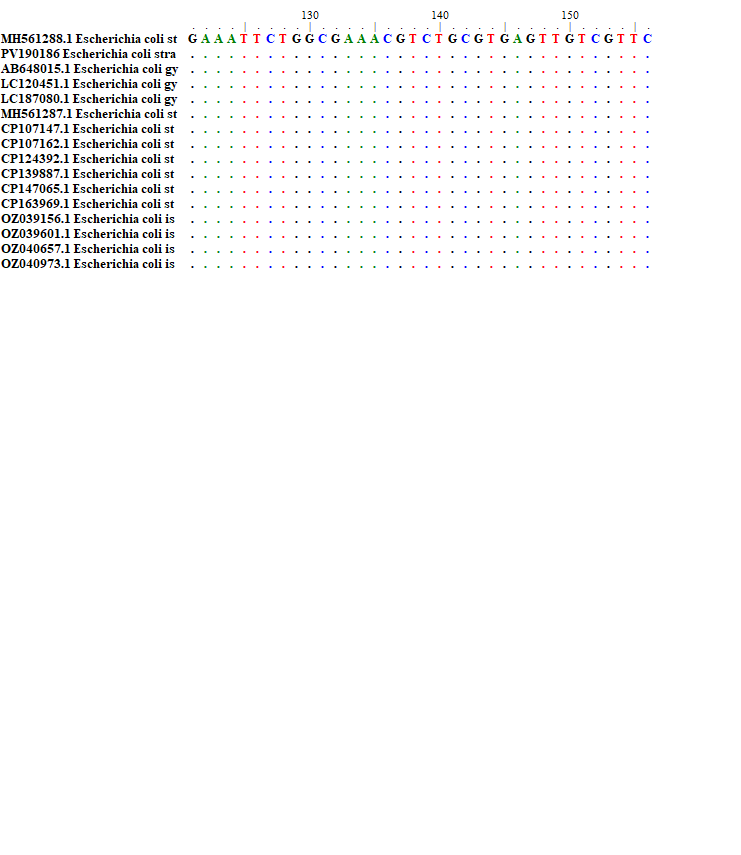


**Figure S11.** Nucleotide sequence alignments of the *gyrB* gene of the examined *Escherichia coli* isolate strain S1 with PV190186 accession number. The sequences are indicated by their accession numbers.


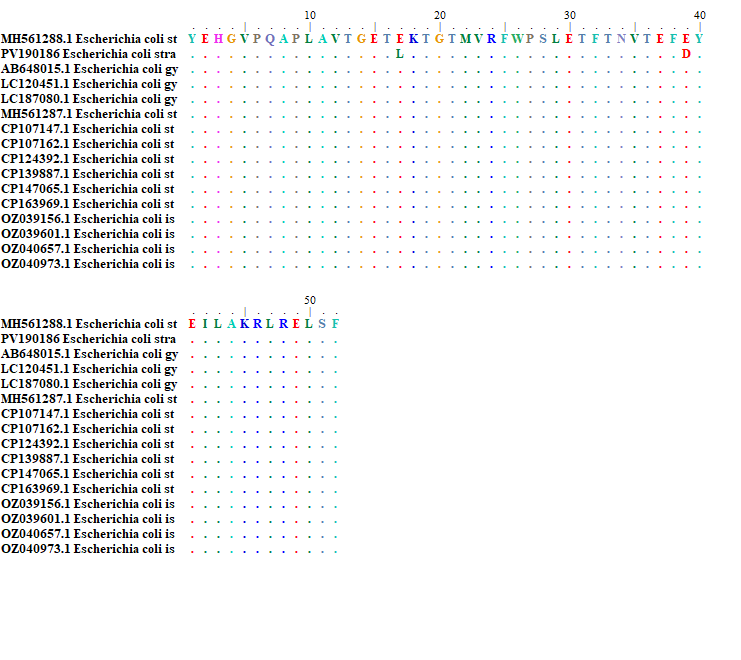


**Figure S12.** Amino acid sequence alignments of the *gyrB* gene of the examined *Escherichia coli* isolate strain S1 with PV190186 accession number. The sequences are indicated by their accession numbers.


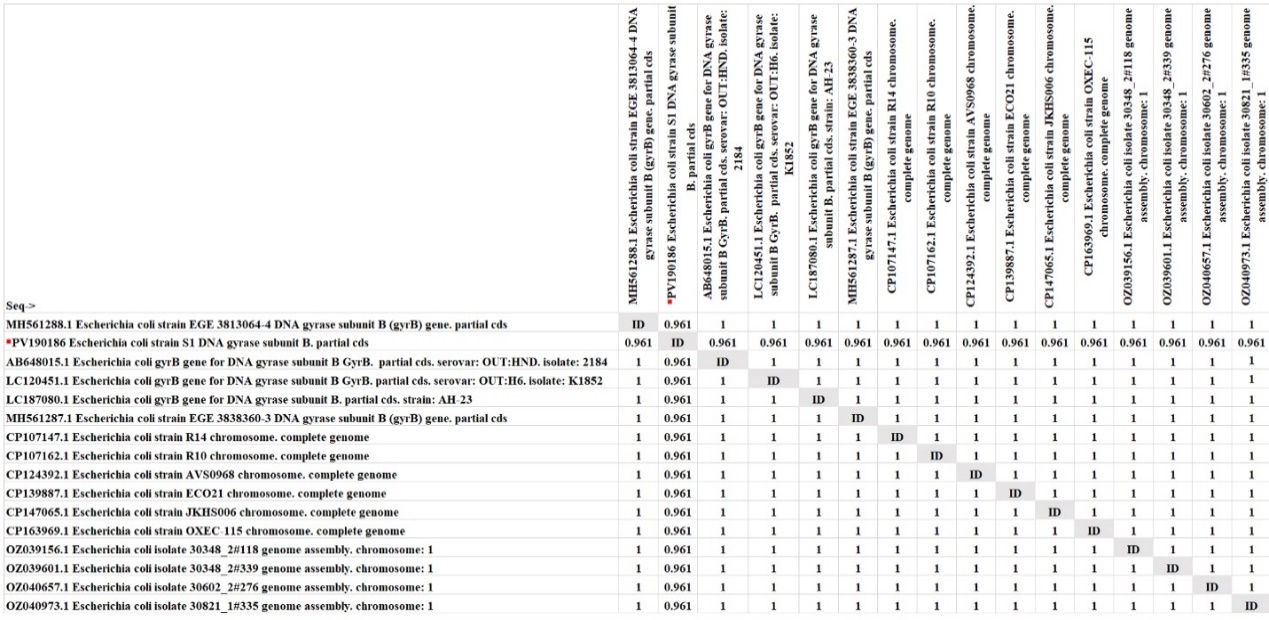


**Figure S13.** Amino acid identity percentages between the *gyrB* genes of the examined *Escherichia coli* isolate strain S1, and other *E. coli* isolates on the GenBank. ID: identity. ▪ Our examined *E. coli* isolate.


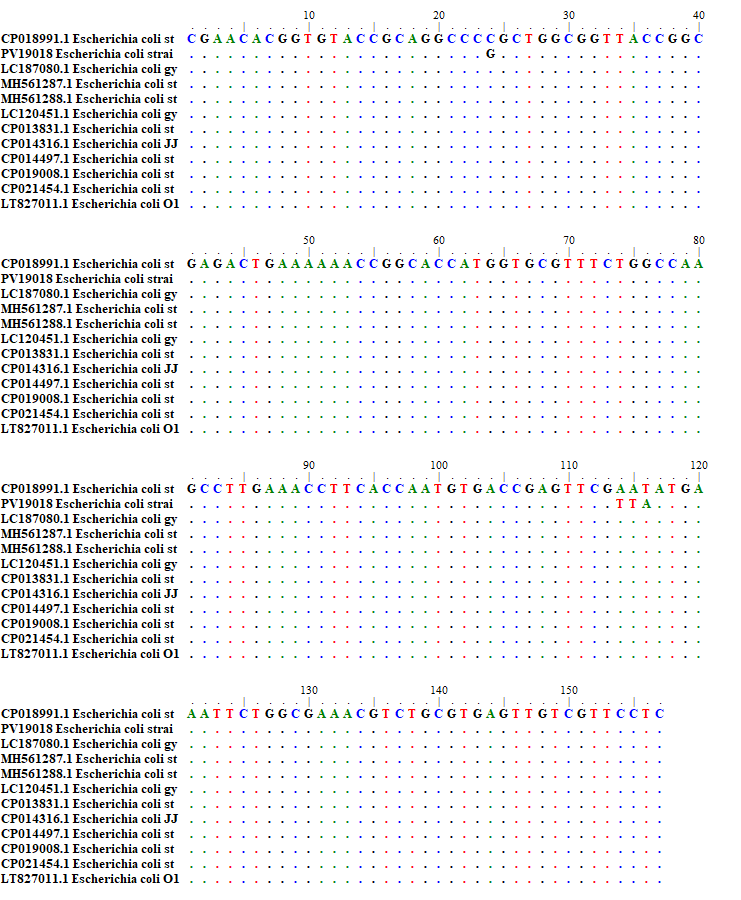


**Figure S14.** Nucleotide sequence alignments of the *gyrB* gene of the examined *Escherichia coli* isolate strain S2 with PV190187 accession number. The sequences are indicated by their accession numbers.


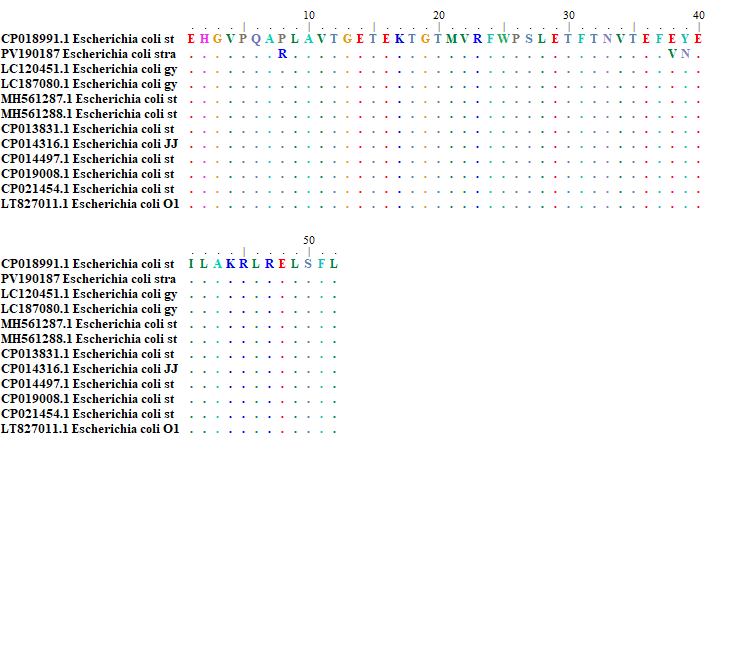


**Figure S15.** Amino acid sequence alignments of the *gyrB* gene of the examined *Escherichia coli* isolate strain S2 with PV190187 accession number. The sequences are indicated by their accession numbers.


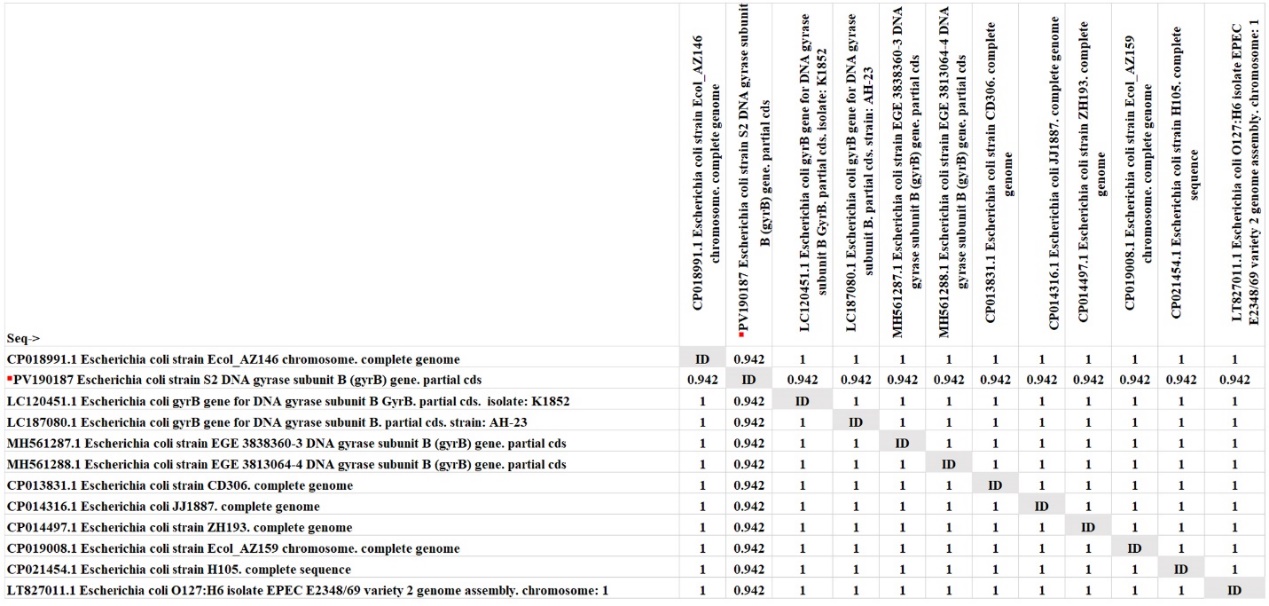


**Figure S16.** Amino acid identity percentages between the *gyrB* genes of the examined *Escherichia coli* isolate strain S2, and other *E. coli* isolates on the GenBank. ID: identity, ▪ Our examined *E. coli* isolate.


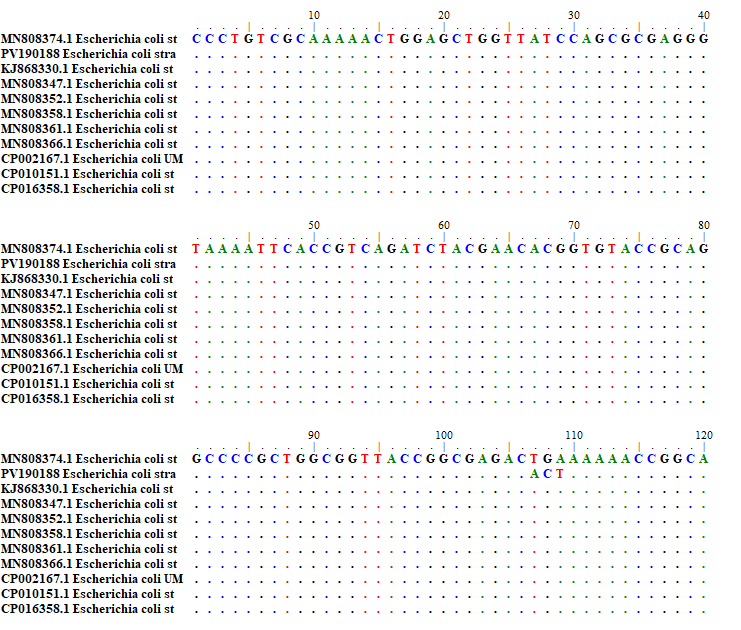


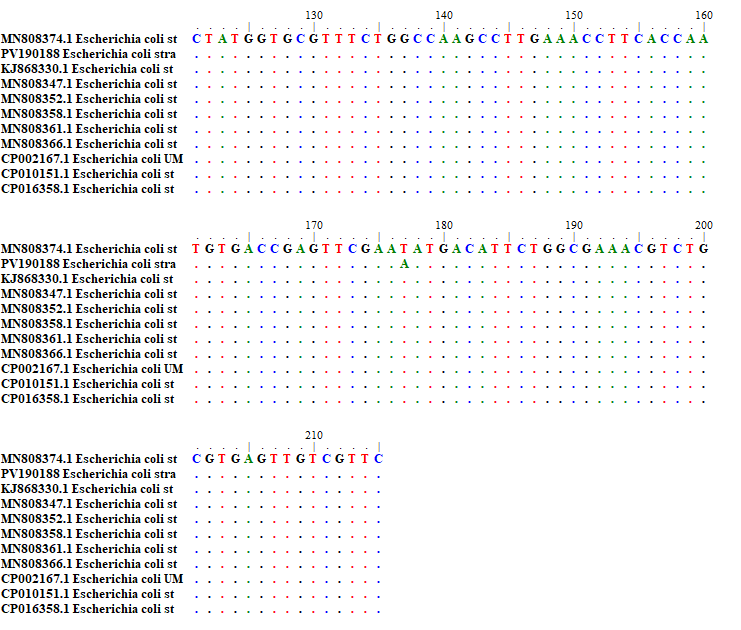


**Figure S17.** Nucleotide sequence alignments of the *gyrB* gene of the examined *Escherichia coli* isolate strain S3 with PV190188 accession number. The sequences are indicated by their accession numbers.


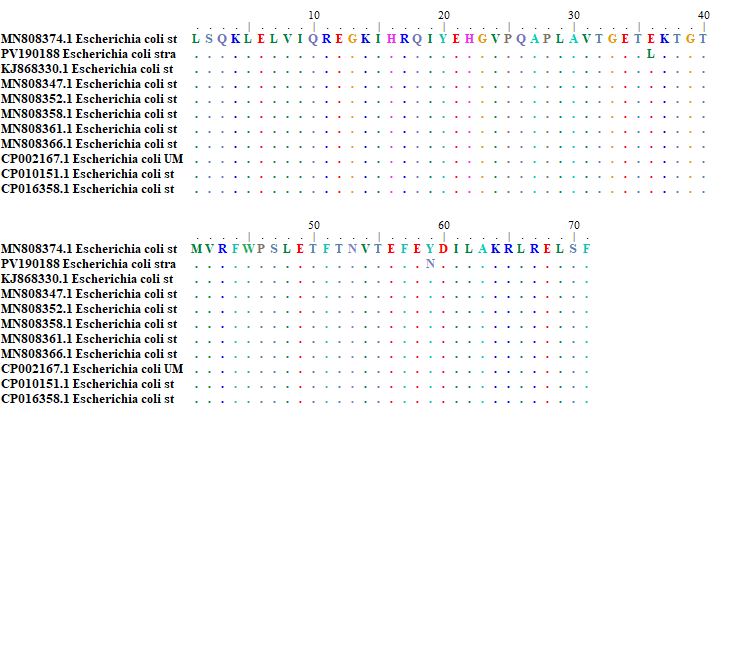


**Figure S18.** Amino acid sequence alignments of the *gyrB* gene of the examined *Escherichia coli* isolate strain S3 with PV190188 accession number. The sequences are indicated by their accession numbers.


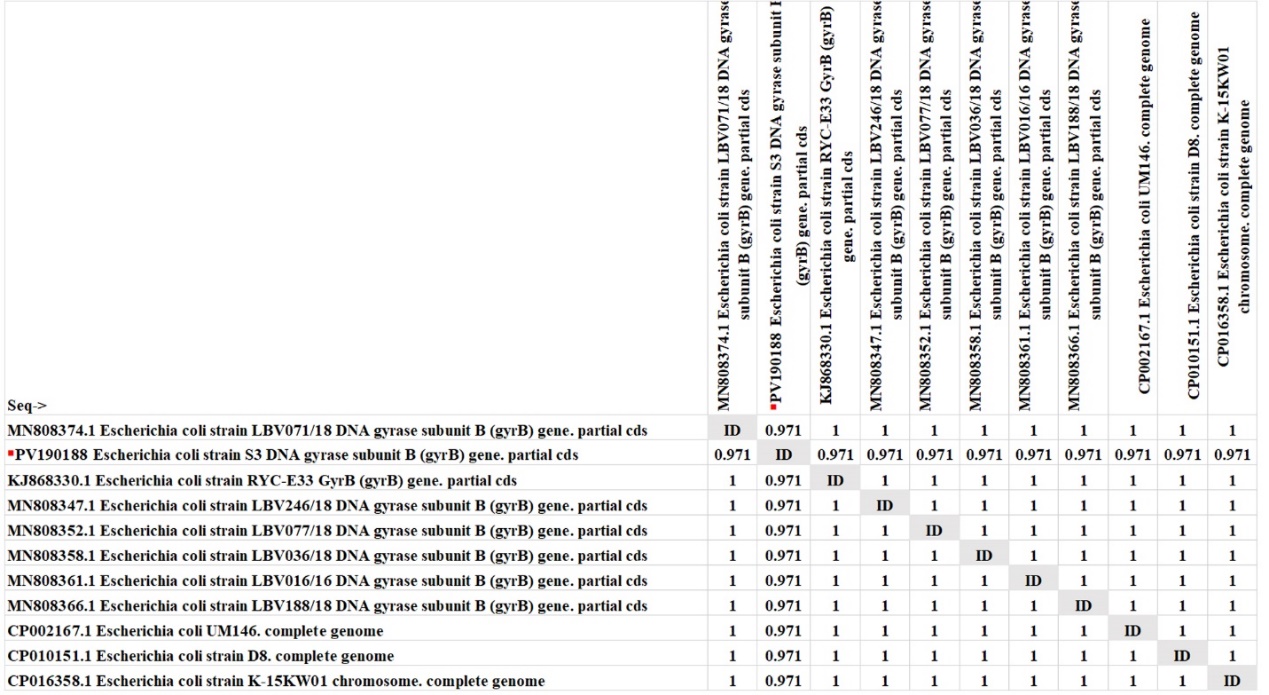


**Figure S19.** Amino acid identity percentages between the *gyrB* genes of the examined *Escherichia coli* isolate strain S3, and other *E. coli* isolates on the GenBank. ID: identity. ▪ Our examined *E. coli* isolate.


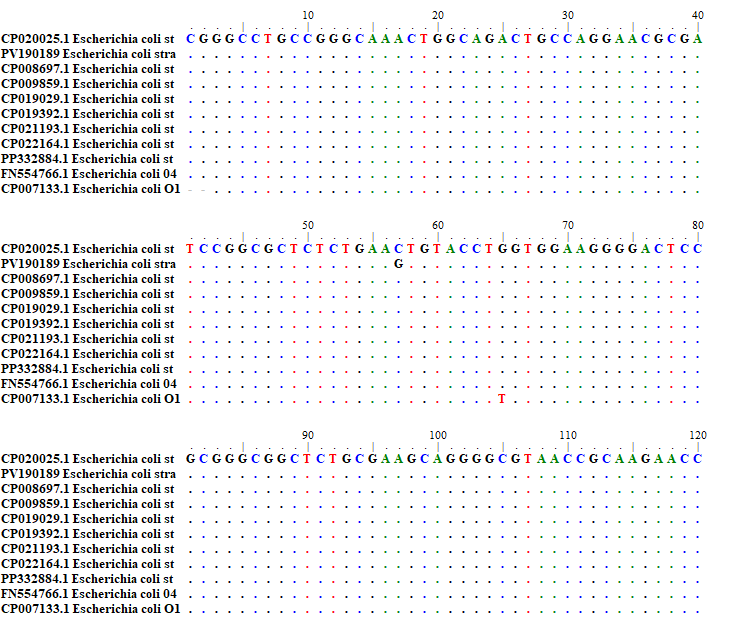

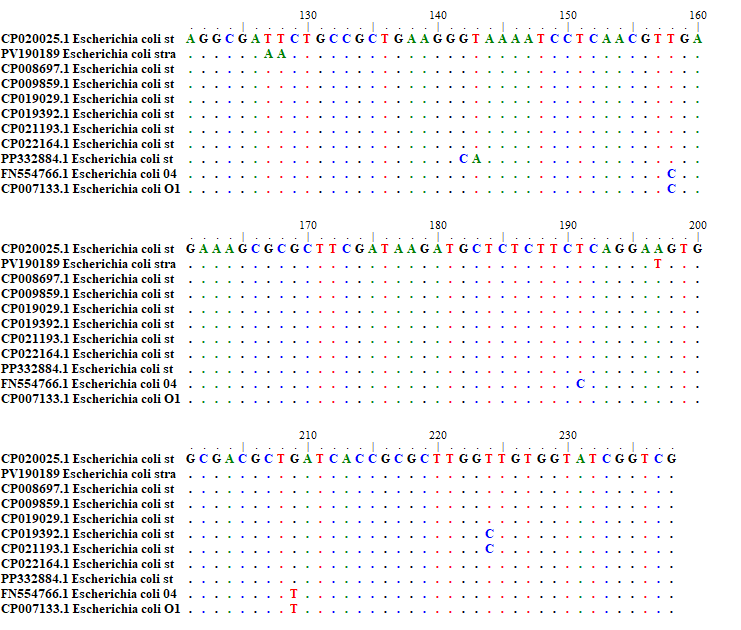


**Figure S20.** Nucleotide sequence alignments of the *gyrB* gene of the examined *Escherichia coli* isolate strain S4 with PV190189 accession number. The sequences are indicated by their accession numbers.


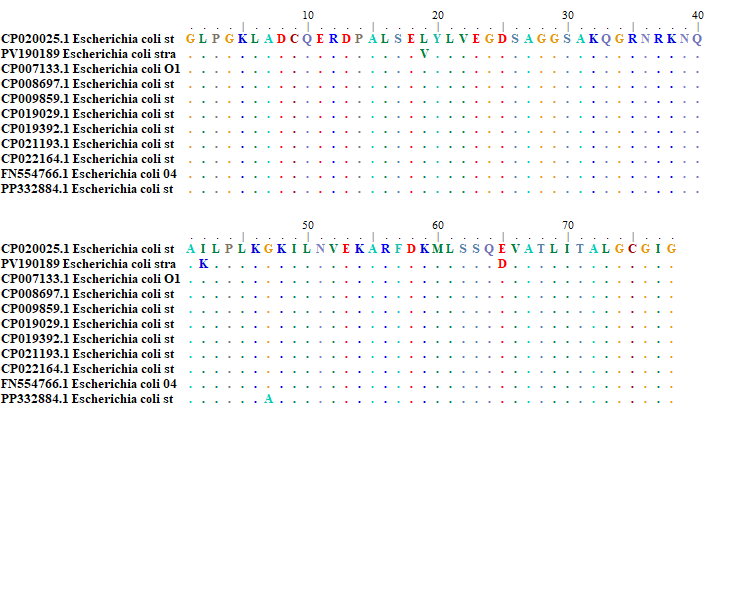


**Figure S21.** Amino acid sequence alignments of the *gyrB* gene of the examined *Escherichia coli* isolate strain S4 with PV190189 accession number. The sequences are indicated by their accession numbers.


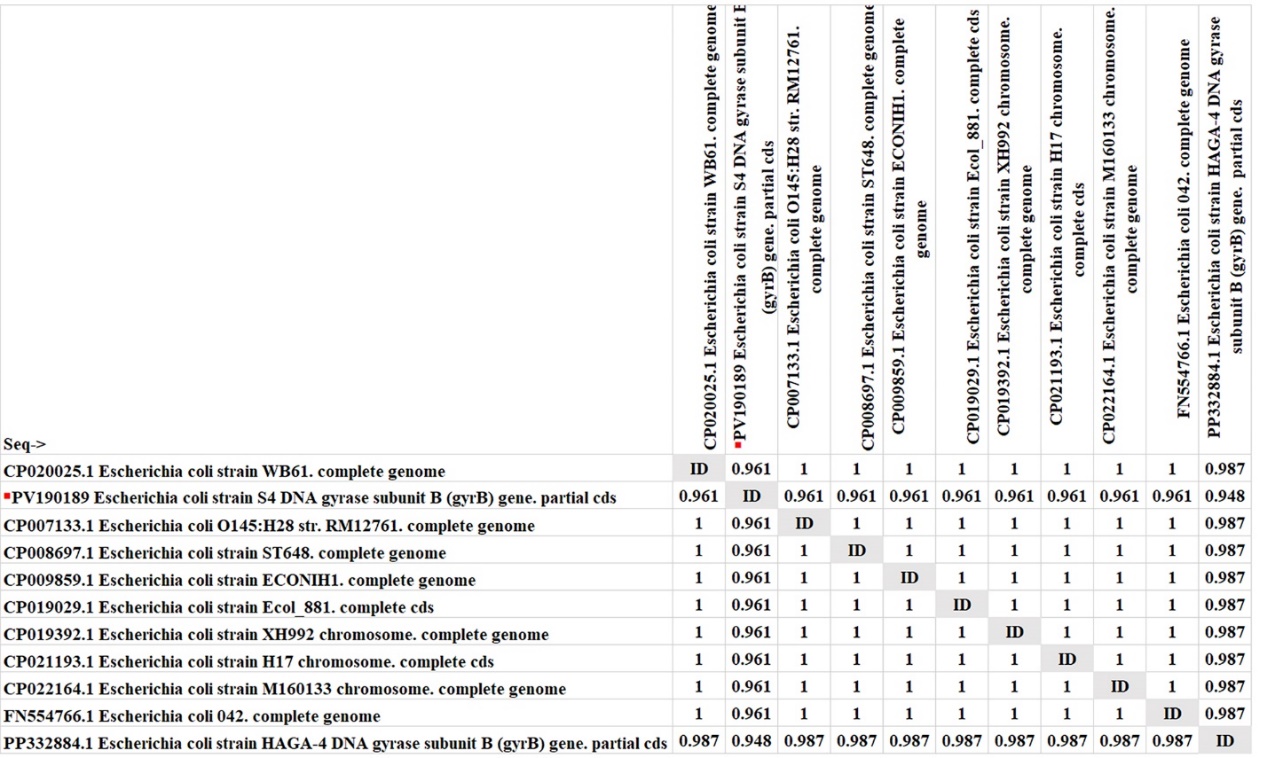


**Figure S22.** Amino acid identity percentages between the *gyrB* genes of the examined *Escherichia coli* isolate strain S4, and other *E. coli* isolates on the GenBank. ID: identity. ▪ Our examined *E. coli* isolate.


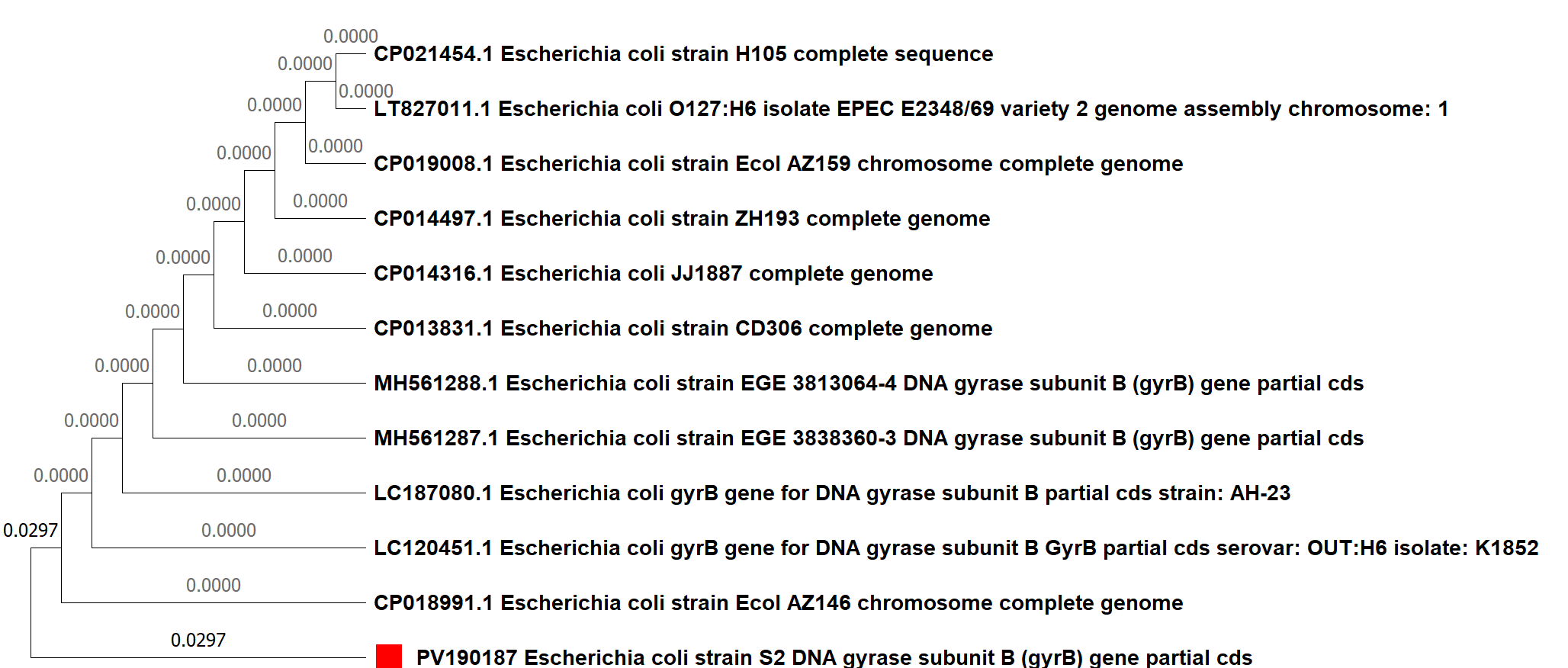


**Figure S23.** Phylogenetic tree of the examined *Escherichia coli* isolate strain S2 based on the *gyrB* gene partial sequence generated via the Neighbor-Joining technique. ▪ Our examined *E. coli* isolate.


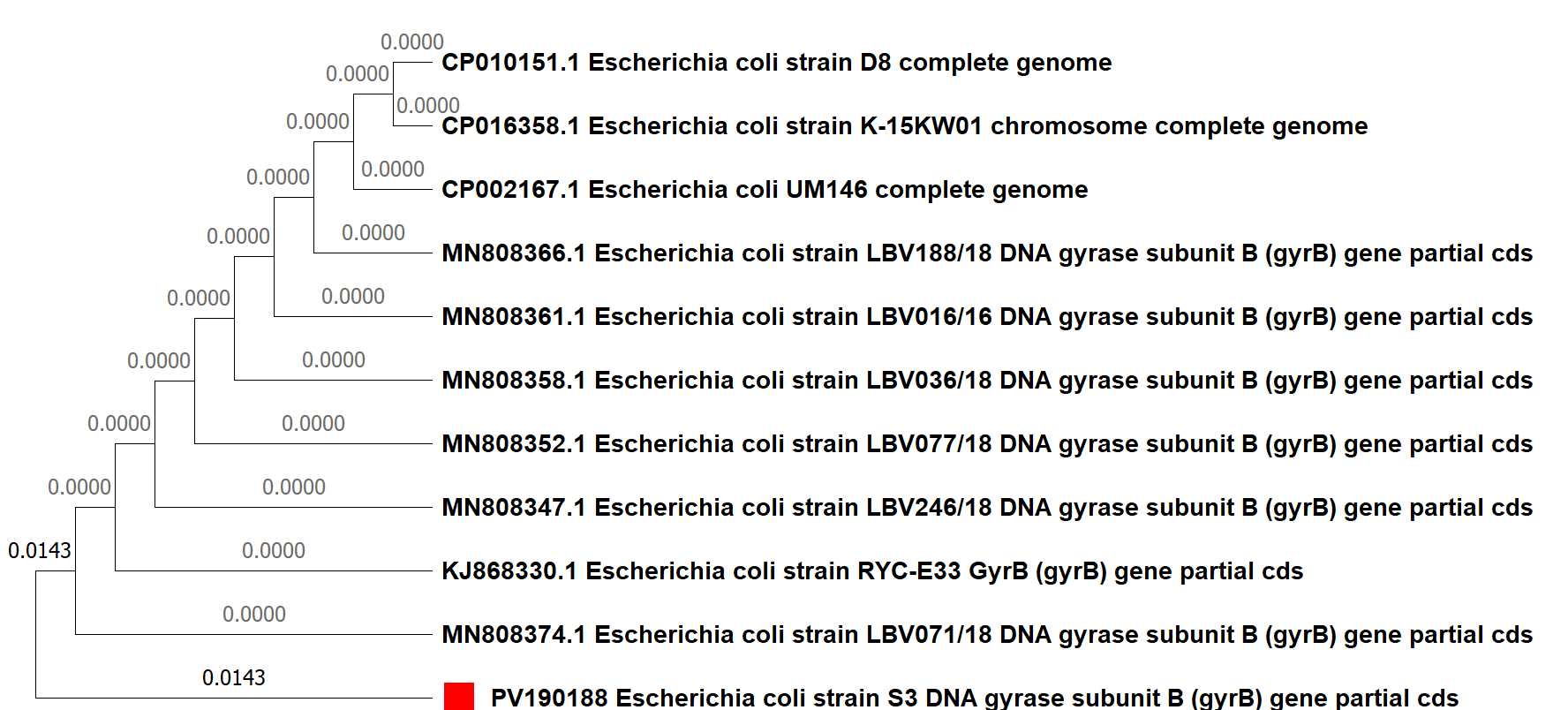


**Figure S24.** Phylogenetic tree of the examined *Escherichia coli* isolate strain S3 based on the *gyrB* gene partial sequence generated via the Neighbor-Joining technique. ▪ Our examined *E. coli* isolate.


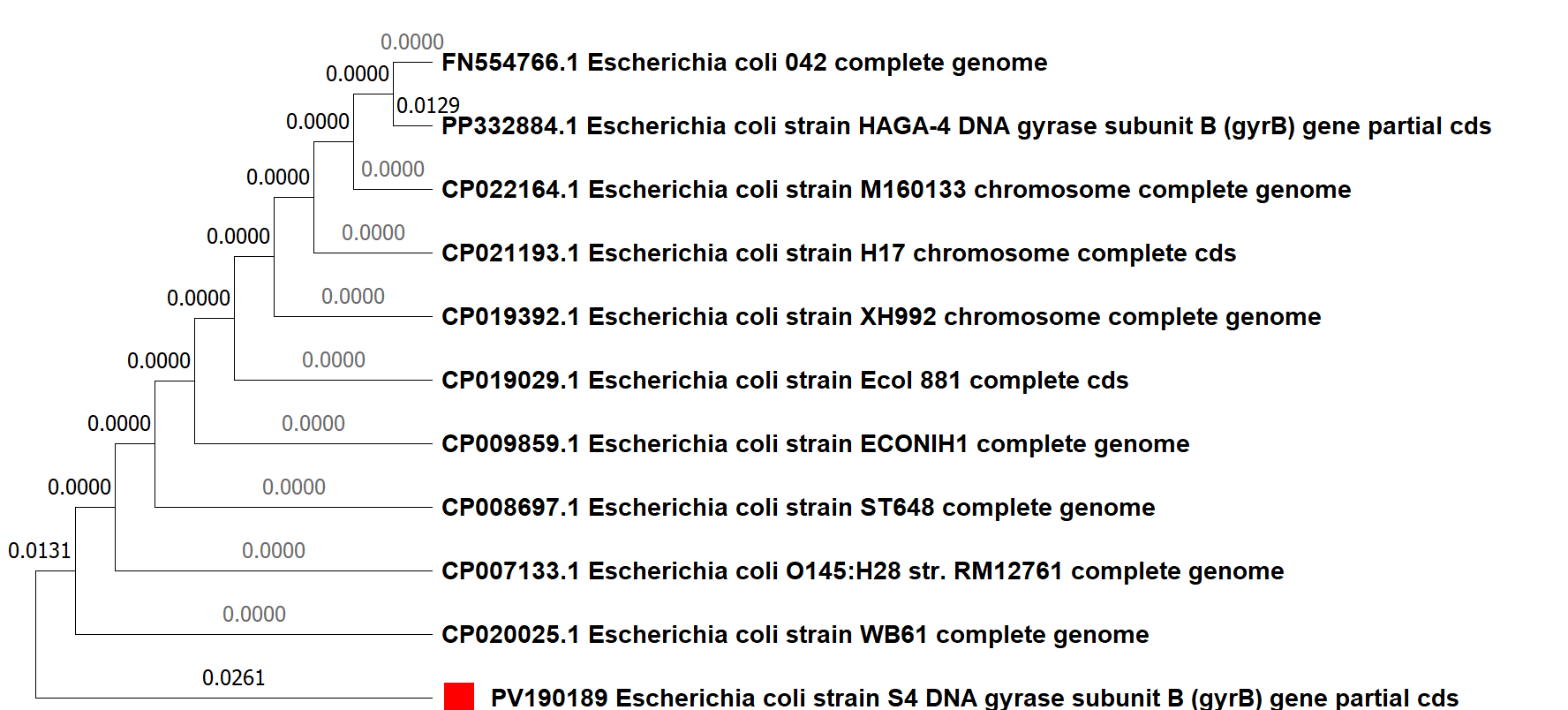


**Figure S25.** Phylogenetic tree of the examined *Escherichia coli* isolate strain S4 based on the *gyrB* gene partial sequence generated via the Neighbor-Joining technique. ▪ Our examined *E. coli* isolate.


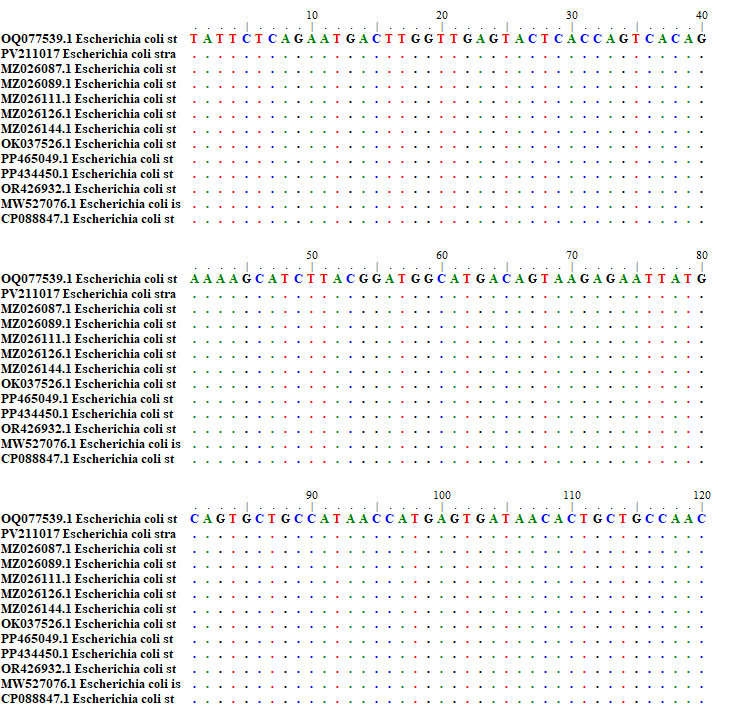

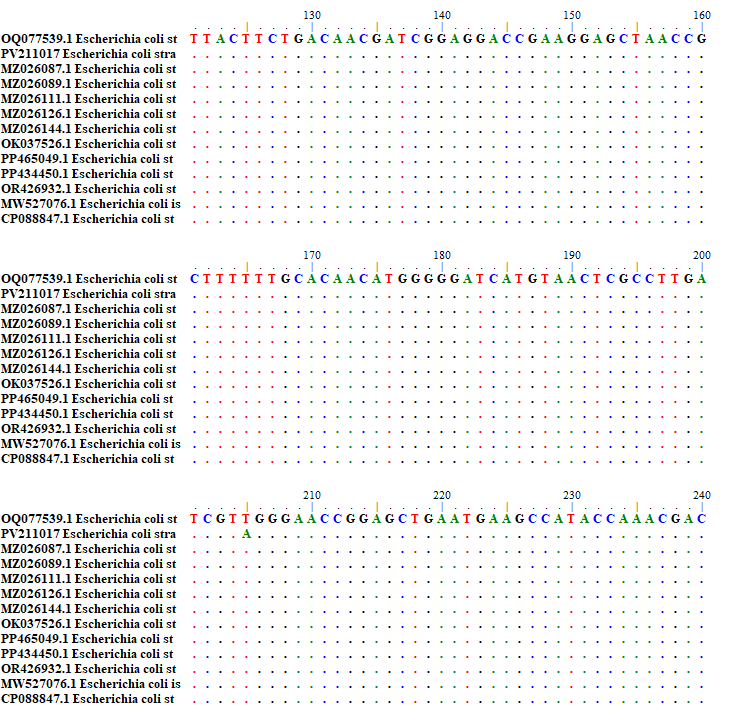


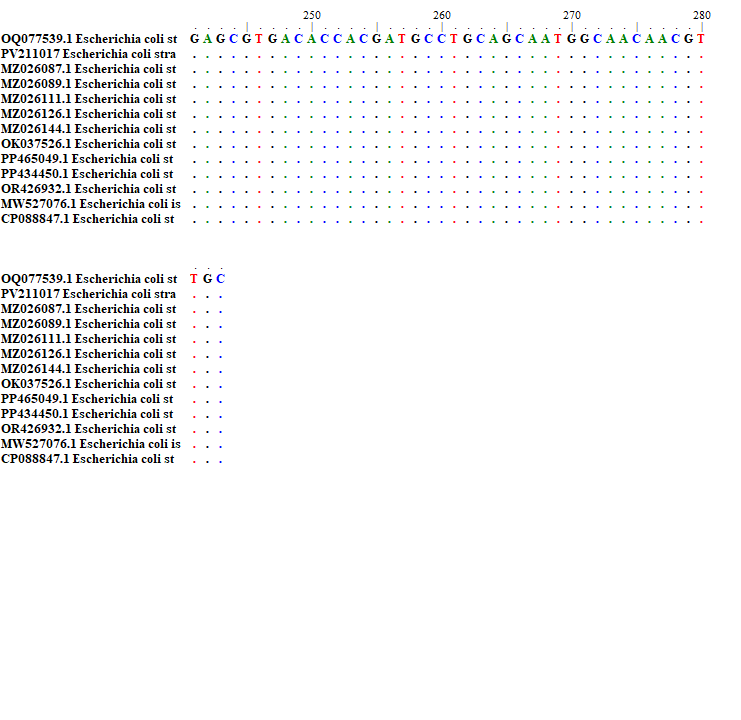


**Figure S26.** Nucleotide sequence alignments of the *blaTEM* gene of the examined *Escherichia coli* isolate strain S1 with accession number of PV211017. The sequences are indicated by their accession numbers.


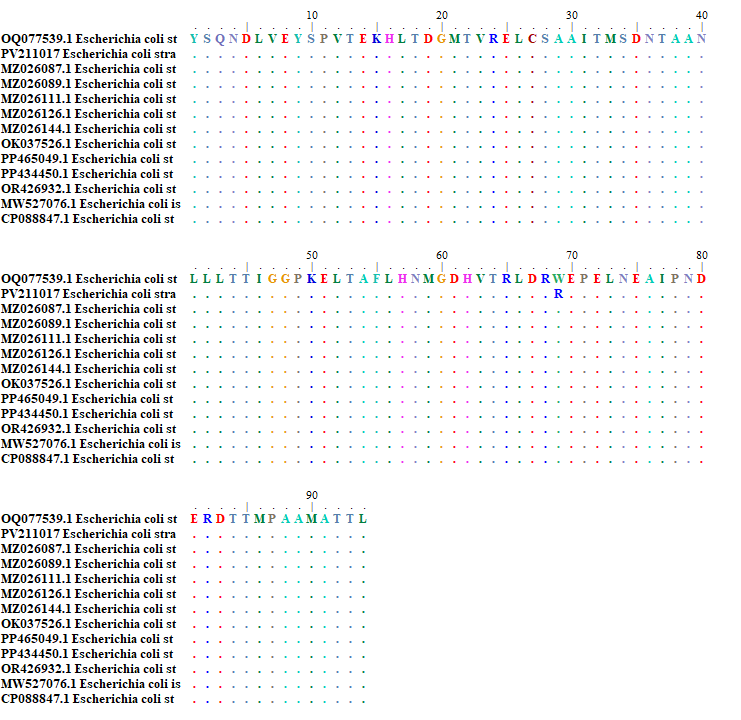


**Figure S27.** Amino acid sequence alignments of the *blaTEM* gene of the examined *Escherichia coli* isolate strain S1 with accession number of PV211017. The sequences are indicated by their accession numbers.


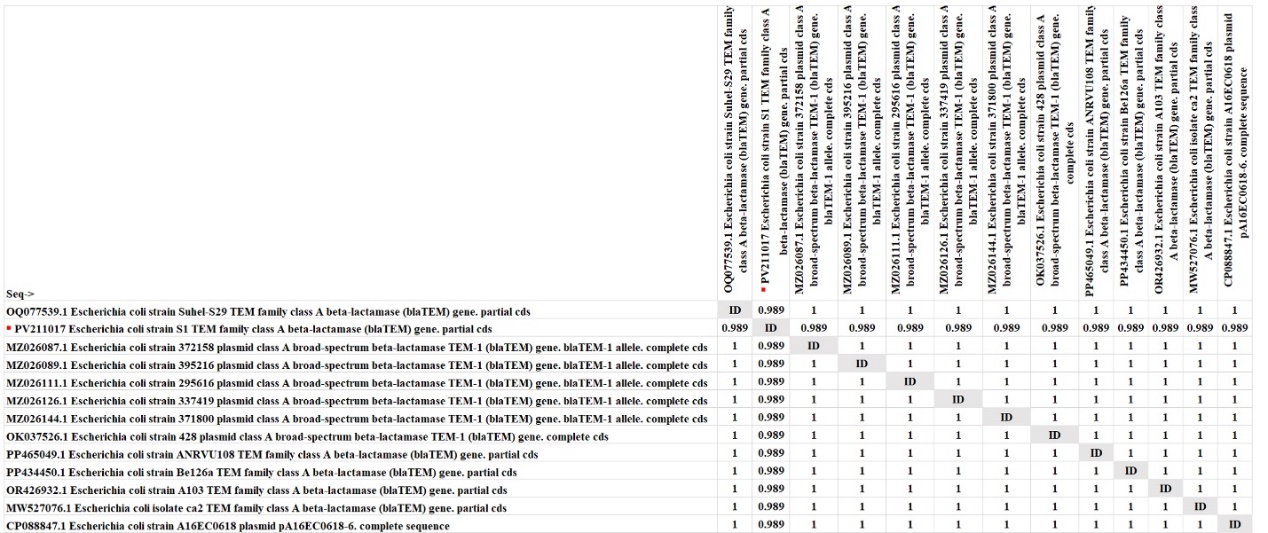


**Figure S28.** Amino acid identity percentages between the *blaTEM* genes of the examined *Escherichia coli* isolate strain S1, and other *E. coli* isolates on the GenBank. ID: identity. ▪ Our examined *E. coli* isolate.


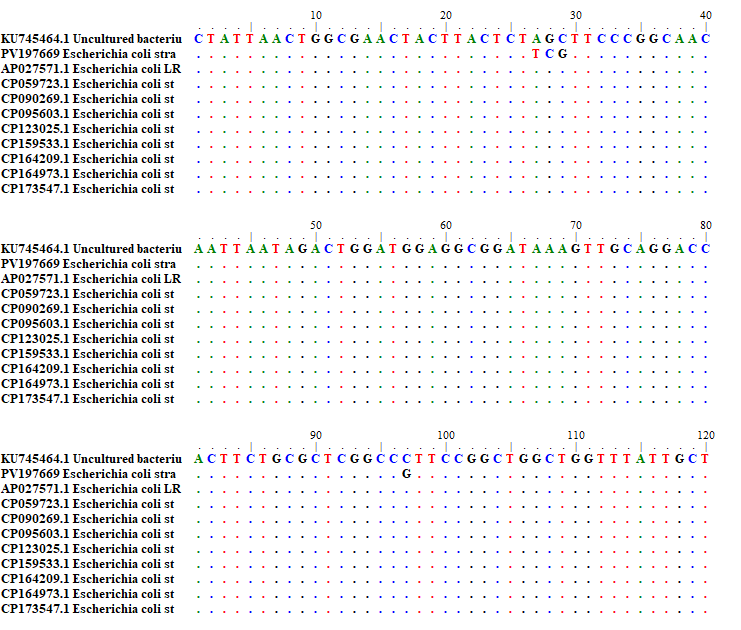

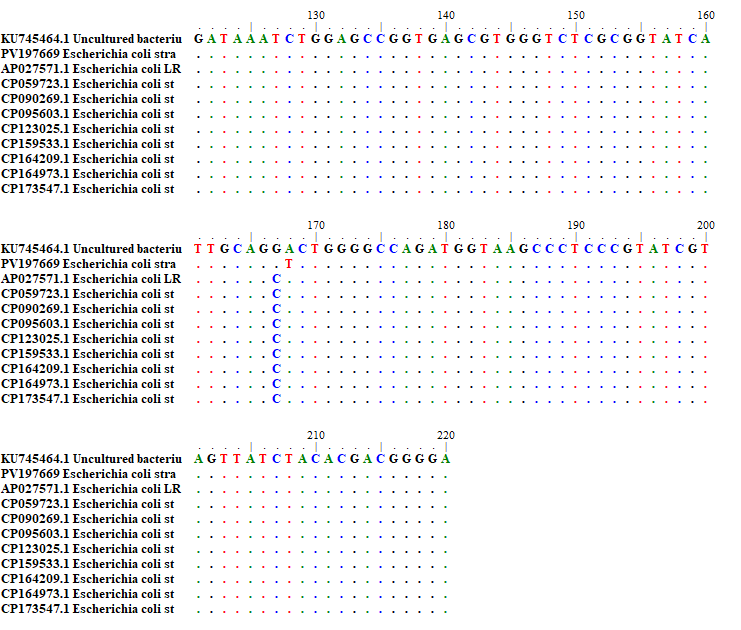


**Figure S29.** Nucleotide sequence alignments of the *blaTEM* gene of the examined *Escherichia coli* isolate strain S2 with PV197669 accession number. The sequences are indicated by their accession numbers.


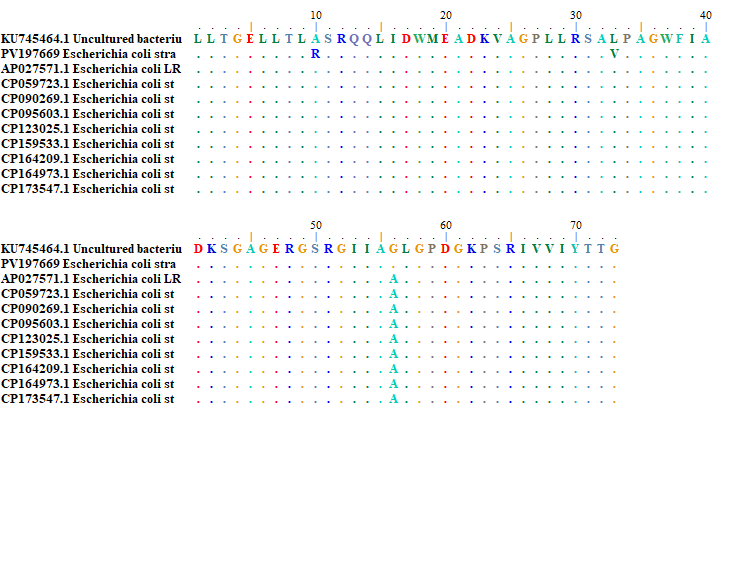


**Figure S30.** Amino acid sequence alignments of the *blaTEM* gene of the examined *Escherichia coli* isolate strain S2 with PV197669 accession number. The sequences are indicated by their accession numbers.


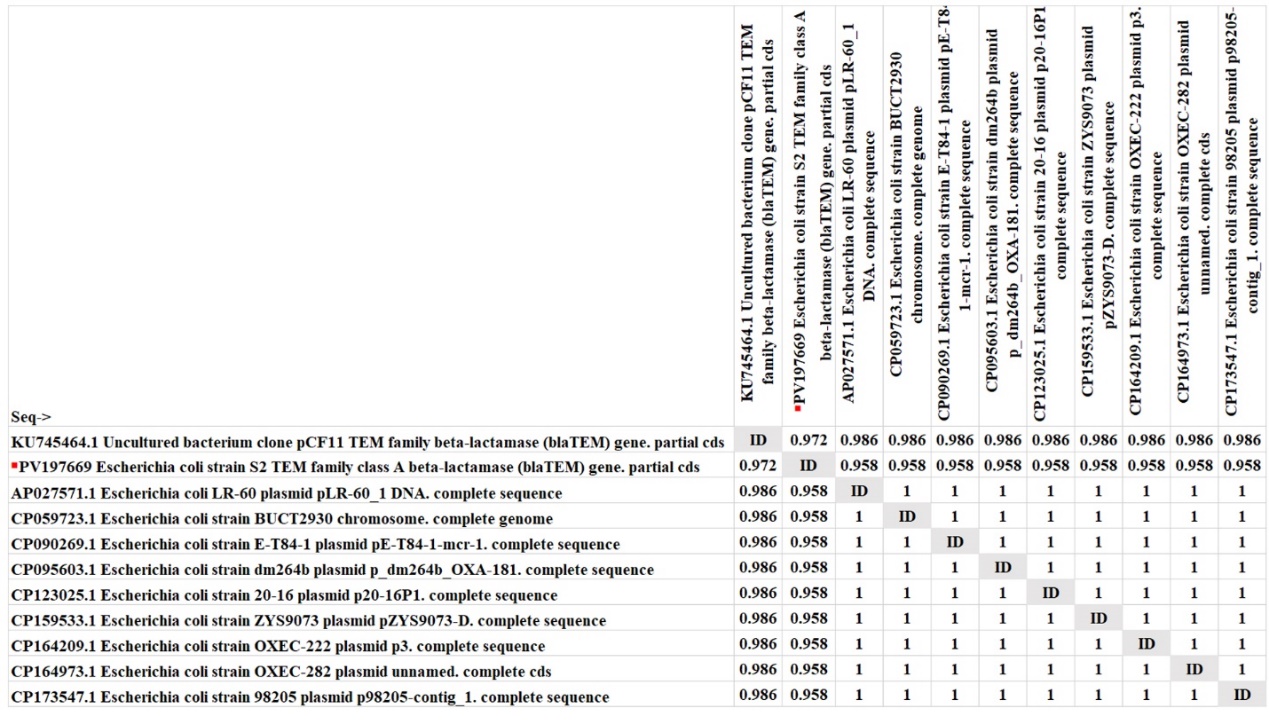


**Figure S31.** Amino acid identity percentages between the *blaTEM* genes of the examined *Escherichia coli* isolate strain S2, and other *E. coli* isolates on the GenBank. ID: identity. ▪ Our examined *E. coli* isolate.


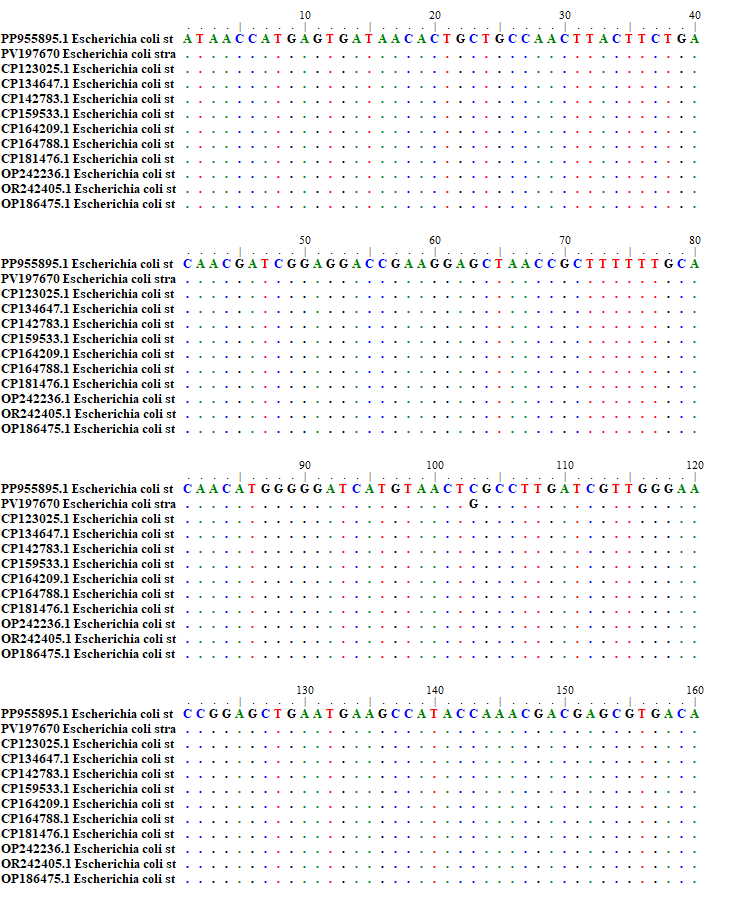

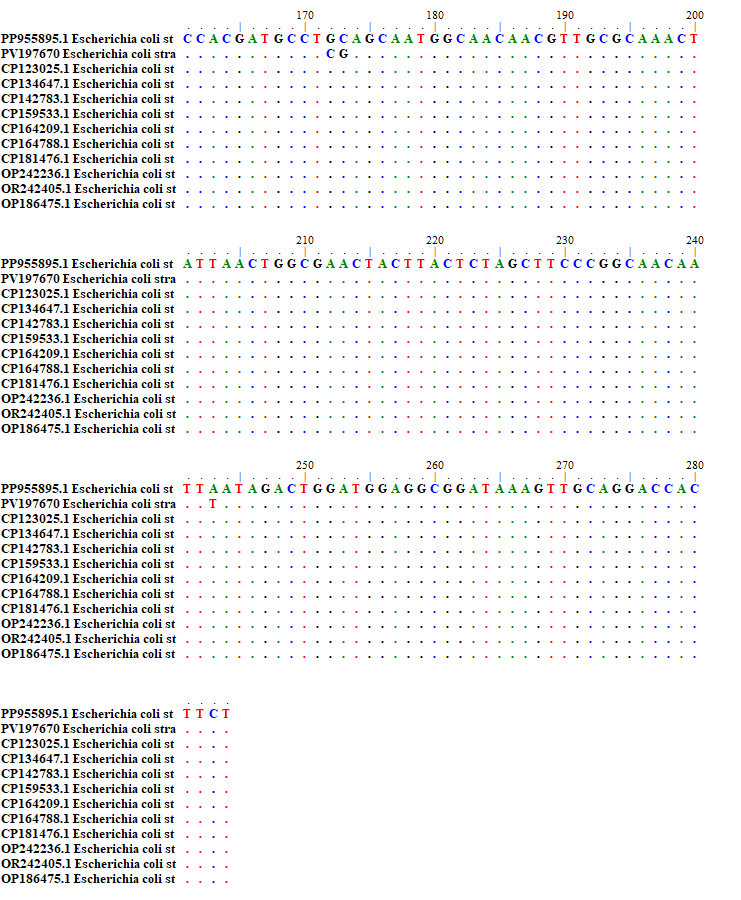


**Figure S32.** Nucleotide sequence alignments of the *blaTEM* gene of the examined *Escherichia coli* isolate strain S3 with PV197670 accession number. The sequences are indicated by their accession numbers.


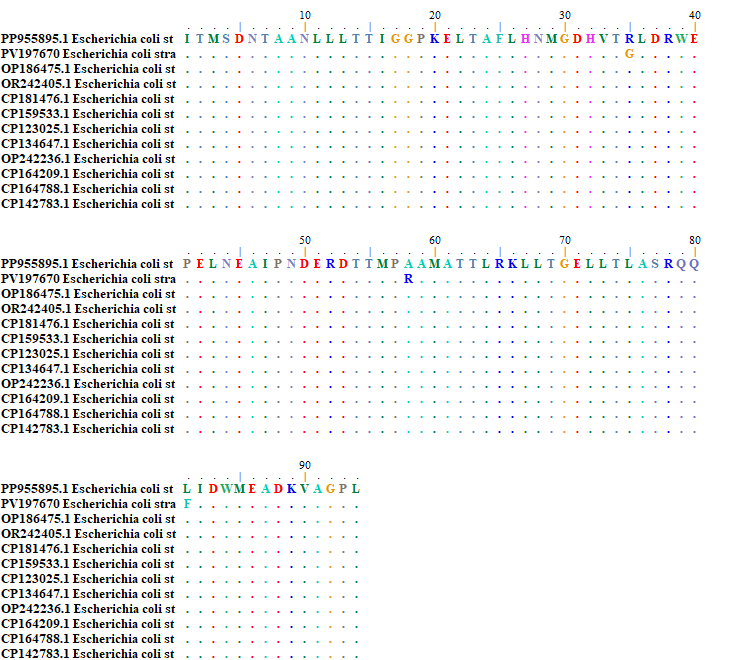


**Figure S33.** Amino acid sequence alignments of the *blaTEM* gene of the examined *Escherichia coli* isolate strain S3 with PV197670 accession number. The sequences are indicated by their accession numbers.


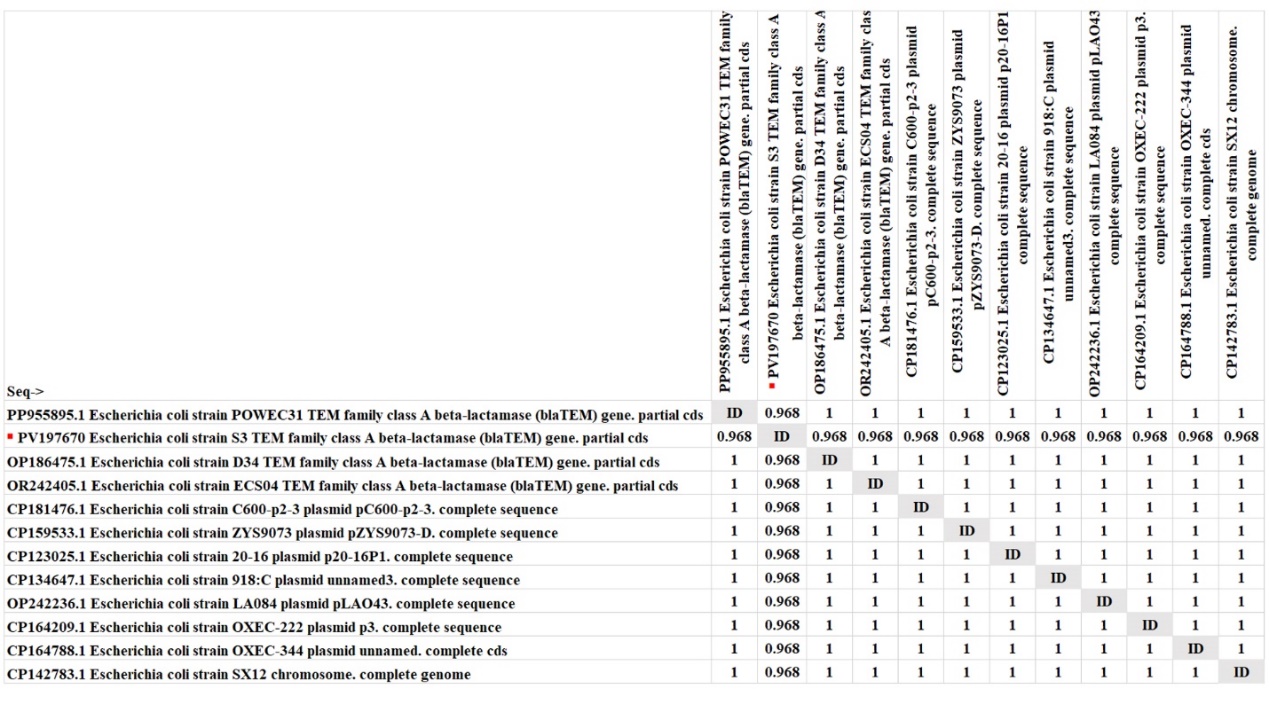


**Figure S34.** Amino acid identity percentages between the *blaTEM* genes of the examined *Escherichia coli* isolate strain S3, and other *E. coli* isolates on the GenBank. ID: identity. ▪ Our examined *E. coli* isolate.


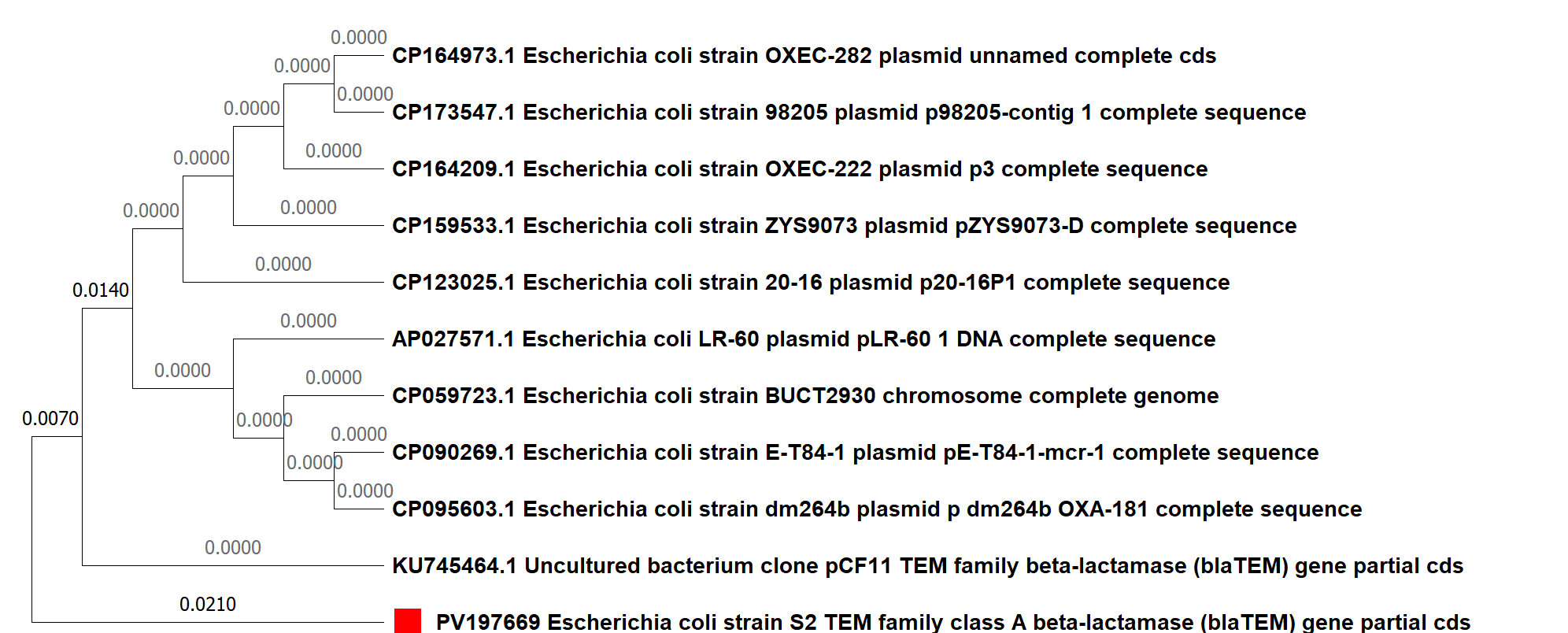


**Figure S35.** Phylogenetic tree of the examined *Escherichia coli* isolate strain S2 based on the *blaTEM* gene partial sequence generated via the Neighbor-Joining technique. ▪ Our examined *E. coli* isolate.


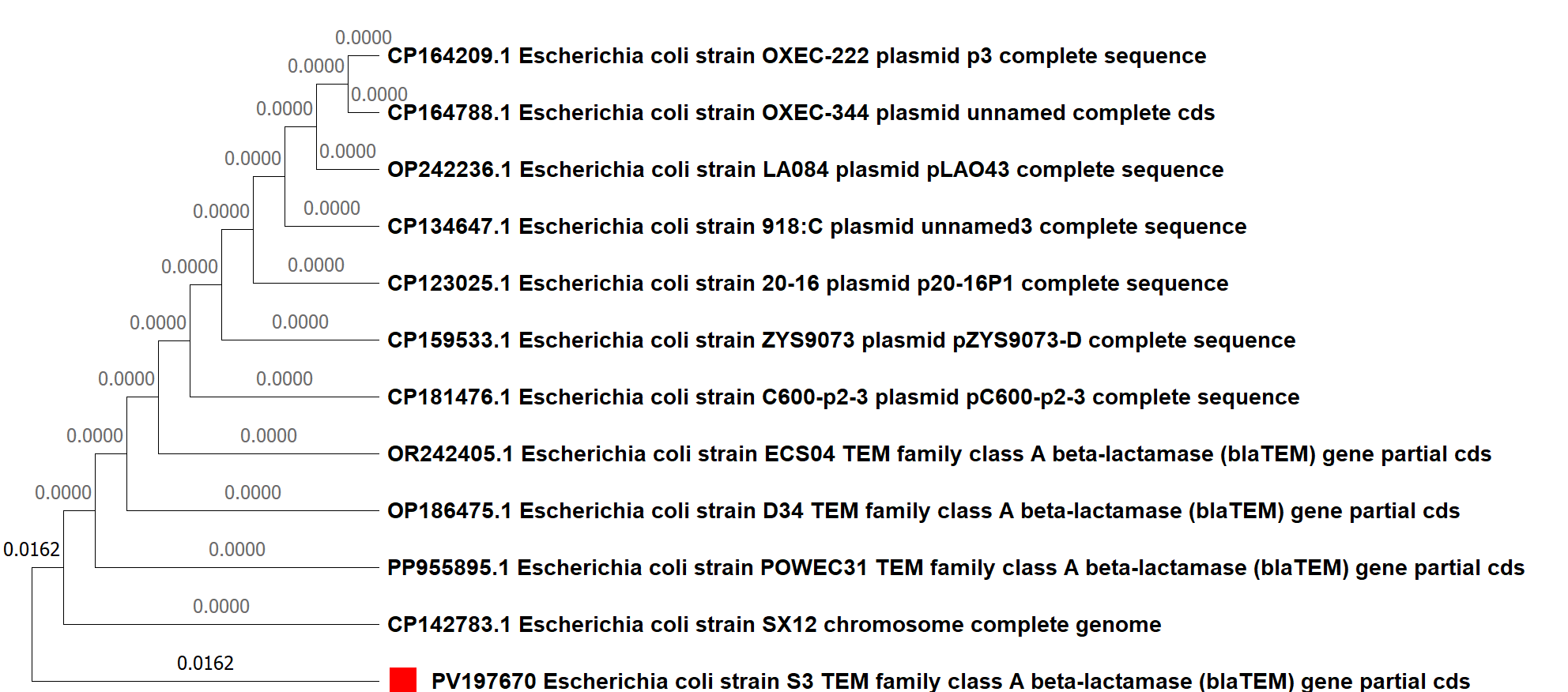


**Figure S36.** Phylogenetic tree of the examined *Escherichia coli* isolate strain S3 based on the *blaTEM* gene partial sequence generated via the Neighbor-Joining technique. ▪ Our examined *E. coli* isolate.


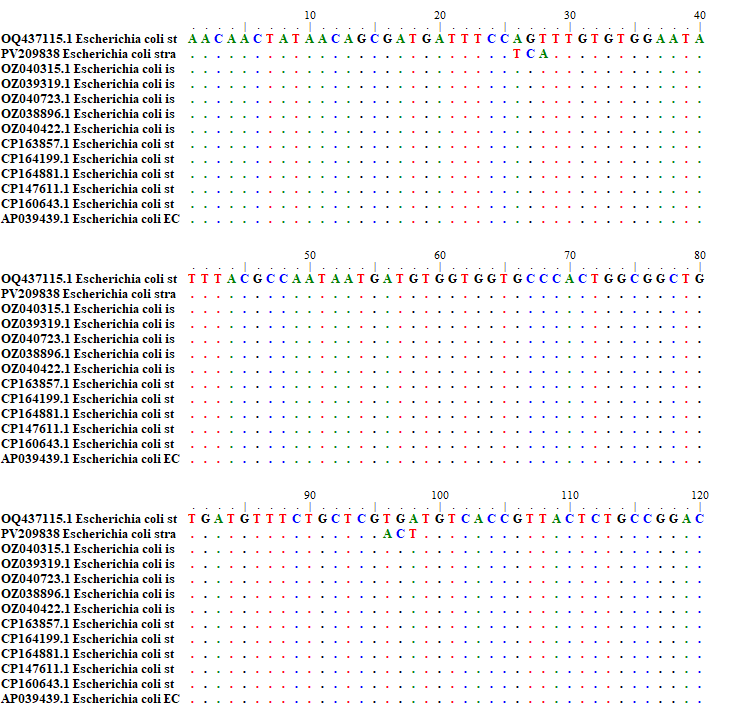

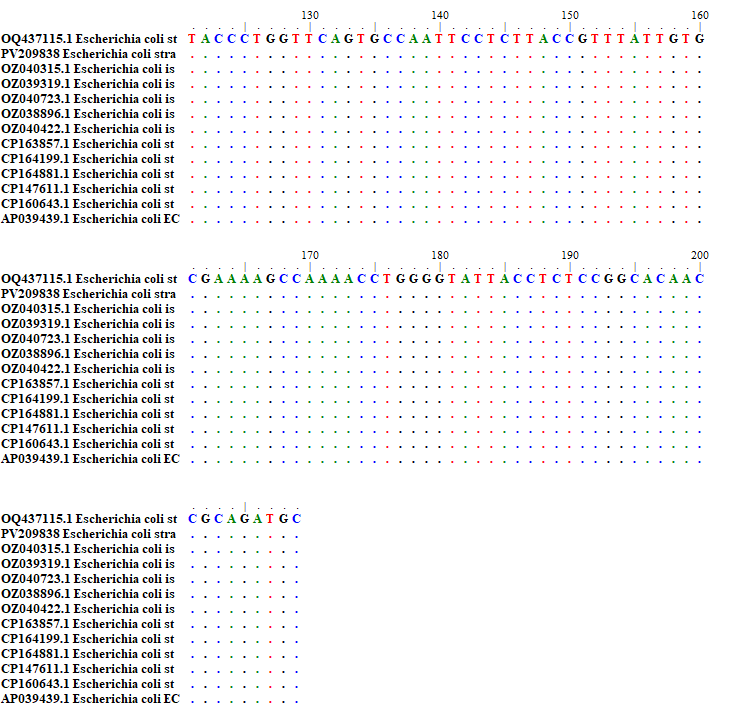


**Figure S37.** Nucleotide sequence alignments of the *fimH* gene of the examined *Escherichia coli* isolate strain S1 with PV209838 accession number. The sequences are indicated by their accession numbers.


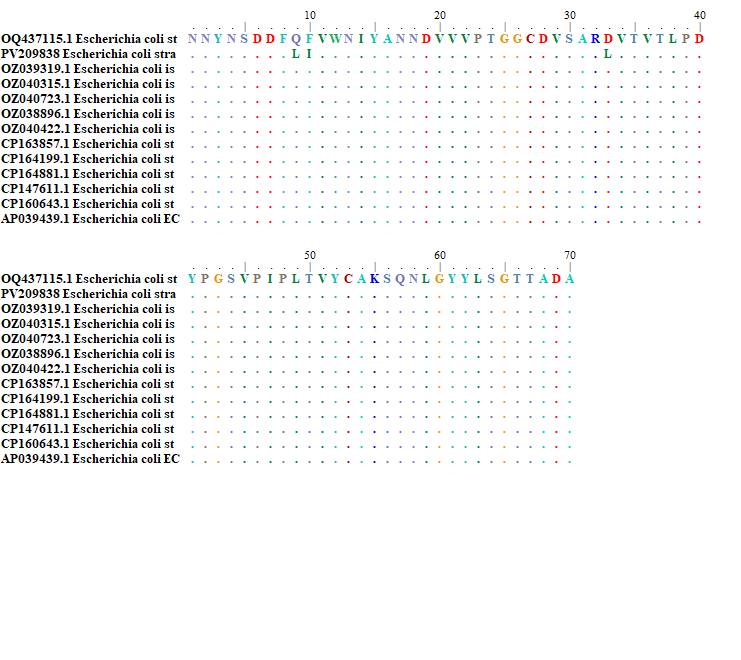


**Figure S38.** Amino acid sequence alignments of the *fimH* gene of the examined *Escherichia coli* isolate strain S1 with PV209838 accession number. The sequences are indicated by their accession numbers.


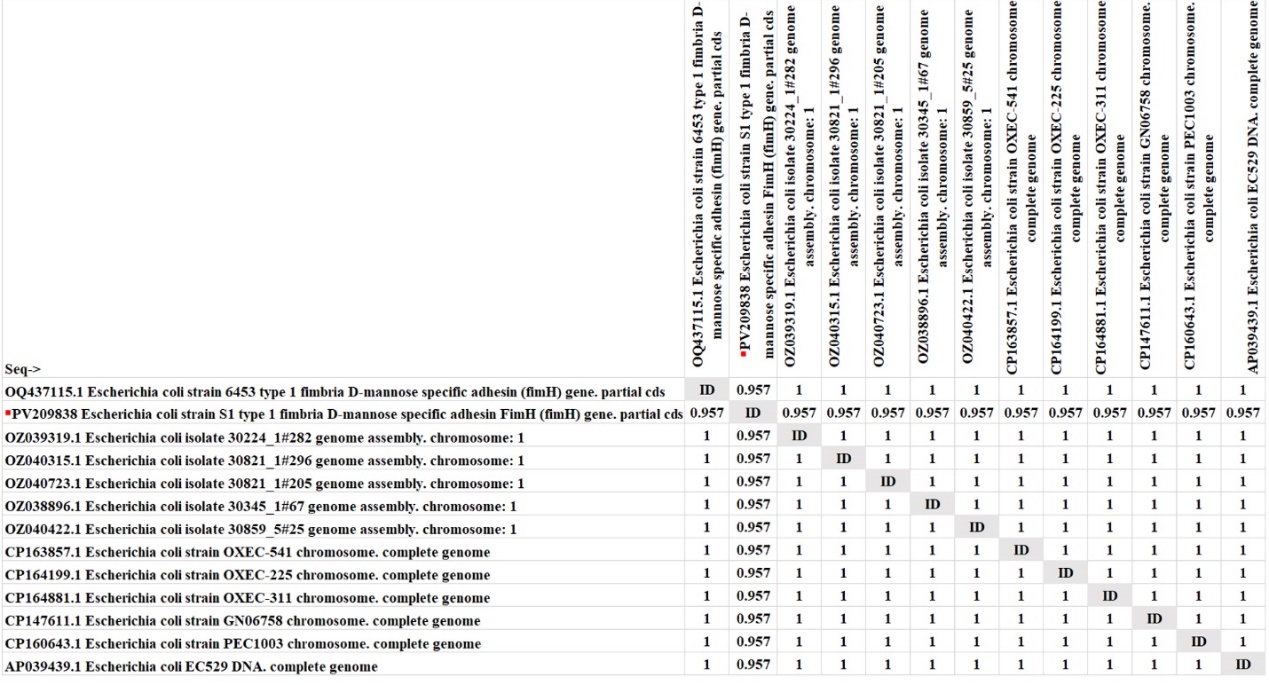


**Figure S39.** Amino acid identity percentages between the *fimH* genes of the examined *Escherichia coli* isolate strain S1, and other *E. coli* isolates on the GenBank. ID: identity. ▪ Our examined *E. coli* isolate.


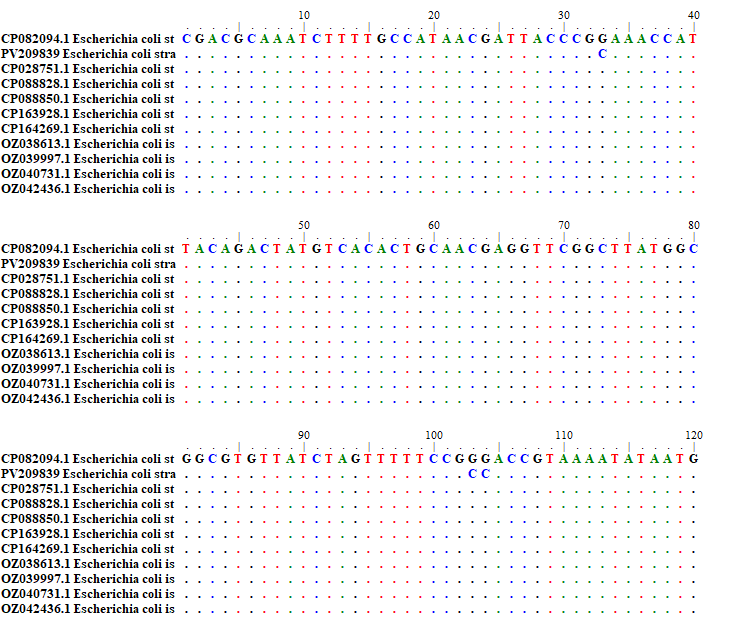

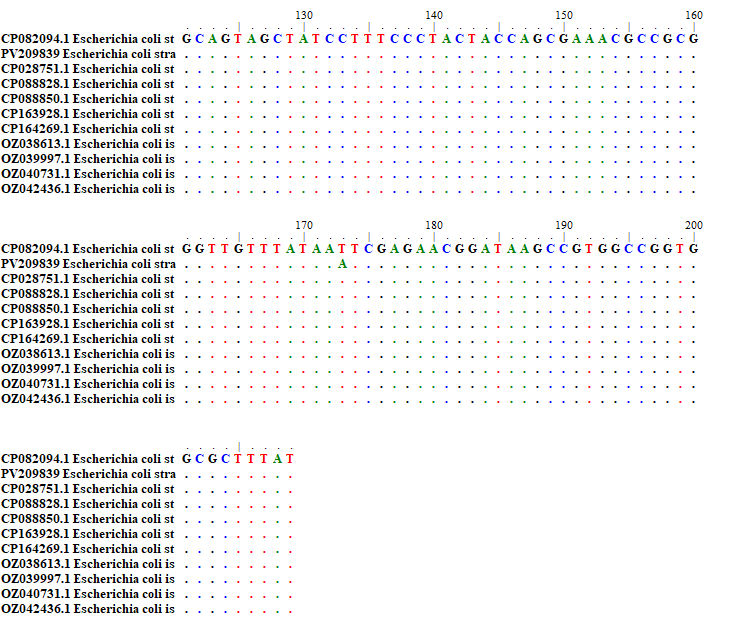


**Figure S40.** Nucleotide sequence alignments of the *fimH* gene of the examined *Escherichia coli* isolate strain S2 with the accession number of PV209839. The sequences are indicated by their accession numbers.


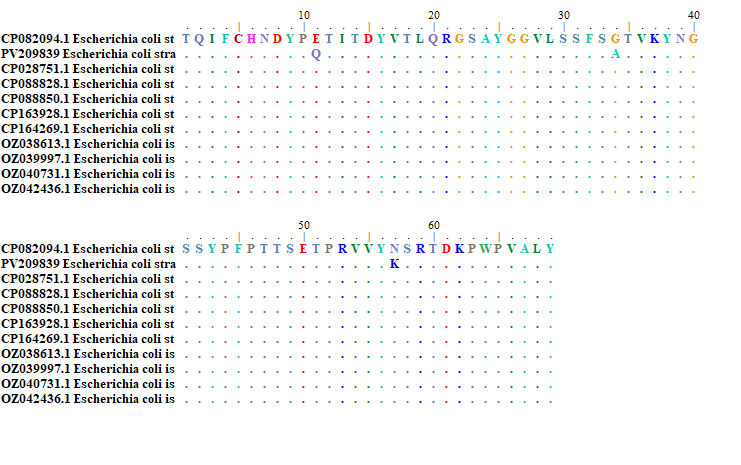
**Figure S41.** Amino acid sequence alignments of the *fimH* gene of the examined *Escherichia coli* isolate strain S2 with the accession number of PV209839. The sequences are indicated by their accession numbers.


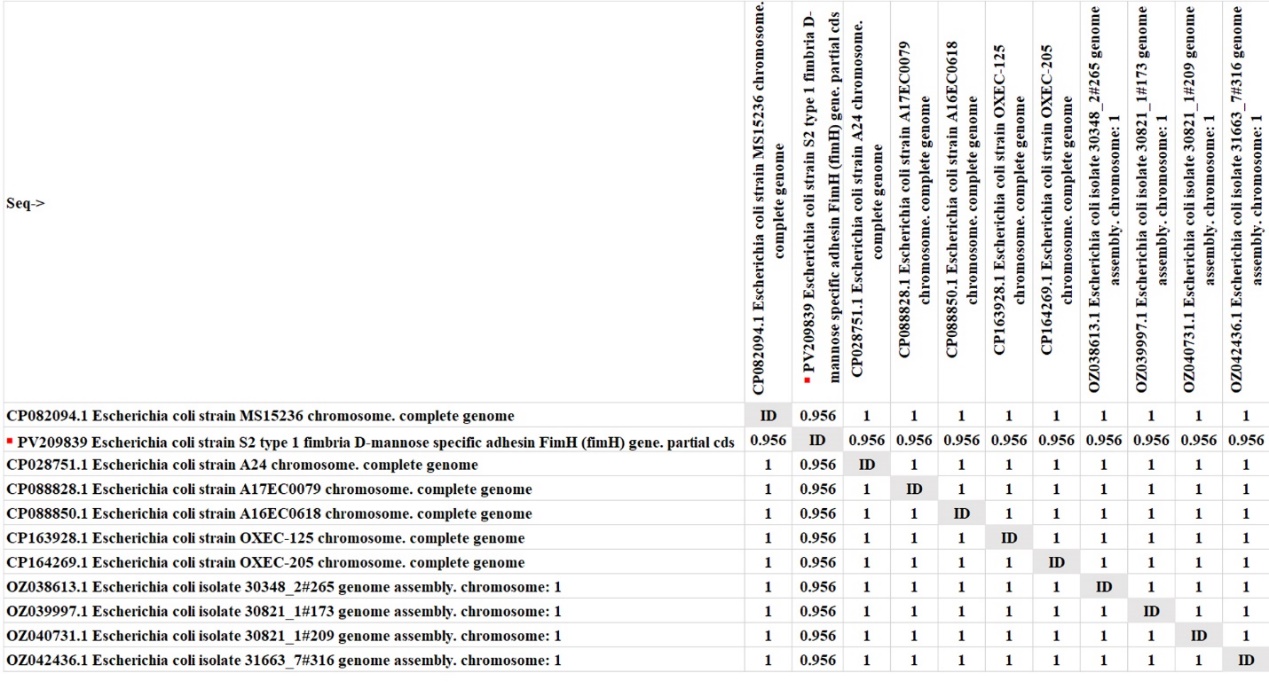


**Figure S42.** Amino acid identity percentages between the *fimH* genes of the examined *Escherichia coli* isolate strain S2, and other *E. coli* isolates on the GenBank. ID: identity. ▪ Our examined *E. coli* isolate.


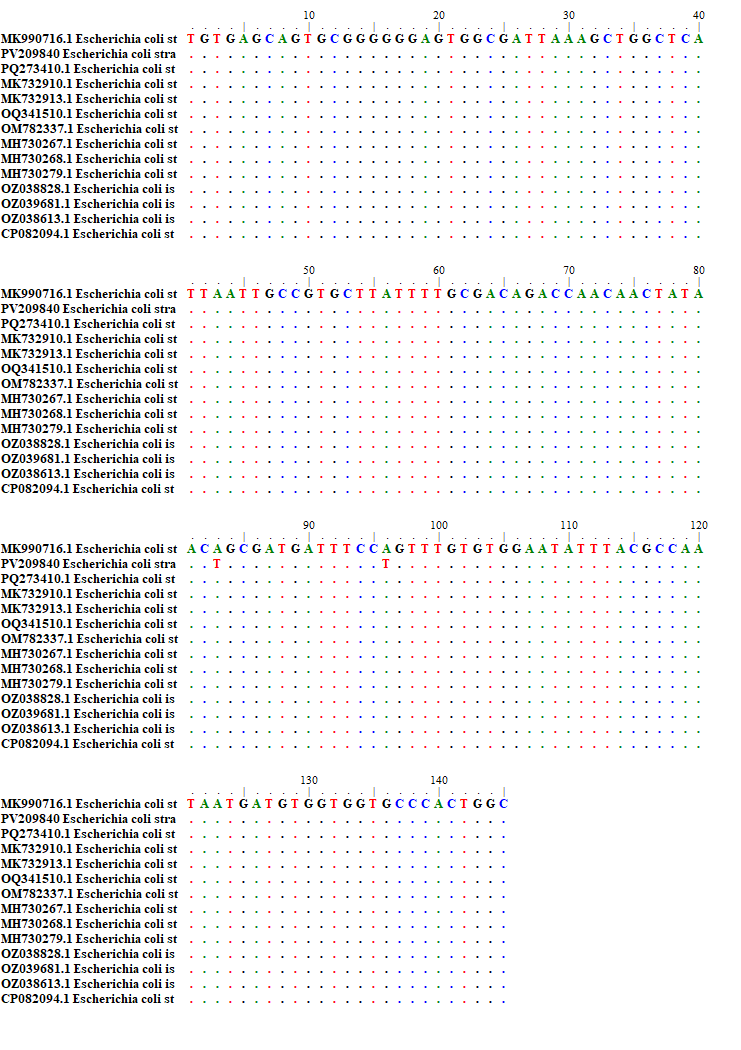


**Figure S43.** Nucleotide sequence alignments of the *fimH* gene of the examined *Escherichia coli* isolate strain S3 with a ccession number of PV209840. The sequences are indicated by their accession numbers.


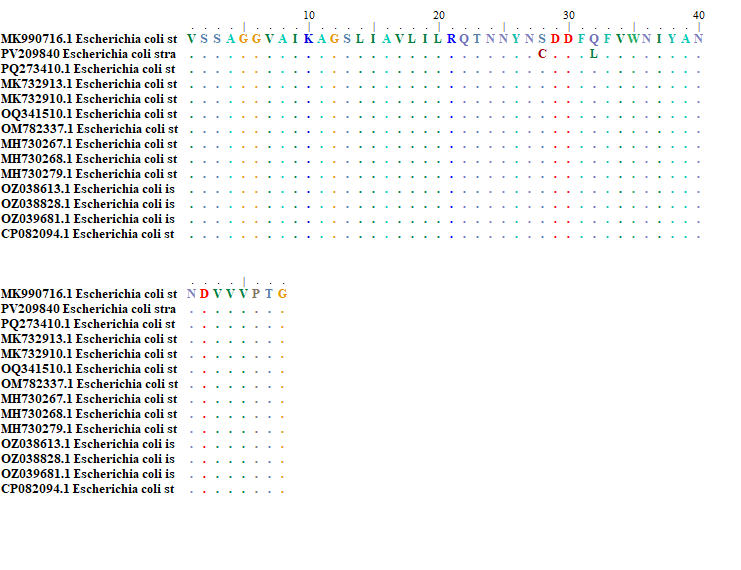


**Figure S44.** Amino acid sequence alignments of the *fimH* gene of the examined *Escherichia coli* isolate strain S3 with accession number of PV209840. The sequences are indicated by their accession numbers.


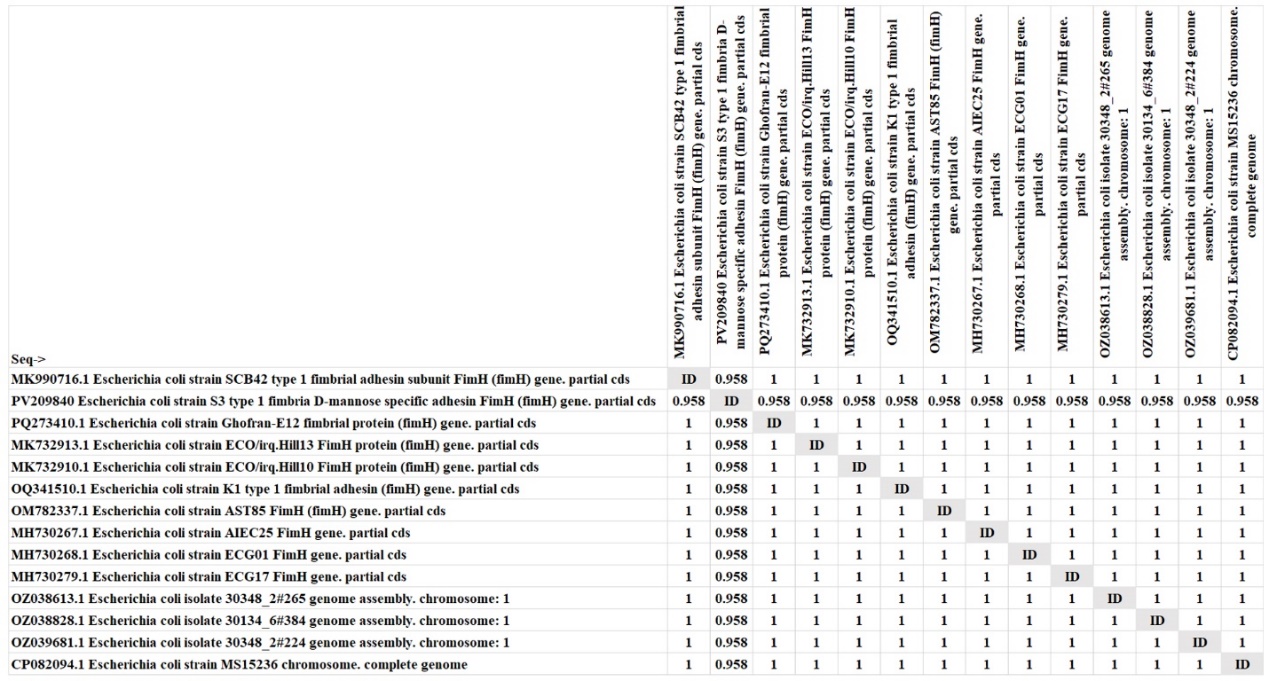


**Figure S45.** Amino acid identity percentages between the *fimH* genes of the examined *Escherichia coli* isolate strain S3, and other *E. coli* isolates on the GenBank. ID: identity. ▪ Our examined *E. coli* isolate.


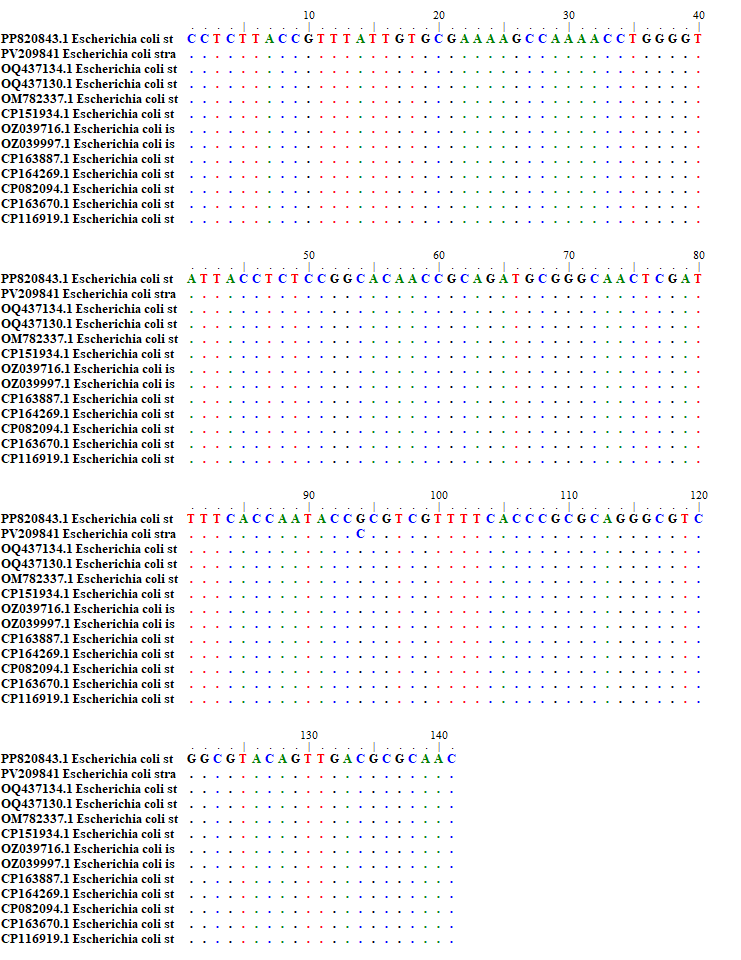


**Figure S46.** Nucleotide sequence alignments of the *fimH* gene of the examined *Escherichia coli* isolate strain S4 with accession number of PV209841. The sequences are indicated by their accession numbers.


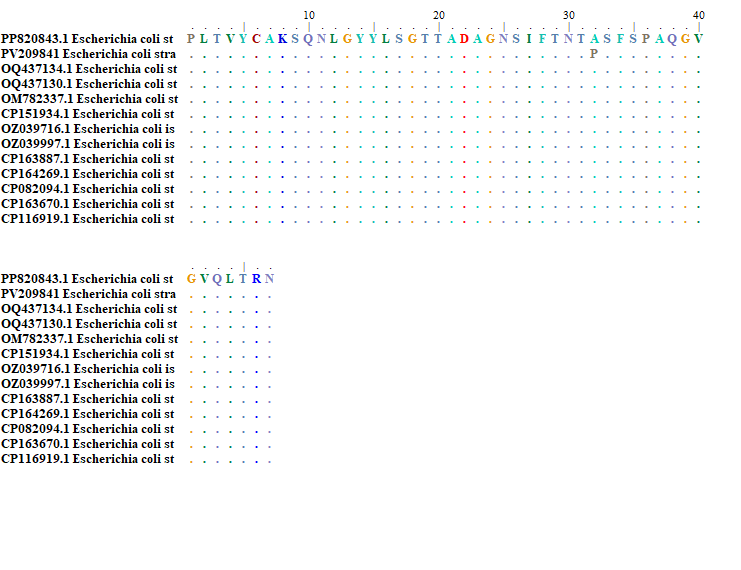


**Figure S47.** Amino acid sequence alignments of the *fimH* gene of the examined *Escherichia coli* isolate strain S4 with accession number of PV209841. The sequences are indicated by their accession numbers.


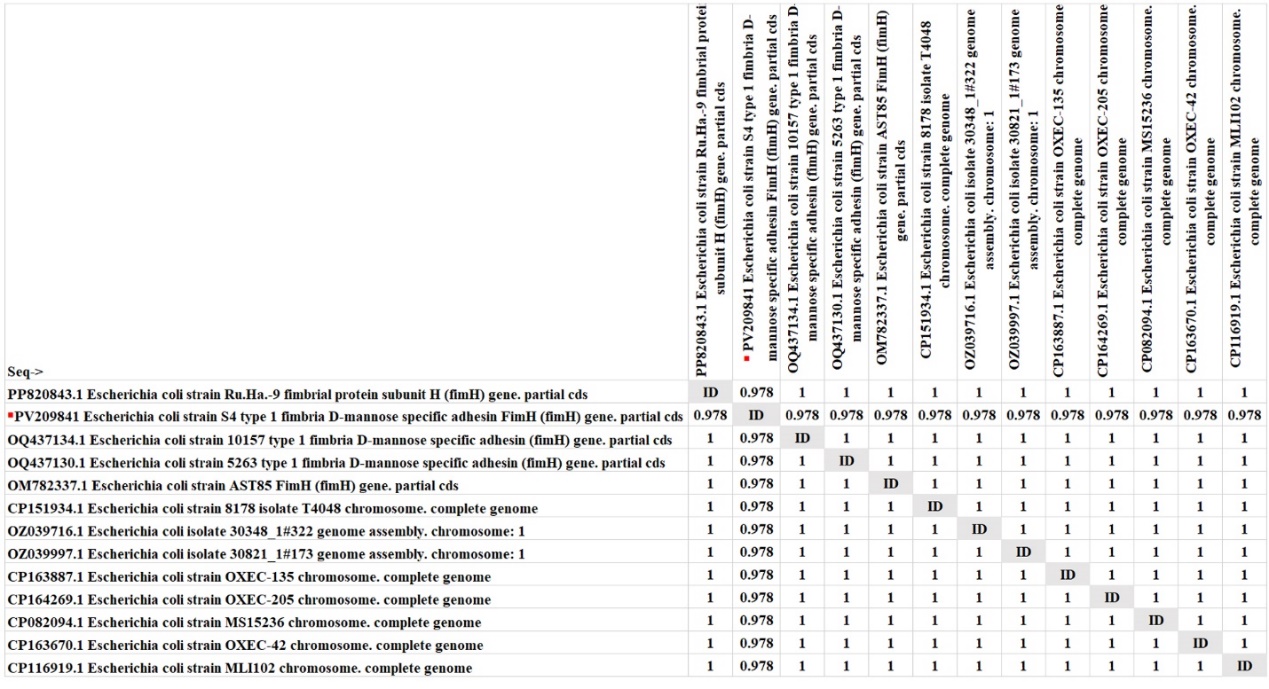


**Figure S48.** Amino acid identity percentages between the *fimH* genes of the examined *Escherichia coli* isolate strain S4, and other *E. coli* isolates on the GenBank. ID: identity. ▪ Our examined *E. coli* isolate.


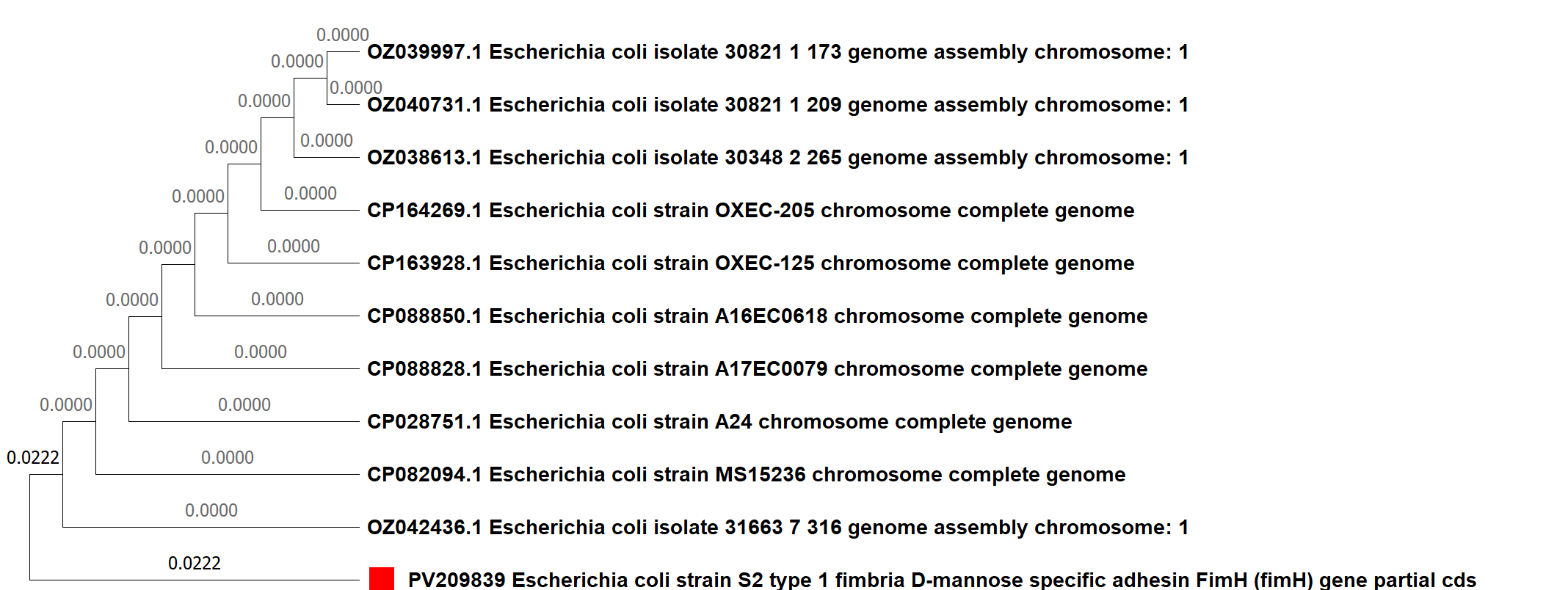


**Figure S49.** Phylogenetic tree of the examined *Escherichia coli* isolate strain S2 based on the *fimH* gene partial sequence generated via the Neighbor-Joining technique. ▪ Our examined *E. coli* isolate.


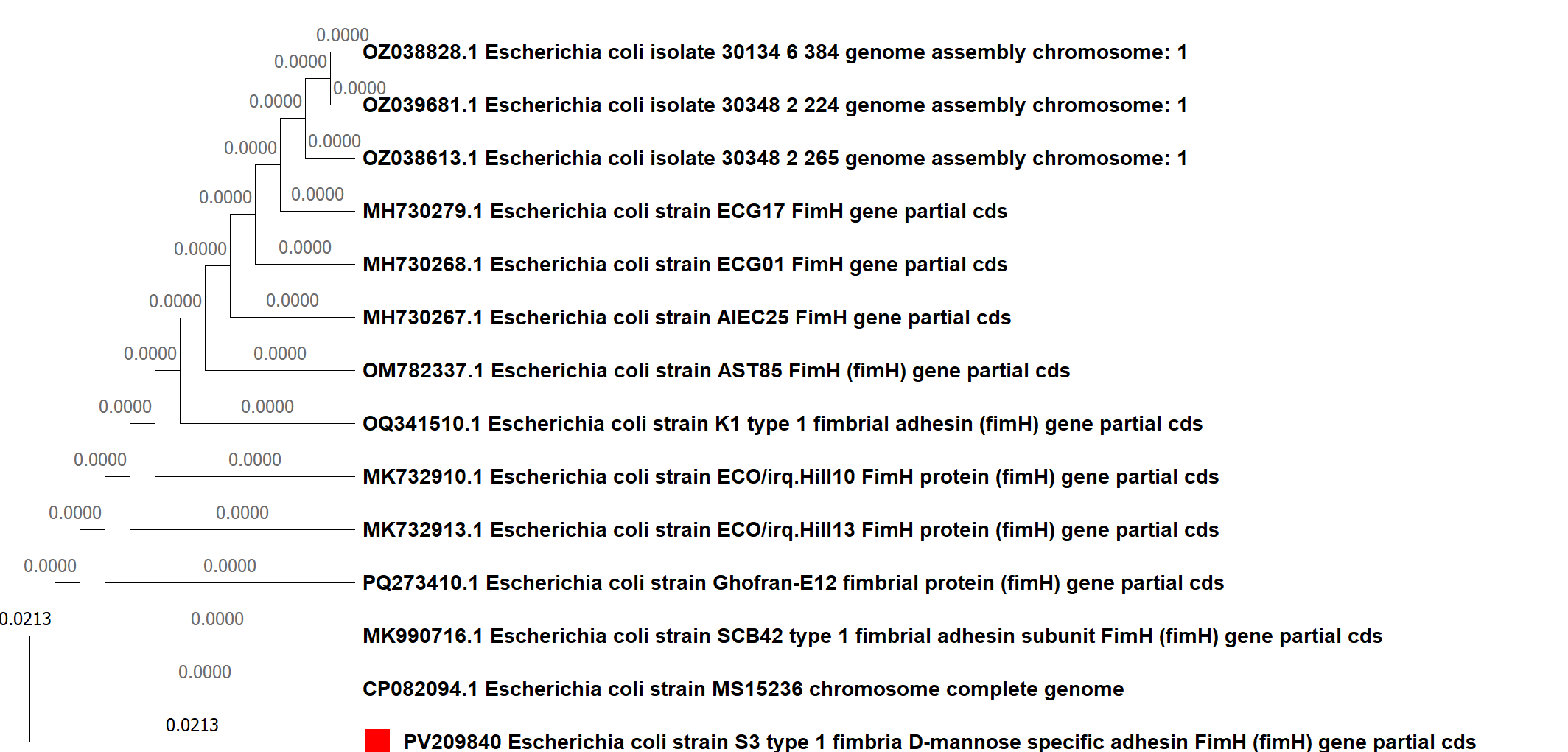


**Figure S50.** Phylogenetic tree of the examined *Escherichia coli* isolate strain S3 based on the *fimH* gene partial sequence generated via the Neighbor-Joining technique. ▪ Our examined *E. coli* isolate.


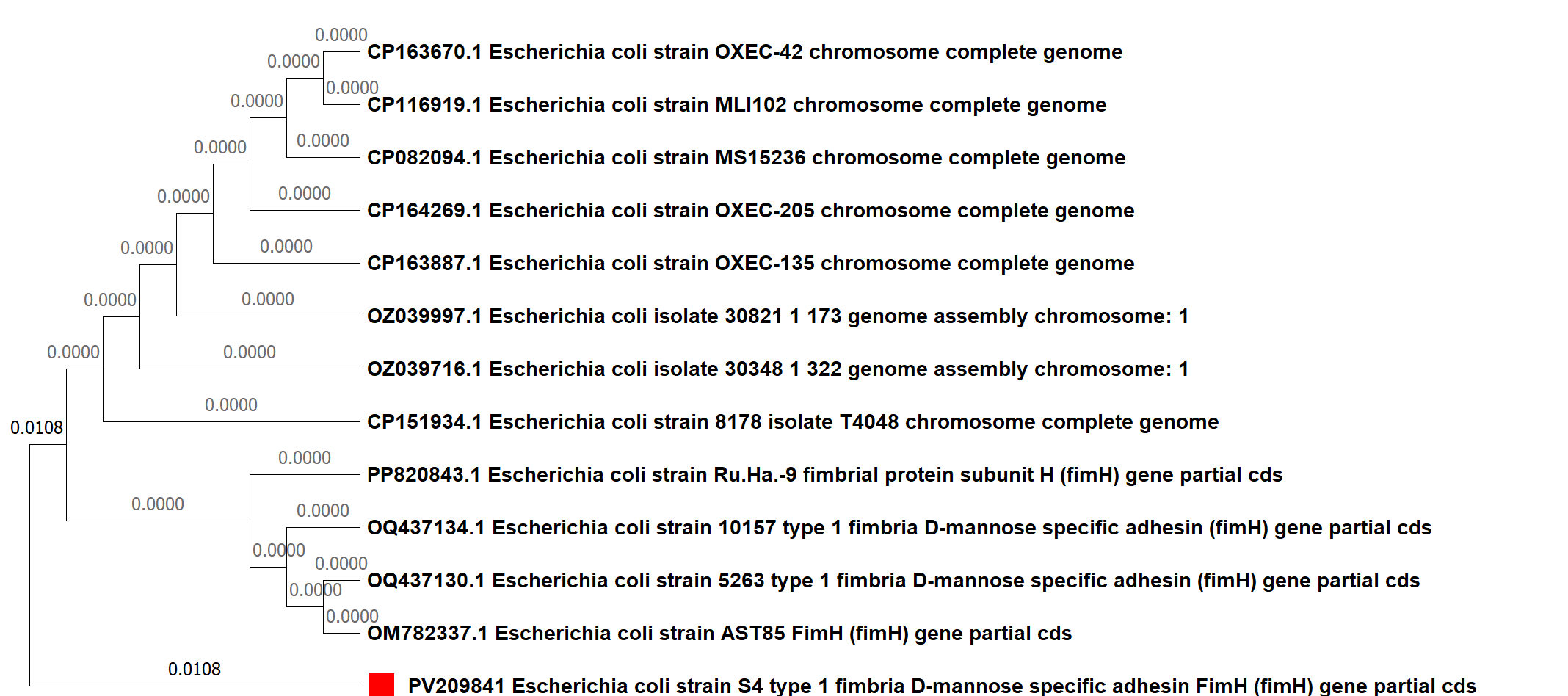


**Figure S51.** Phylogenetic tree of the examined *Escherichia coli* isolate strain S4 based on the *fimH* gene partial sequence generated via the Neighbor-Joining technique. ▪ Our examined *E. coli* isolate.


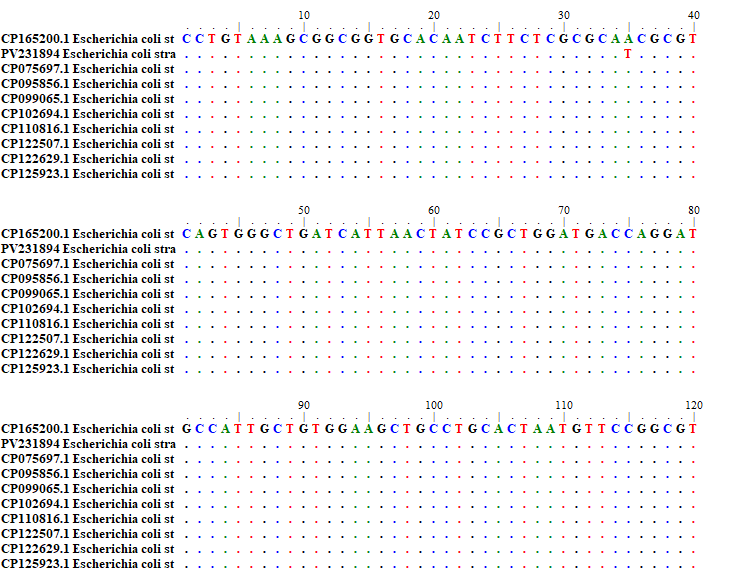

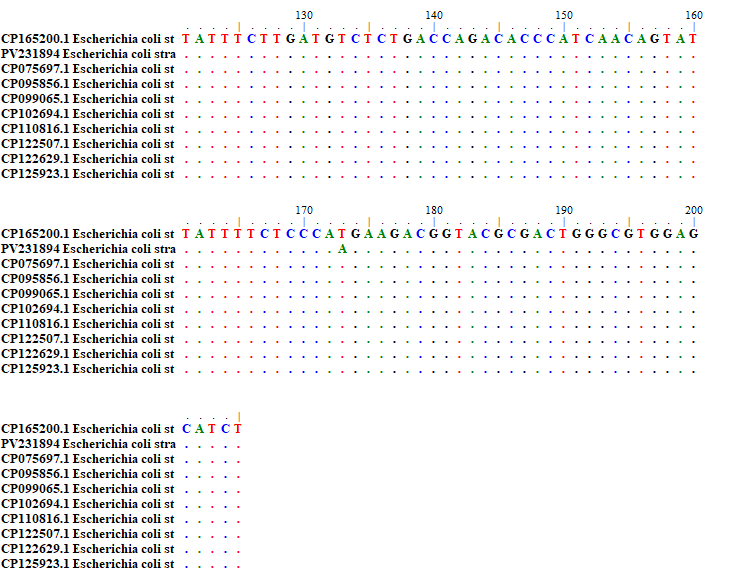


**Figure S52.** Nucleotide sequence alignments of the *lacI* gene of the examined *Escherichia coli* isolate strain S1 with accession number of PV231894. The sequences are indicated by their accession numbers.


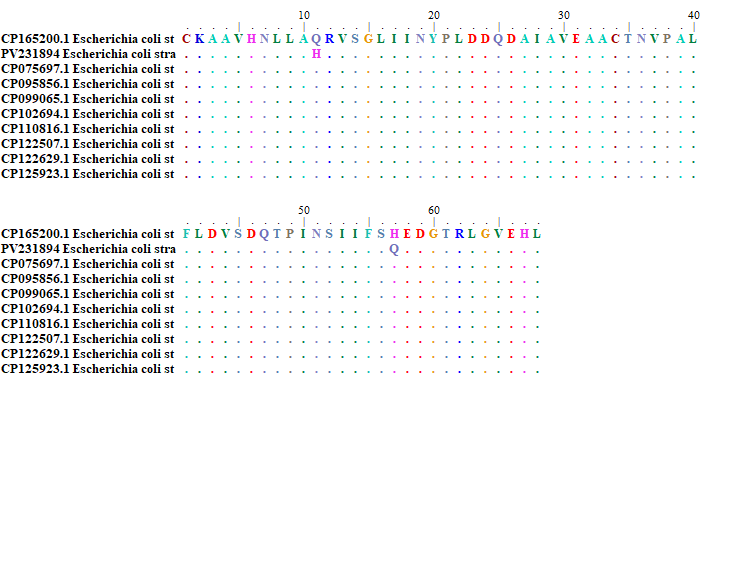


**Figure S53.** Amino acid sequence alignments of the *lacI* gene of the examined *Escherichia coli* isolate strain S1 with accession number of PV231894. The sequences are indicated by their accession numbers.


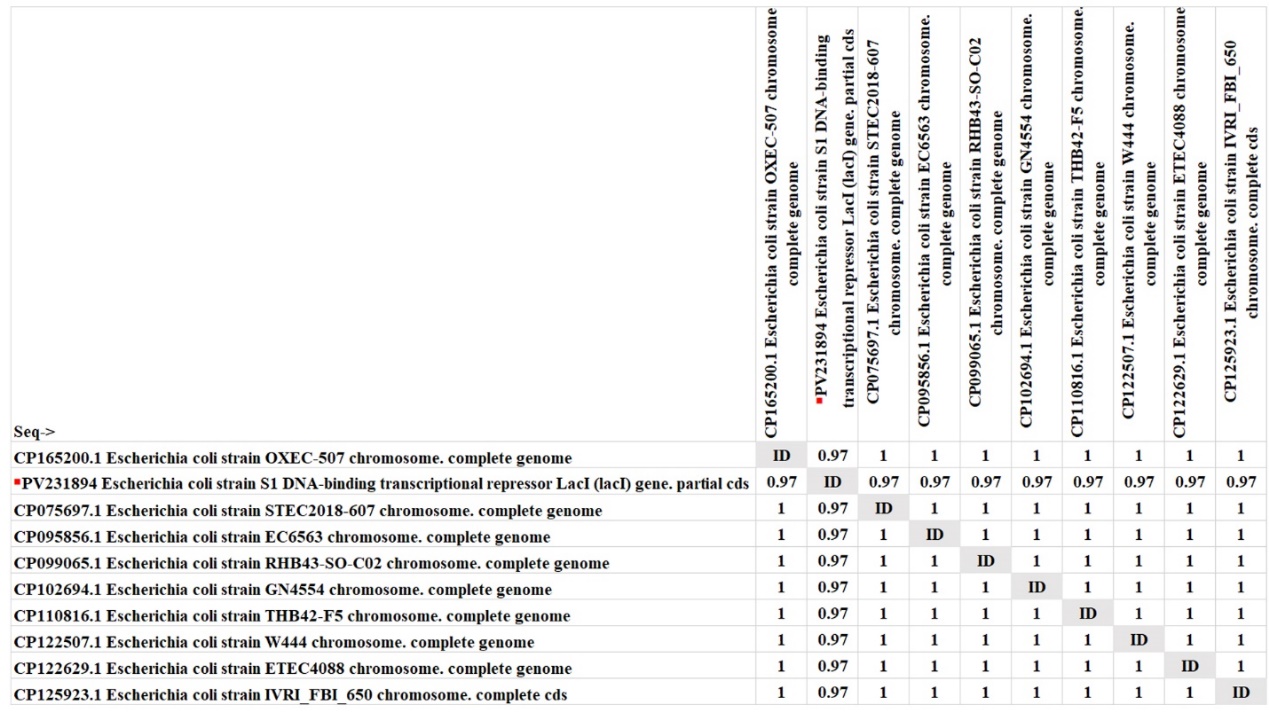


**Figure S54.** Amino acid identity percentages between the *lacI* genes of the examined *Escherichia coli* isolate strain S1, and other *E. coli* isolates on the GenBank. ID: identity. ▪ Our examined *E. coli* isolate.


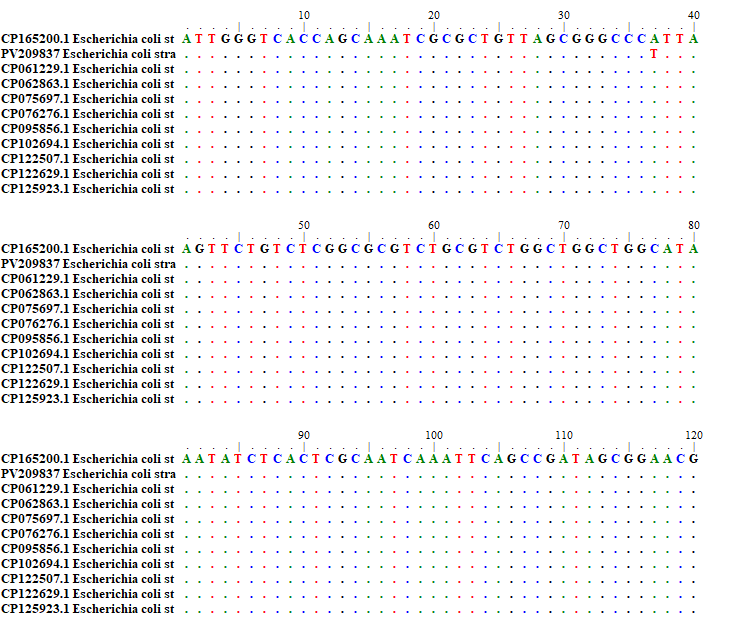

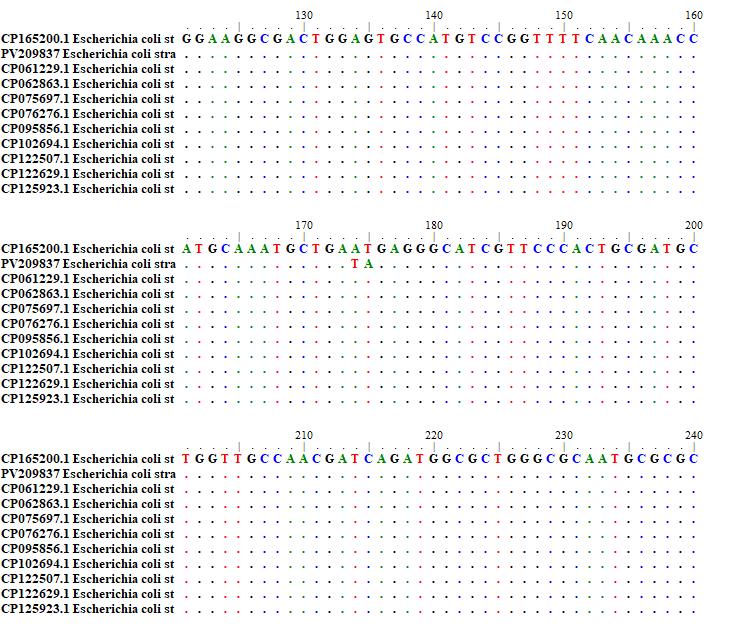


**Figure S55.** Nucleotide sequence alignments of the *lacI* gene of the examined *Escherichia coli* isolate strain S2 with accession number of PV209837. The sequences are indicated by their accession numbers.


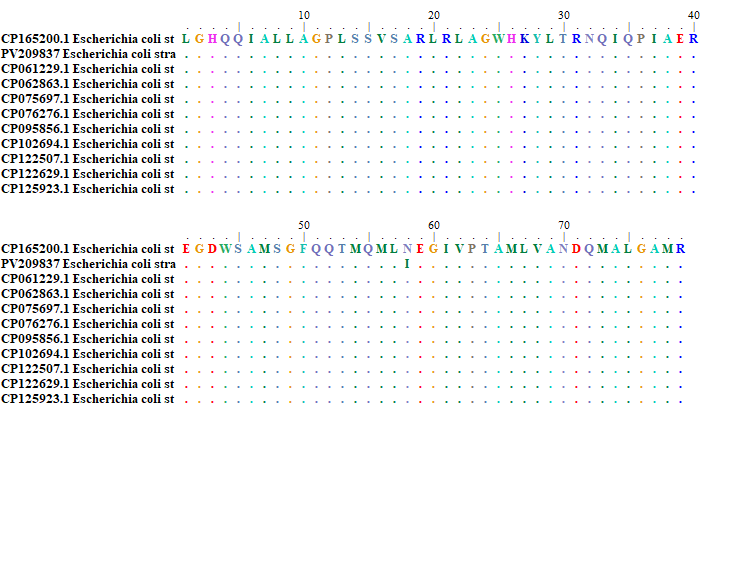


**Figure S56.** Amino acid sequence alignments of the *lacI* gene of the examined *Escherichia coli* isolate strain S2 with accession number of PV209837. The sequences are indicated by their accession numbers.


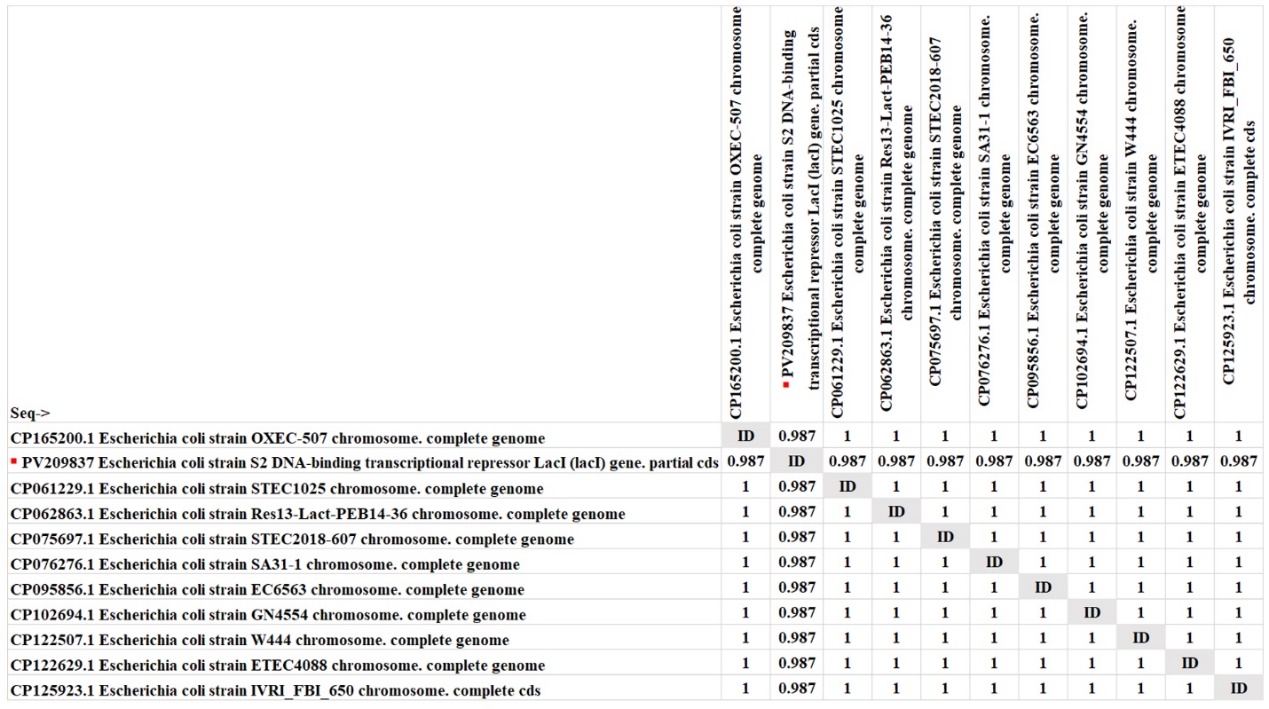


**Figure S57.** Amino acid identity percentages between the *lacI* genes of the examined *Escherichia coli* isolate strain S2, and other *E. coli* isolates on the GenBank. ID: identity. ▪ Our examined *E. coli* isolate.


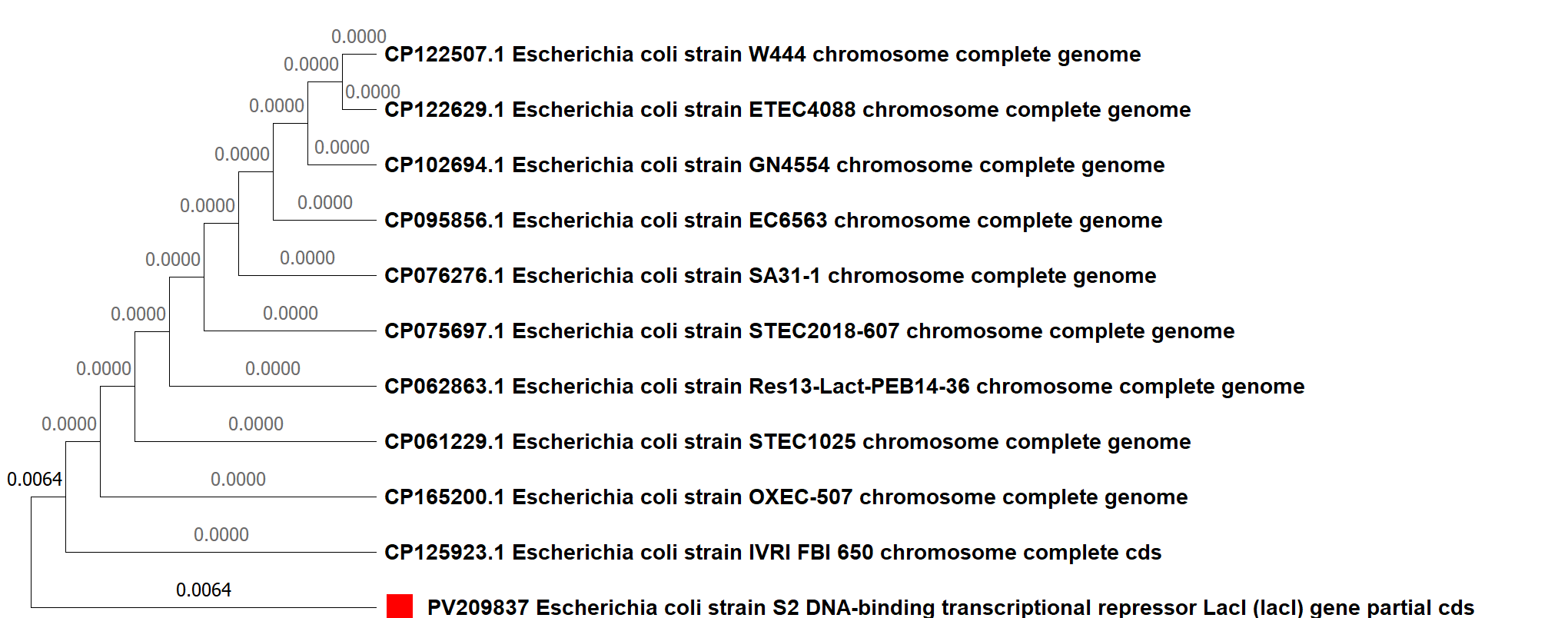


**Figure S58.** Phylogenetic tree of the examined *Escherichia coli* isolate strain S2 based on the *lacI* gene partial sequence generated via the Neighbor-Joining technique. ▪ Our examined *E. coli* isolate.


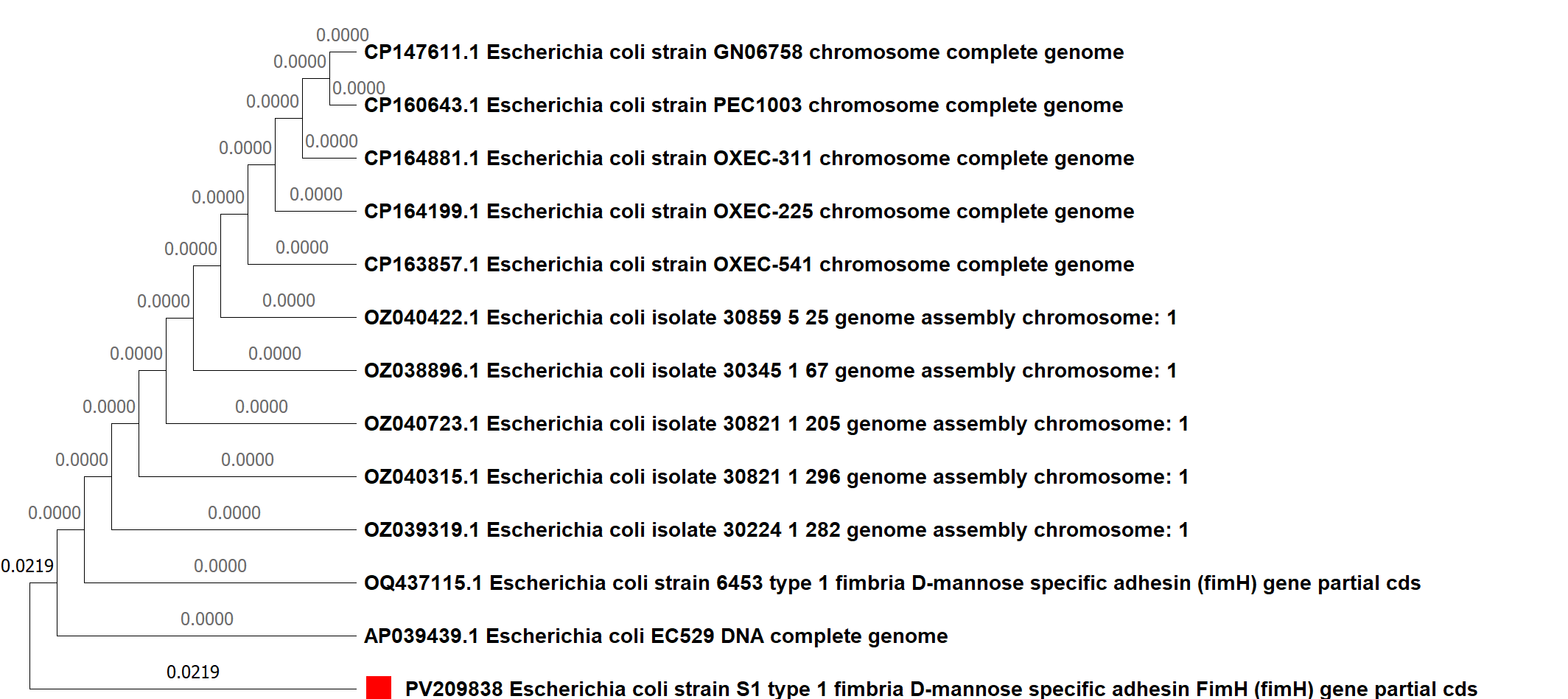


**Figure 59.** Phylogenetic tree of the examined *Escherichia coli* isolate strain S1 based on the *fimH* gene partial sequence generated via the Neighbor-Joining technique. ▪ Our examined *E. coli* isolate.

**
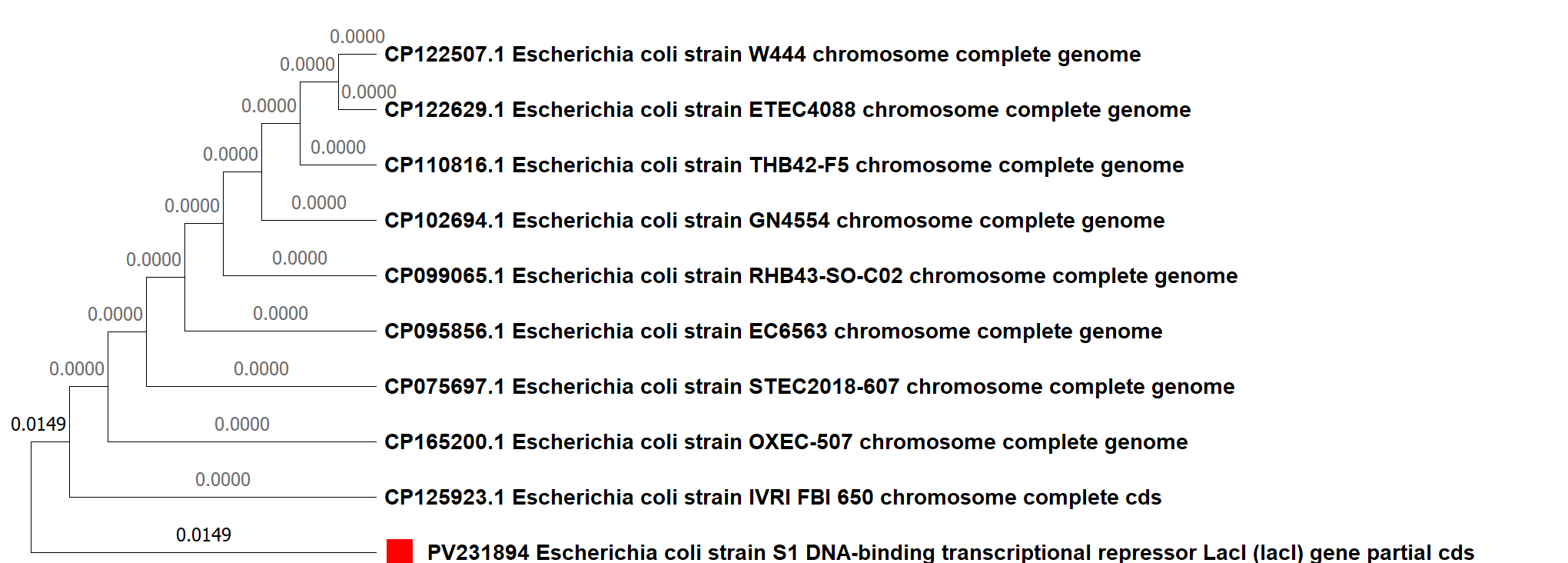
**

**Figure 60.** Phylogenetic tree of the examined *Escherichia coli* isolate strain S1 based on the *lacI* gene partial sequence generated via the Neighbor-Joining technique. ▪ Our examined *E. coli* isolate**.**
